# GSDME–IL-18 pyroptotic axis prevents myosteatosis by expanding tissue-resident macrophages to promote muscle regeneration

**DOI:** 10.1172/JCI198076

**Published:** 2026-02-17

**Authors:** Qi Cao, Jian Liu, Gang Huang, Su-Yuan Wang, Guo-Dong Lu, Yong Huang, Yi-Ting Chen, Zhen Zhang, Jiang-Tao Fu, Si-Jia Sun, Xiao-Fei Chen, Chunlin Zhuang, Chunquan Sheng, Fu-Ming Shen, Dong-Jie Li, Pei Wang

**Affiliations:** 1 Center for Basic Research and Innovation of Medicine and Pharmacy (MOE), School of Pharmacy, Naval Medical University, Shanghai, China.; 2 Department of Hepatic Surgery, Eastern Hepatobiliary Surgery Hospital, Naval Medical University, Shanghai, China.; 3 Department of Urology, Baoshan Renhe Hospital, Shanghai University, Shanghai, China.; 4 Department of Pharmacy, Shanghai Tenth People’s Hospital, School of Medicine, Tongji University, Shanghai, China.

**Keywords:** Immunology, Inflammation, Metabolism, Macrophages, Signal transduction

## Abstract

Metabolic–inflammatory crosstalk orchestrates muscle repair. Although pyroptosis typically aggravates sterile injury, we demonstrated that GSDME-dependent pyroptotic signaling associated with recruited myeloid cells paradoxically supported regeneration. GSDME expression was induced in postsurgical human muscle injury and murine damage models. *Gsdme* deficiency delayed functional recovery and exacerbated injury-induced myosteatosis, a pathological form of intramuscular ectopic fat deposition. Time-series and scRNA-seq analyses revealed that GSDME loss shifted the transcriptional program from oxidative metabolism to lipid storage and adipogenesis. Lipidomics confirmed aberrant accumulation of triacylglycerols (TAGs) and sphingolipids in *Gsdme*-deficient muscle. Single-cell profiling further identified divergent fibro-adipogenic progenitor (FAP) states skewed toward adipogenesis, accompanied by impaired expansion of restorative Lyve1^+^Cd163^+^Txnip^+^ tissue-resident macrophages (TRMs), as validated by multiplex flow cytometry. Blocking CCR2-dependent monocyte recruitment produced regenerative defects comparable with those caused by *Gsdme* deficiency. Myeloid-specific *Gsdme* reintroduction rescued TRM expansion and function and curbed FAP adipogenic reprogramming, whereas FAP-specific expression proved ineffective. Mechanistically, IL-18 downstream of GSDME-dependent signaling engaged KLF4/JUN signaling in TRMs, sustaining their reparative and lipid-clearing capacity. This GSDME–IL-18–TRM axis was compromised in aged muscle, yet exogenous IL-18 reversed myosteatosis and accelerated regeneration. Together, these findings suggest that GSDME-dependent pyroptotic signaling can act as a metabolic checkpoint that sustains TRM-driven lipid homeostasis to support muscle regeneration.

## Abstract 2

GSDME–IL18 axis-orchestrated pyroptosis in recruited macrophages establishes a pro-regenerative immune niche to sustain tissue-resident macrophage expansion, restrain fibro-adipogenic progenitor adipogenesis, and promote muscle regeneration.

## Graphical abstract

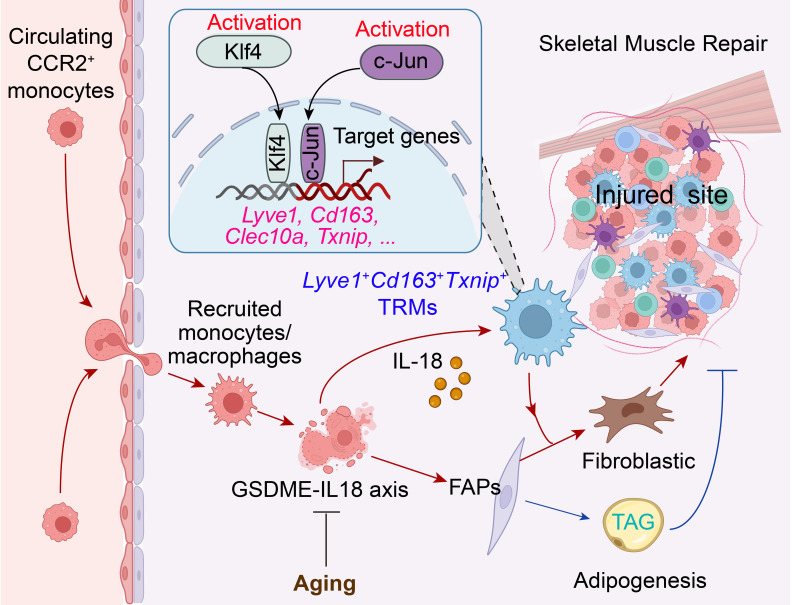

## Introduction

Skeletal muscle exhibits exceptional regenerative capacity after injury. While muscle satellite stem cells (MuSCs) drive myofiber regeneration, effective repair also depends on coordinated interactions among non-muscle cells, including immune cells and fibro-adipogenic progenitors (FAPs). Under homeostasis, immune cells are sparse in muscle but rapidly infiltrate after injury to promote regeneration and suppress myosteatosis, a pathological form of ectopic fat deposition within skeletal muscle. Impaired muscle repair is frequently associated with immune dysfunction and pathological myosteatosis (1). Among myeloid cells, monocytes and macrophages play a central role in muscle healing by clearing cellular debris, inhibiting FAP adipogenesis and shifting the intramuscular microenvironment from proinflammatory to antiinflammatory states (2–4). However, conditions like aging, diabetes, and muscle dysfunction lead to excessive fat infiltration and reduced regenerative capacity (5, 6). The molecular mechanisms linking immune regulation, metabolic reprogramming, and myosteatosis during muscle repair remain unknown.

Pyroptosis is a lytic and highly inflammatory form of regulated cell death, first characterized in macrophages during bacterial infection (7). This process is mediated by proteolytic cleavage of gasdermin family proteins (GSDMA–GSDME), whose N-terminal fragments oligomerize to form pores in the plasma membrane (and, in some contexts, the mitochondrial membrane), ultimately leading to cell rupture and release of inflammatory cytokines such as IL-1β and IL-18 (8). Among gasdermins, GSDMD is the best-characterized executor of canonical pyroptosis, being cleaved by inflammatory caspases (caspase-1/4/5 in humans and caspase-1/11 in mice) (9, 10). In contrast, GSDME can convert apoptotic cell death into pyroptosis when cleaved by caspase-3, representing a distinct molecular pathway (11, 12). While both GSDMD and GSDME have been implicated in various pathological conditions, their specific roles in tissue repair remain incompletely understood. Previously, GSDMD-mediated canonical pyroptosis was shown to promote ischemic tissue injury (13–15). However, 2 recent studies have indicated that GSDMD-mediated pore formation in the cell membrane functions as a secretory pathway for metabolites, including prostaglandin and epoxyeicosatrienoic acid, which subsequently activate tissue repair (16, 17). Notably, the 2 studies demonstrated that deletion of GSDMD did not impact the release of IL-1β and IL-18 during muscle healing nor did it affect the intramuscular proinflammatory or immune components in the injured tissue (16, 17). This suggests that GSDMD may not be the primary regulator of immune/inflammatory responses following muscle injury. However, it remains unknown whether other gasdermins, such as GSDME, play a role in skeletal muscle repair.

We and others have implicated GSDME in driving tissue injury (18–20). Based on these findings, we initially hypothesized that GSDME-mediated pyroptosis would exacerbate muscle damage and impair regeneration. However, contrary to this expectation, our comprehensive multiomics analyses — including longitudinal bulk RNA-seq, untargeted lipidomics, and scRNA-seq — demonstrated that GSDME deficiency impairs muscle recovery by promoting myosteatosis and disrupting the expansion of reparative tissue-resident macrophages (TRMs). These results suggest an unexpected protective role for GSDME-mediated pyroptosis in skeletal muscle regeneration.

## Results

### GSDME contributes to skeletal muscle injury and is required for muscle regeneration.

**Figure 1:**
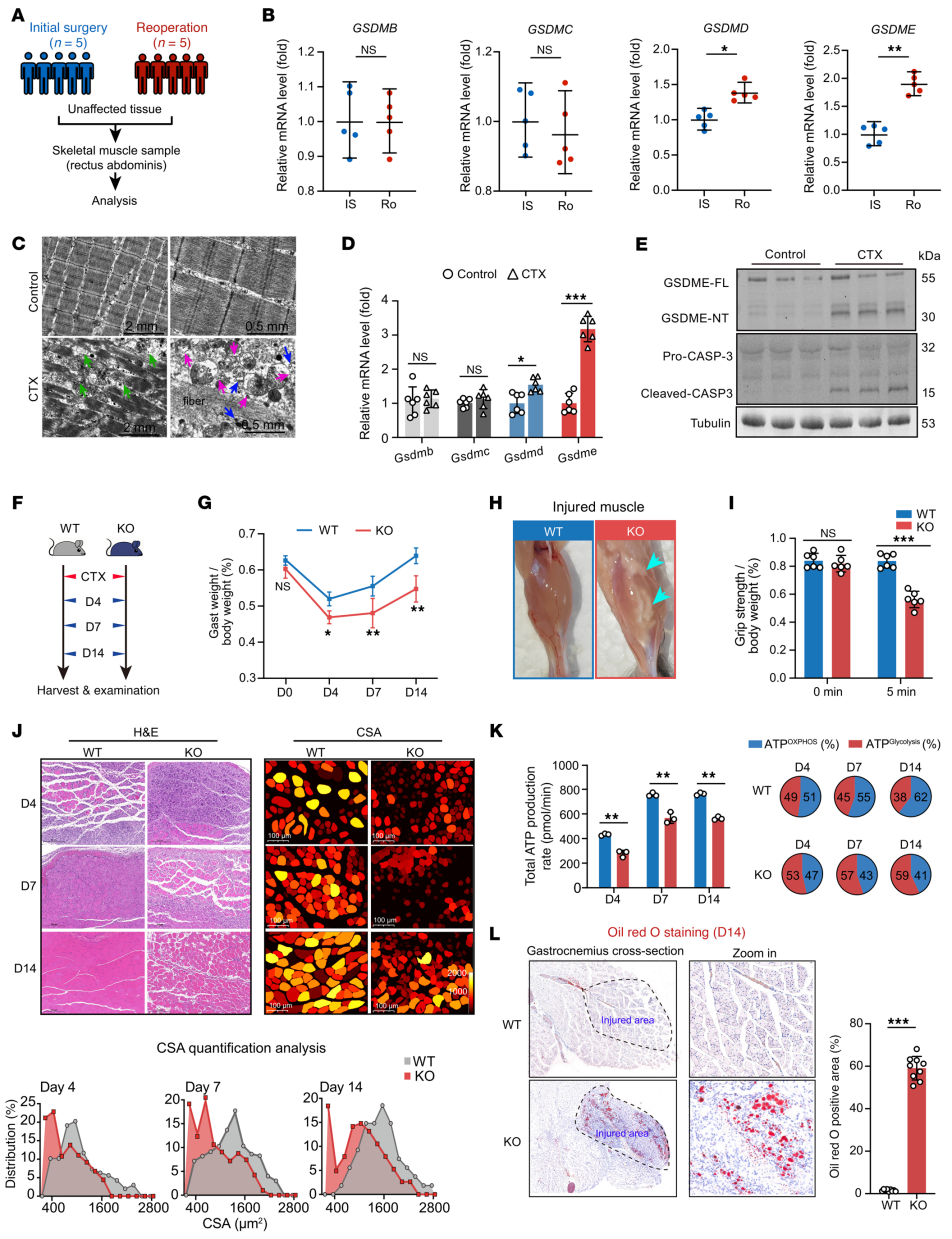
GSDME is activated in injured skeletal muscle, while its KO hinders tissue recovery by inducing dysmetabolism and fatty accumulation. (**A**) Scheme of GSDME analysis in rectus abdominis during surgical stress, comparing reoperation cases (5–8 days after initial biliary surgery for bile leakage, *n* = 5) with controls (initial biliary surgery, *n* = 5); all samples were histologically normal and unaffected by pathology. (**B**) Transcriptomic expression of gasdermin family members (GSDMB, GSDMC, GSDMD, and GSDME) in initial surgery (IS) versus reoperation (Ro) groups. (**C**) Transmission electron microscopy of CTX-injured skeletal muscle showing membrane pores and release of intracellular contents. Pink arrows: pore formation; blue arrows: released intracellular organelles; green arrows: cellular debris within the intercellular spaces of damaged muscle fibers. Scale bars: 2 mm (lower magnification), 0.5 mm (higher magnification). (**D**) Evaluation of gasdermin family members (*Gsdmb*, *Gsdmc*, *Gsdmd*, and *Gsdme*) in CTX-injured muscle of mice. (**E**) Evaluation of the cleavage of GSDME proteins in CTX-injured muscle of mice. (**F**) Experimental design for the CTX-induced muscle injury model and functional recovery in WT and GSDME-KO mice. (**G**) Gastrocnemius (Gast) weight/body weight ratio in WT and KO mice at D4, D7, and D14 after injury. Data are shown as the mean ± SD with *n* = 6 (from 6 WT independent mice) and *n* = 6 (from 6 *Gsdme*-KO independent mice). (**H**) Representative images of injured gastrocnemius in WT and KO mice. Cyan arrows indicate fatty degeneration. (**I**) Gastrocnemius strength at baseline (0 min) and 5 min later (5 min). (**J**) Representative images and quantification of CSA of myofibers stained with H&E. Scale bars: 100 μm. (**K**) Total ATP production and proportions of ATP produced via OXPHOS and glycolysis were calculated. (**L**) Representative images of Oil Red O staining and quantification of lipid accumulation. Scale bars, original images: 500 μm; enlargements: 200 μm. For **B**, **D**, and **L**, by unpaired 2-tailed Student’s *t* test; for **G**, **I**, and **K**, by 2-way ANOVA with Tukey’s post hoc test. Data are presented as mean ± SEM. *P < 0.05, **P < 0.01, ***P < 0.001.

To investigate the potential role of gasdermin family proteins in skeletal muscle during surgical stress, we analyzed rectus abdominis muscle samples obtained from 5 patients who required reoperation 5–8 days following initial biliary surgery due to bile leakage. Importantly, all cases involved only mild, localized leakage without abdominal wall infiltration, thereby ensuring that the collected muscle tissue was unaffected by the pathological process. Control samples comprised rectus abdominis muscle samples from 5 patients during initial biliary surgery, with all control tissues histologically normal. Among all gasdermin family members, *GSDME* mRNA showed the most pronounced upregulation in skeletal muscle (Figure 1, A and B). Based on this, we employed a cardiotoxin-induced (CTX-induced) muscle injury mouse model to study GSDME’s role. At day 4 (D4) after injury, H&E and succinate dehydrogenase (SDH) staining confirmed severe muscle damage (Supplemental Figure 1A; supplemental material available online with this article; https://doi.org/10.1172/JCI198076DS1), and electron microscopy revealed numerous cells undergoing membrane rupture near injured fibers (Figure 1C). Among gasdermin family members, *Gsdme* was the most upregulated in injured muscle (Figure 1D), with marked cleavage of GSDME protein and upstream caspase-3 (Figure 1E). Flow cytometry showed a substantial increase in CD45^+^ leukocytes (~10% to ~45%) and CD11b^+^ myeloid cells (6.4%–34.8%) in injured muscle (Supplemental Figure 1, B and C). IL-18 expression was elevated accordingly (Supplemental Figure 1D). O2K metabolic assay demonstrated reduced oxygen consumption in injured muscle (Supplemental Figure 1E). Bulk RNA-seq revealed upregulated genes enriched in inflammasome signaling, cytokine production, and leukocyte migration, while downregulated genes were related to muscle function and oxidative phosphorylation (Supplemental Figure 1, F and G). These data suggest a potential association between GSDME and skeletal muscle injury.

We then examined muscle repair in *Gsdme* global KO (Supplemental Figure 2, A and B) and WT mice at 3 postinjury time points (D4, D7, and D14; Figure 1F). By D14, WT controls had achieved complete recovery, while KO mice continued to show multiple pathological manifestations, including reduced gastrocnemius muscle mass normalized to body weight (Figure 1G), substantial lipid accumulation in injured muscle tissue (Figure 1H), and compromised grip strength (Figure 1I). To evaluate muscle regeneration, we performed H&E staining and quantitative analysis of myofiber cross-sectional area (CSA). Histological examination revealed more severe tissue disruption in GSDME-KO mice compared with WT controls (Figure 1J). This observation was further supported by CSA quantification using the Cellpose algorithm for cellular segmentation (21), which demonstrated a significantly reduced myofiber size in GSDME-KO mice following CTX-induced injury (Figure 1J), indicating impaired regenerative capacity in the absence of GSDME. KO mice also showed diminished running distance, time, and maximum speed at D14 (Supplemental Figure 2C). Immunostaining for SDH revealed fewer SDH^+^ fibers in KO muscle (Supplemental Figure 2D). Analysis of myosin heavy chain (MyHC) fiber types showed a consistent decrease in the ratio of oxidative type-IIa to glycolytic type-IIb fibers across time points (Supplemental Figure 2E), suggesting metabolic alterations. Seahorse assays demonstrated reduced oxygen consumption rate (OCR) and total ATP content, especially ATP from oxidative phosphorylation, in KO muscle (Supplemental Figure 2F). These findings were confirmed by luminescent ATP assays (Figure 1K). Oil Red O and perilipin-1 staining indicated increased fatty replacement in KO muscle (Figure 1L and Supplemental Figure 2G). MuSCs, marked by Pax7, are the principal cellular source for myofiber regeneration following injury. Notably, flow cytometry revealed comparable Pax7^+^ MuSC frequencies in uninjured (D0) WT and KO mice, with both genotypes exhibiting expected activation at D4 after CTX injury but no intergenotype difference (Supplemental Figure 2H), and qPCR analysis of *Pax7*, *Pax3*, and *Myf5* showed unchanged expression between genotypes at D0 and equivalent upregulation at D4 (Supplemental Figure 2I), suggesting that the impaired regeneration observed in KO mice is unlikely due to early MuSCs activation defects. Collectively, these results demonstrate that GSDME deficiency markedly impairs skeletal muscle repair process, potentially due to disrupted metabolic homeostasis and lipid accumulation.

### Bulk RNA-seq and lipidomics confirm GSDME loss-driven myosteatosis.

**Figure 2:**
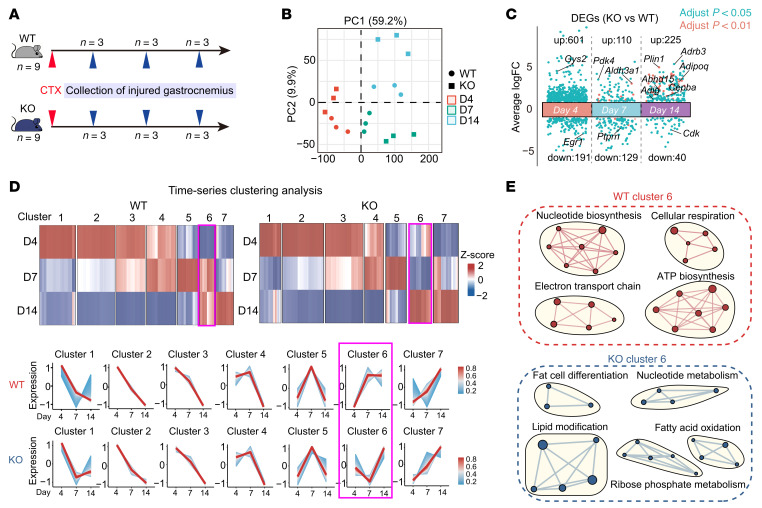
Time-series transcriptome analysis reveals that Gsdme deficiency enhances intramuscular adipogenesis. (**A**) Experimental design for time-series RNA-seq analyses of injured skeletal muscle tissue from WT and GSDME-KO mice with CTX-induced muscle injury at 3 postinjury time points (*n* = 3 per group at each time point). (**B**) Principal component analysis of transcriptome profiles in injured muscle of WT and KO mice. (**C**) Differentially expressed genes (DEGs) between injured muscle tissues of WT and KO mice at each time point. Cyan, adjusted *P* < 0.05; red, adjusted *P* < 0.01. (**D**) Time-series clustering analysis of transcriptomes using the Mfuzz algorithm in R. Cluster 6 gene signature (pink boxes) represents the most significant difference between WT and KO mice. (**E**) GO enrichment analysis demonstrating enriched signaling pathways in cluster 6 gene signatures of WT and KO mice.

To validate the association between impaired skeletal muscle repair and intramuscular lipid accumulation, we performed a time-series bulk RNA-seq analysis on CTX-injured skeletal muscles from WT and GSDME-KO mice at D4, D7, and D14 after injury (Figure 2A). Principal component analysis revealed distinct transcriptomic profiles across time points and genotypes (Figure 2B). Differentially expressed genes between KO and WT muscles are shown in Figure 2C and detailed in Supplemental Table 1. Using the Mfuzz algorithm, we identified 6 dynamic transcriptional clusters (clusters 1–5 and cluster 7) with similar temporal expression patterns in both groups, except for cluster 6, which displayed a distinct gene signature (Figure 2D and Supplemental Figure 3). Gene network analysis showed that cluster 6 encompassed regenerative processes in WT muscle, including myogenesis and anabolic metabolism, whereas in KO muscle, it was associated with adipogenic and lipid-remodeling pathways (Figure 2E). In KO muscle, adipogenic features emerged as early as D4 but subsided at D7, indicating an early, transient enrichment of adipogenic-associated transcriptional features in the context of sustained inflammatory signatures. By D14, these signatures became prominent and coincided with overt lipid accumulation (Figure 2, D and E). Thus, these findings support that GSDME deficiency induces myosteatosis during the late phase of muscle regeneration.

**Figure 3:**
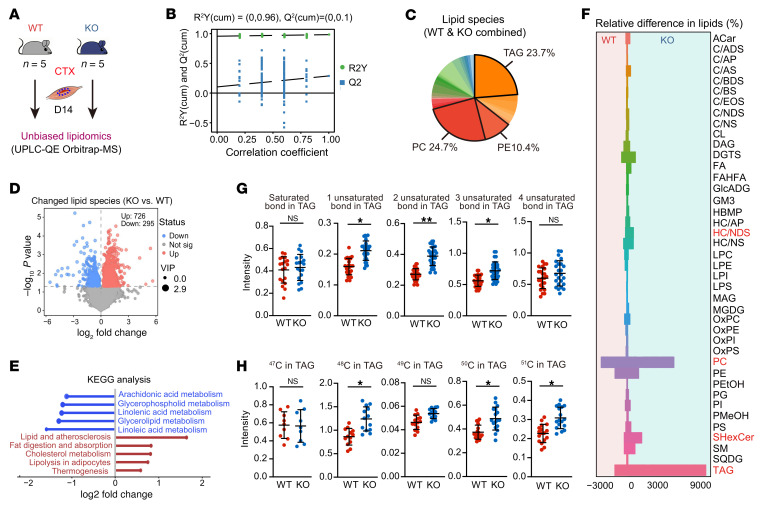
Lipidomics reveals increased intramuscular TAG enrichment in the absence of Gsdme. (**A**) Experimental design for untargeted lipidomics analysis using ultraperformance liquid chromatography–mass spectrometry in injured muscle of WT and KO mice at D14. (**B**) OPLS score plots assessing robustness of the mass spectrometry–based lipidomics model. (**C**) Lipid species identified in injured muscle of WT and KO mice: TAGs, PC, and phosphatidylethanolamine (PE). (**D**) Volcano plot showing the upregulated and downregulated lipid species in KO mouse muscle versus WT muscle. (**E**) KEGG pathway analysis of lipid-associated functions based on differential lipid species between WT and KO injured muscle; upregulated species are in red, and downregulated species are in blue. (**F**) Heatmap of differential lipid species in injured skeletal muscle from WT and KO mice. (**G**) Levels of saturated and unsaturated fatty acids in TAG species. (**H**) TAG levels of long-chain fatty acids with 47, 48, 49, 50, and 51 carbon atoms. Data are presented as mean ± SEM and were analyzed by unpaired 2-tailed Student’s *t* test. *P < 0.05, **P < 0.01.

To further understand the accumulated lipid species caused by GSDME deficiency, we performed a mass spectrometry–based untargeted lipidomic analysis (Figure 3A). The lipidomic data showed good separation and robust modeling (Figure 3B). Among lipid species identified, TAGs, phosphatidylcholine (PC), and phosphatidylethanolamine were the most abundant (Figure 3C). In KO muscle, 726 lipid species were upregulated, while 295 were downregulated (Figure 3D). KEGG pathway analysis indicated that the upregulated lipid species were primarily involved in lipid metabolism and fat digestion/absorption, whereas the downregulated lipid species were linked to linolenic acid and arachidonic acid metabolism (Figure 3E). Notably, TAG, PC, sulfur hexosylceramide hydroxy fatty acid (SHexCer), and hexosylceramide/non-hydroxy fatty acid-dihydrosphingosine (HC/NDS) were the most prominently upregulated lipid species in KO mice (Figure 3F). Saturated TAG levels were comparable between WT and KO mice, but levels of mono-, di-, and triunsaturated TAGs were significantly higher in KO muscle (Figure 3G). Among PC species, short-chain PCs showed no significant difference between WT and KO mice (Supplemental Figure 4A), whereas long-chain TAGs were significantly elevated in KO muscle (Figure 3H). The specific species of enhanced SHexCer and HC/NDS are detailed in Supplemental Figure 4, B and C. These lipidomic results confirm that GSDME deficiency exacerbates myosteatosis during the skeletal muscle regeneration from injury.

### scRNA-seq uncovers immune–stromal reprogramming in GSDME-KO muscle.

**Figure 4:**
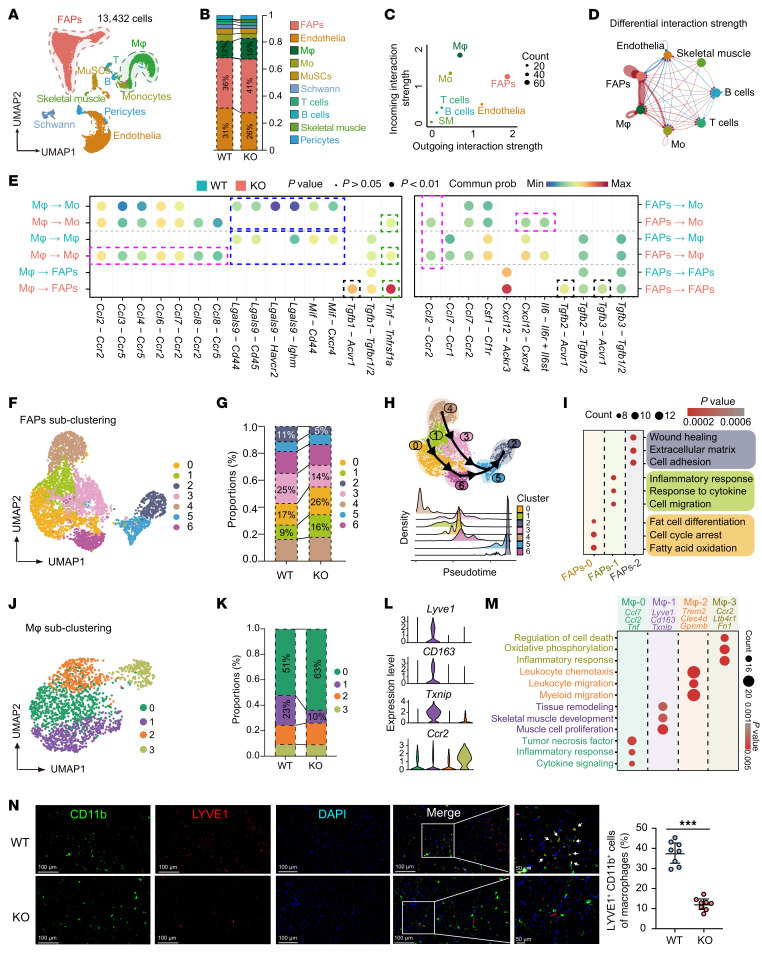
scRNA-seq reveals a coordinated regulation of GSDME in intramuscular TRMs and FAPs. (**A**) scRNA-seq UMAP plot showing clustering of endothelial cells, FAPs, macrophages, monocytes, MuSCs, Schwann cells, T cells, B cells, skeletal muscle (mature), and pericytes in the injured skeletal muscle tissue of WT and KO mice at D14. (**B**) Proportions of each cell cluster in injured skeletal muscle of WT and KO mice. (**C**) Analysis of incoming and outgoing interaction strength between the cell types in a 2-dimensional graph using the CellChat algorithm in R. (**D**) Circle plot showing the strength of ligand-receptor interactions between pairwise cell populations among the major cell populations in WT and KO groups. (**E**) Differences in ligand-receptor pairs among these cell types between WT and KO mice. (**F**) UMAP plot of FAP subgroups. (**G**) Proportions of each subcluster of FAPs in injured skeletal muscle of WT and KO mice. (**H**) Trajectory analysis of FAP differentiation according to pseudotime algorithms in injured skeletal muscle of WT and KO mice. (**I**) GO enrichment analysis of 3 crucial FAP subclusters between WT and KO muscle. (**J**) UMAP plot of macrophage subgroups. (**K**) Proportions of each subcluster of macrophages in injured skeletal muscle of WT and KO mice. (**L**) Expression of TRM and infiltrating macrophage marker genes across the 4 macrophage subclusters. (**M**) GO enrichment analysis showing the major biological functions enriched in the 4 subgroups of macrophages. (**N**) Multiplex IHC showing the proportion of Lyve1^+^CD11b^+^ macrophages within the total CD11b^+^ macrophage population in injured muscle (white arrows). Nuclei were stained with DAPI. Scale bars: 100 μm (lower magnification, 50 μm (higher magnification). Data are presented as mean ± SEM and were analyzed by unpaired 2-tailed Student’s *t* test. ***P < 0.001.

To illustrate the changes in cell status and molecular events within the injured and regenerated muscle, we used 10x Genomics scRNA-seq to study the injured muscle tissue (Supplemental Figure 5A). After quality control, 13,432 cells from the injured skeletal muscles of WT and KO mice were sequenced and clustered into endothelial cells, FAPs, macrophages, monocytes, MuSCs, Schwann cells, T cells, B cells, mature skeletal muscle, and pericytes (Figure 4A and Supplemental Figure 5, B and C). KO mice had a higher proportion of FAPs and macrophages (Figure 4B). When comparing the outgoing and incoming signals of each cell population in WT and KO mice, we found that FAPs and macrophages were the major signaling sources, and further analysis revealed a significantly strengthened FAP–macrophage interaction in KO mice (Figure 4, C and D). Enhanced IL-6, TRAIL, PAR, and TNF signaling pathways were present in KO muscle (Supplemental Figure 5D). Ligand-receptor analysis showed loss of *Mif*- and *Lgals9*-mediated interactions and induction of chemokine-, *Tgfb*-, and *Tnf*-mediated interactions in KO mice (Figure 4E).

As the regulation of macrophages on clearance of FAPs is an essential event for correct muscle repair, we further subgrouped FAPs and macrophages to detect their transcriptional changes. Seven FAP subgroups (Figure 4F and Supplemental Figure 5E) were identified: FAPs-0 (*Hsd11b1*^+^*Mme*^+^*Enpp2*^+^), FAPs-1 (*Ccl7*^+^*Ifi205*^+^*Fgl2*^+^), FAPs-2 (*Fmod*^+^*Wif1*^+^*Comp*^+^), FAPs-3 (*Sfrp1*^+^*Sfrp4*^+^*C2*^+^), FAPs-4 (*Pi16*^+^*Dpp4*^+^*Efemp1*^+^), FAPs-5 (*Thbs4^+^Tnmd^+^Chodl^+^*), and FAPs-6 (*Clu^+^Cpe^+^Myoc^+^*). According to the marker genes, FAPs-0 showed a high adipogenic differentiation trend (22–24), while FAPs-2 were fibroblast like (25, 26). Notably, KO mice had a higher proportion of adipogenic FAPs-0 but fewer fibroblast-like FAPs-2 (Figure 4G). Trajectory analysis suggested that *Pi16^+^Dpp4^+^* FAPs-4 might be the original state FAPs (27), while FAPs-2 were in a terminal state (Figure 4H and Supplemental Figure 5F). Gene Ontology (GO) enrichment analysis confirmed that FAPs-0 and FAPs-2 were involved in fat differentiation and wound healing, respectively (Figure 4I). The altered composition of FAP subgroups in KO mice indicates that *Gsdme* deletion drives adipogenic differentiation of FAPs.

Macrophages were subclustered into 4 groups (Figure 4J and Supplemental Figure 5G): Mφ-0 (*Ccl7*^+^*Tnf*^+^*Ccl2^+^*), Mφ-1 (*Lyve1*^+^*Cd163*^+^*Txnip*^+^), Mφ-2 (*Trem2*^+^*Clec4d*^+^*Gpnmb*^+^), and Mφ-3 (*Ccr2*^+^*Ltb4r1*^+^*Nlrp3*^+^). KO mice had increased Mφ-0 and decreased Mφ-1 compared with WT mice (Figure 4K). Mφ-0 seemed to be a group of high proinflammatory macrophages. Mφ-1, characterized by *Lyve1*, *Cd163*, and *Txnip* expression, were categorized as TRMs with high expansion capacity (28–31). Mφ-2 may be a group of lipid-sensing macrophage (32, 33). Mφ-3, marked by predominant *Ccr2* expression, were likely to be a group of macrophages derived from recruited monocytes (Figure 4L) (28–30). Pseudotime trajectory analysis suggested that Mφ-1 were in an independent terminal state distinct from Mφ-0, Mφ-2, and Mφ-3 (Supplemental Figure 5H), further supporting the unique gene signature of TRMs of Mφ-1. In line with these results, GO enrichment analysis revealed Mφ-1 was enriched in pathways related to tissue healing and skeletal muscle development, whereas Mφ-0, Mφ-2, and Mφ-3 were associated with inflammatory response and leukocyte migration (Figure 4M). Moreover, Mφ-3 was associated with regulation of cell death (Figure 4M). A prominently elevated expression level of transcription factors (TFs) such as Jun, Klf, Fos, and Egr1 was noted in Mφ-1 (Supplemental Figure 5I). Multiplex IHC staining revealed abundant LYVE1^+^CD11b^+^ TRMs within the injured tissue, whereas their presence was substantially diminished in KO mice (Figure 4N).

MuSCs were subclustered into 2 groups: Mu-0 (*Tpm3-rs7*^+^*Ndufa5*^+^*Cox7b*^+^) and Mu-1 (*Id3^+^Cebpd^+^Nfkbiz^+^*) (Supplemental Figure 5, J and K). WT mice had more Mu-0, which is involved in cellular respiration and oxidative phosphorylation (OXPHOS), whereas KO mice had more Mu-1, which is associated with the negative regulation of phosphate and phosphorus metabolic processes (Supplemental Figure 5L). Deletion of GSDME did not significantly impact the status of endothelial cells (Supplemental Figure 5, M–O).

All these findings indicate that GSDME may coordinately regulate immune–stromal reprogramming during muscle regeneration.

### GSDME in macrophages, but not FAPs, is essential for skeletal muscle regeneration.

To determine the functional relevance of GSDME in specific cell types during muscle regeneration, we analyzed its expression pattern across the major cells in muscle via our scRNA-seq data. We found *Gsdme* was preferentially expressed in FAPs and macrophages but showed low expression in MuSCs and endothelial cells (Supplemental Figure 6A). Flow cytometry analysis of GSDME protein levels confirmed this cell type–specific expression pattern, showing significantly elevated mean fluorescence intensity specifically in FAP and macrophage populations (Supplemental Figure 6B). Since scRNA-seq demonstrated GSDME’s dual regulation of FAP differentiation and macrophage reprogramming during muscle repair, we subsequently sought to determine the principal cell type responsible for GSDME’s essential role in skeletal muscle repair.

**Figure 5:**
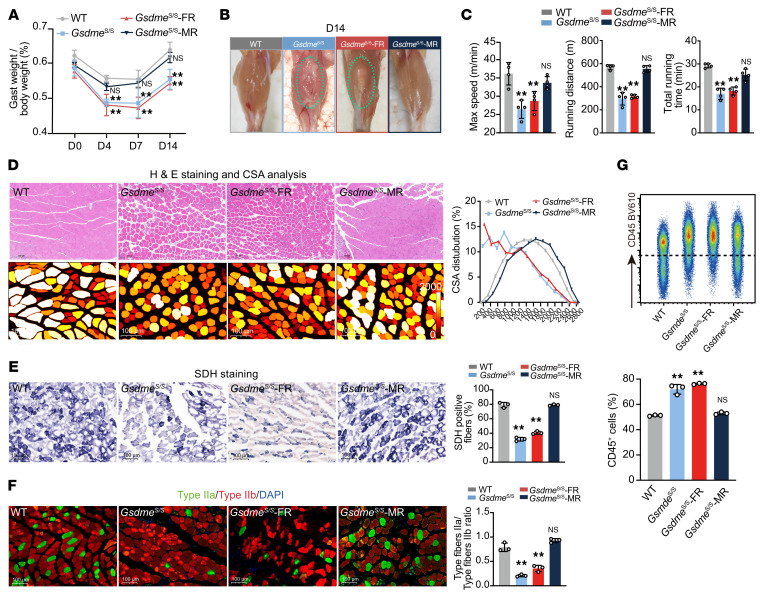
GSDME in recruited macrophages, but not in FAPs, is essential for skeletal muscle repair. (**A**) The gastrocnemius (Gast) weight/body weight ratio in WT (*Gsdme*^WT/WT^), *Gsdme*^S/S^ (*Gsdme*^Stop/Stop^), *Gsdme*^S/S^-FR (*Gsdme*^Stop/Stop^ *Pdgfra*-Cre), and *Gsdme*^S/S^-MR (*Gsdme*^Stop/Stop^ *Lysm*-Cre) mice at D4, D7, and D14 after injury. (**B**) Representative morphology of injured muscle of WT, *Gsdme*^S/S^, *Gsdme*^S/S^-FR, and *Gsdme*^S/S^-MR mice at D14 after injury. (**C**) Exercise performance of WT, *Gsdme*^S/S^, *Gsdme*^S/S^-FR, and *Gsdme*^S/S^-MR mice at D14 after injury. (**D**) Representative H&E staining and quantitative CSA analysis of injured muscle from WT, *Gsdme*^S/S^, *Gsdme*^S/S^-FR, and *Gsdme*^S/S^-MR mice at D14 after injury. Scale bars: 100 �m. (**E**) Representative SDH staining and quantitative analysis of injured muscle from WT, *Gsdme*^S/S^, *Gsdme*^S/S^-FR, and *Gsdme*^S/S^-MR at D14 after injury. Scale bars: 100 �m. (**F**) Representative immunofluorescence images of MyHC type-IIa and MyHC type-IIb fibers in injured muscle of WT, *Gsdme*^S/S^, *Gsdme*^S/S^-FR, and *Gsdme*^S/S^-MR at D14 after injury. Scale bars: 100 �m. (**G**) Proportion of CD45^+^ cells in injured muscle of WT, *Gsdme*^S/S^, *Gsdme*^S/S^-FR, and *Gsdme*^S/S^-MR at D14 after injury determined by flow cytometry. Data are presented as the mean ± SEM and were analyzed by 1-way ANOVA with Tukey’s post hoc tests. **P < 0.01.

A mouse strain (*Gsdme*^Stop/Stop^; Supplemental Figure 6C) described in our previous work (18, 19, 34) carrying a transcriptional STOP cassette (3×SV40-PolyA) flanked by loxP sites (loxP-Stop-loxP) upstream of the ATG start codon of the *Gsdme* gene was crossed with *Pdgfra*-Cre or *Lysm-*Cre mice to generate *Gsdme*^S/S^ *Pdgfra*-Cre (referred to as *Gsdme*^S/S^*-*FR) or *Gsdme*^S/S^ *Lysm*-Cre mice (referred to as *Gsdme*^S/S^*-*MR) mice, respectively. This strain enabled tissue-specific reactivation of Gsdme in FAPs or myeloid cells under a systemic Gsdme-deficient background (Supplemental Figure 6D), as confirmed by qPCR and immunoblotting (Supplemental Figure 6, E and F). WT (*Gsdme*^WT/WT^), *Gsdme*^S/S^, *Gsdme*^S/S^*-*FR, and *Gsdme*^S/S^*-*MR mice were subjected to CTX-induced skeletal muscle injury. Similar to GSDME-KO mice, *Gsdme*^S/S^ and *Gsdme*^S/S^-FR mice showed significantly reduced gastrocnemius weight, while *Gsdme*^S/S^-MR mice had comparable weight as WT mice (Figure 5A). *Gsdme*^S/S^ and *Gsdme*^S/S^-FR mice exhibited obvious fatty replacement in injured muscle (Figure 5B) and compromised running performance (Figure 5C), unlike *Gsdme*^S/S^-MR mice (Figure 5, B and C). Similar results were found in H&E staining/CSA analysis (Figure 5D), SDH staining (Figure 5E), and type-IIa/type-IIb myofiber ratio (Figure 5F). The number of CD45^+^ leukocytes increased in *Gsdme*^S/S^ and *Gsdme*^S/S^-FR mice, but to a lesser extent in *Gsdme*^S/S^-MR mice (Figure 5G).

**Figure 6:**
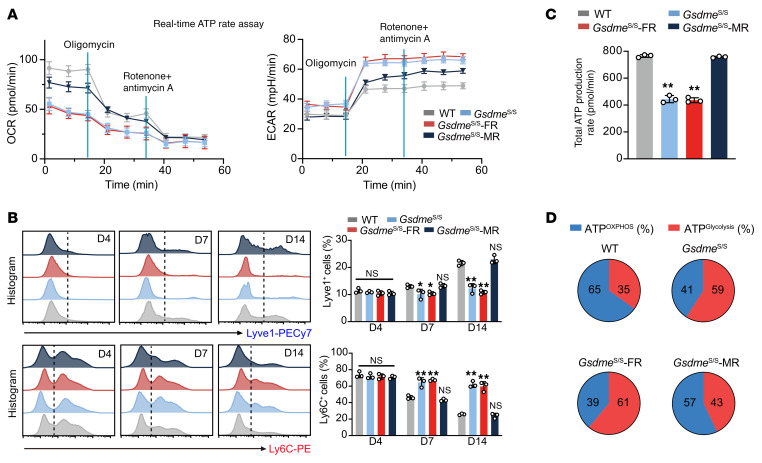
Gsdme regulates metabolic remodeling and macrophage polarization during muscle repair. (**A**) Analysis of the metabolic profile of injured skeletal muscle tissue of WT, *Gsdme*^S/S^, *Gsdme*^S/S^-FR, and *Gsdme*^S/S^-MR at D14 after injury using Seahorse technology. The curves for OCR and ECAR are presented. (**B**) Time-series analyses of proportions of Ly6C^+^ or LYVE1^+^ macrophages within the CD11b^+^ macrophages/monocytes of injured skeletal muscle tissue. (**C** and **D**) Total ATP production and the proportions of ATP produced via 2 distinct manners (OXPHOS and glycolysis) of injured skeletal muscle tissue. Data are presented as the mean ± SEM and were analyzed by 1-way ANOVA with Tukey’s post hoc tests. *P < 0.05, **P < 0.01.

We next asked whether these impairments were associated with altered cellular metabolism and myeloid cell kinetics. Seahorse metabolic analysis showed lower OCR curves in muscle samples from *Gsdme*^S/S^ and *Gsdme*^S/S^-FR mice, but not in *Gsdme*^S/S^-MR mice, compared with WT mice (Figure 6A). Time-sequential changes of Ly6C^+^ monocytes and LYVE1^+^ macrophages in injured muscle showed that the rapid peaking of infiltrating Ly6C^+^ monocytes and the persistent presence of LYVE1^+^ macrophages in WT mice were absent in *Gsdme*^S/S^ and *Gsdme*^S/S^-FR mice but restored in *Gsdme*^S/S^-MR mice (Figure 6B). Total ATP content and the proportion of ATP produced by OXPHOS or glycolysis displayed similar changes (Figure 6, C and D). These findings suggest that GSDME in macrophages, but not FAPs, regulates skeletal muscle repair.

To delineate the cellular basis of pyroptosis, we investigated whether TRMs or recruited macrophages represent the predominant population undergoing this process during muscle repair. Analysis of our scRNA-seq dataset revealed selective enrichment of *Gsdme* and its downstream effectors (*Il18* and *Il1β*) in the Mφ-3 subset (CCR2^+^ recruited macrophages), but not in Mφ-0 (Lyve1^+^ TRMs), suggesting a preferential propensity toward GSDME-mediated pyroptotic activation in recruited macrophages (Supplemental Figure 6G). To validate this, we isolated CD11b^+^CCR2^+^ (recruited) and CD11b^+^CCR2^–^ (nonrecruited/TRMs) macrophages from injured muscles at D14 after injury using FACS (Supplemental Figure 6H). Flow cytometry analysis revealed significantly higher expression of pyroptosis-associated molecules, including GSDME, IL-18, and IL-1β, in recruited CD11b^+^CCR2^+^ monocyte-derived macrophages compared with CD11b^+^CCR2^–^ cells (Supplemental Figure 6I).

To further functionally assess their role in driving the GSDME–IL-18 axis, we employed the CCR2/5 dual antagonist cenicriviroc (CVC) to block monocyte recruitment. CVC treatment in normal mice for 2 consecutive weeks (Supplemental Figure 7A) did not exert direct cytotoxic effects on monocytes (Supplemental Figure 7B), consistent with previous findings (35). Upon muscle injury (Supplemental Figure 7C), CVC administration significantly reduced the infiltration of CCR2^+^ monocytes into injured muscle (Supplemental Figure 7D) and concomitantly increased circulating monocyte proportions (Supplemental Figure 7E), indicating impaired tissue recruitment. Moreover, CVC treatment resulted in significantly lower expression of GSDME and IL-18 in injured muscle, as shown by flow cytometry (Supplemental Figure 7F), and reduced IL-18 concentrations confirmed by ELISA (Supplemental Figure 7G). Importantly, blockade of CCR2^+^ monocyte recruitment prevented the expansion of the Lyve1^+^ TRM pool (Supplemental Figure 7H), accompanied by marked impairment in muscle functional recovery, as evidenced by reduced voluntary wheel running activity (Supplemental Figure 7I). Together, these data indicate that recruited CCR2^+^ monocyte–derived macrophages are functionally required for activation of the GSDME–IL-18 pathway, thereby playing a critical role in supporting effective muscle regeneration.

### GSDME deletion impairs TRM expansion dynamics.

**Figure 7:**
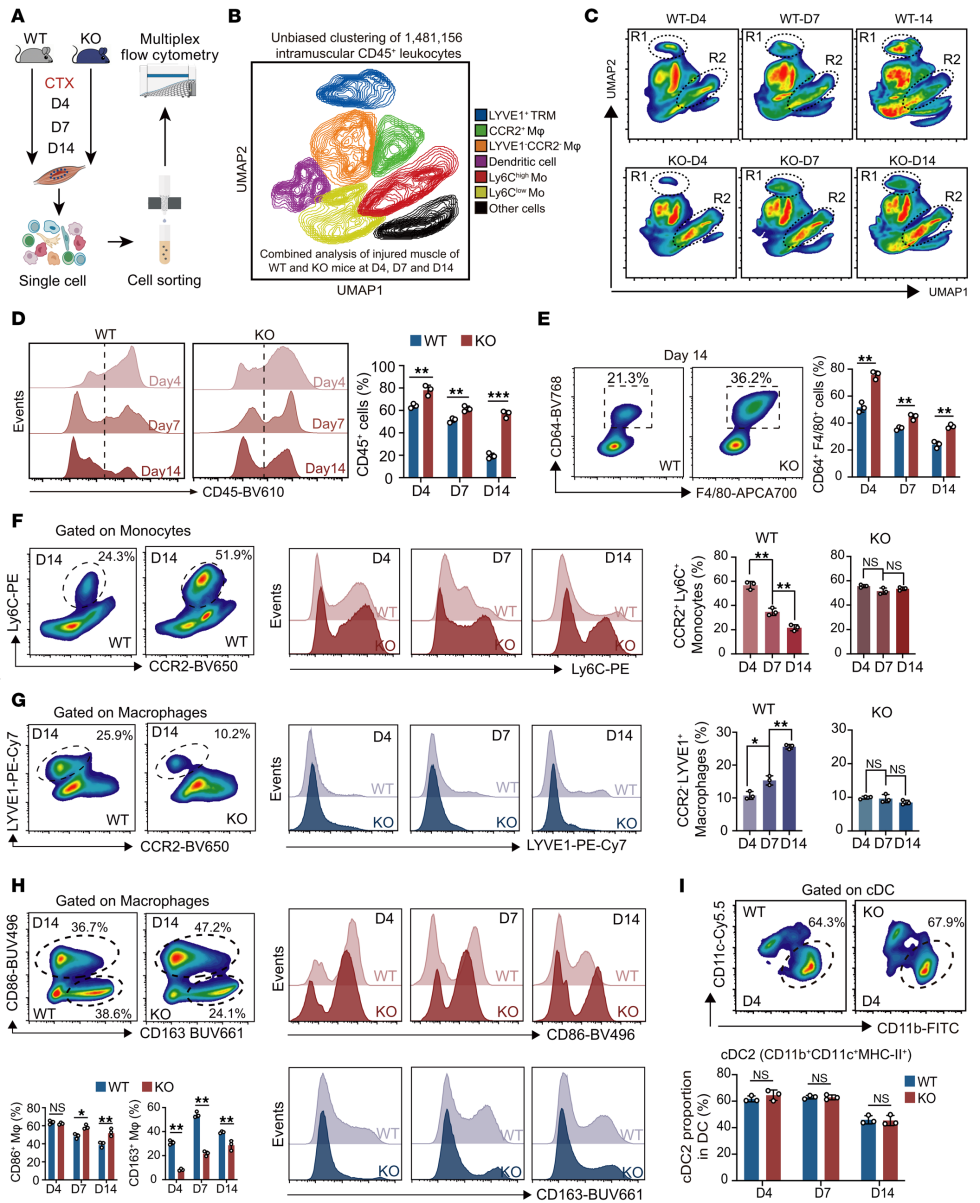
GSDME controls the recruitment and expansion of intramuscular immune cells, especially TRMs. (**A**) Experimental design for flow cytometry analysis of injured gastrocnemius muscles from WT and KO mice at D4, D7, and D14. Muscle tissues were digested into single-cell suspensions, and CD45^+^ cells were isolated using magnetic activated cell sorting. (**B**) High-dimensional analysis of multiplexed flow cytometry using an unbiased nonlinear dimensionality reduction algorithm to identify the immune cell populations in injured skeletal muscle of WT and KO mice. A total of 1,481,156 CD45^+^ leukocytes from injured muscle of WT and KO mice at D4, D7, and D14 after injury were analyzed. The mice at different days after injury were arranged to undergo euthanasia simultaneously to avoid batch effect. Full spectral panels and key antibodies are shown in Supplemental Figure 8A. (**C**) A UMAP visualization of dynamic changes in intramuscular immune cell populations between WT and KO mice at D4, D7, and D14 after injury. The regions with obvious differences between WT and KO mice are indicated with hollow elliptical shapes. (**D**) Time-series analyses of CD45^+^ leukocytes within the total cell population of injured skeletal muscle using flow cytometry with manual gating. (**E**) Time-series analyses of F4/80^+^CD64^+^ macrophages within the CD45^+^ leukocytes of injured skeletal muscle. (**F** and **G**) Time-series analyses of proportions of CCR2^+^Ly6C^+^ monocytes (**F**) and CCR2^–^LYVE1^+^ macrophages (**G**) within the CD11b^+^ myeloid of injured skeletal muscle. (**H**) Time-series analyses of proportions of CD86^+^ and CD163^+^ cells within the macrophages of injured skeletal muscle tissue. (**I**) The proportion of cDC2 within total DC (CD11c^+^MHC-II^+^) population of injured skeletal muscle. Data are presented as mean ± SEM. For **D**, **E**, **H**, and **I**, by unpaired 2-tailed Student’s *t* test; for **F** and **G**, by 2-way ANOVA with Tukey’s post hoc test. *P < 0.05, **P < 0.01, ***P < 0.001.

To scrutinize the characteristics of immune cells at the protein level in the injured muscle, we collected 1,481,156 CD45^+^ leukocytes from injured skeletal muscle of WT and KO mice at D4, D7, and D14 and further analyzed them using multiplex flow cytometry with antibodies against F4/80, CD64, CD11b, Ly6C, LYVE1, and CCR2, as well as using the nonlinear dimensionality reduction algorithm (Figure 7A and Supplemental Figure 8A). Using UMAP-guided unbiased gating, they were clustered into 6 groups (LYVE1^+^ TRMs, CCR2^+^ macrophages, LYVE1^–^CCR2^–^ macrophages, DCs, Ly6C^+^ monocytes, and Ly6C^–^ monocytes) (Figure 7B) as described in our recent work (20). Multi–time point analysis (D4, D7, and D14) revealed the dynamic evolution of the immune landscape and the impact of GSDME deficiency after injury (Figure 7C). In WT mice, TRMs exhibited a progressive expansion from D4 to D14, which was markedly suppressed in KO mice at D7–D14 (region 1). Moreover, the proportion of Ly6C^hi^ monocytes in KO mice was consistently higher than that in WT mice across all 3 postinjury time points (region 2) (Figure 7C). Together, these results suggest that the disrupted expansion of TRMs, accompanied by persistent Ly6C^hi^ monocyte accumulation, reflects a failure of inflammatory resolution in KO mice after injury.

We further evaluated the changes in immune cells using manual gating (gating strategy in Supplemental Figure 8B). It was shown that CD45^+^ leukocytes infiltration peaked at D4 and subsided by D14 in WT mice. In contrast, KO mice exhibited a failure to resolve inflammation, resulting in sustained leukocytes infiltration at D14 (Figure 7D). The number of CD64^+^F4/80^+^ macrophages was consistently higher in GSDME-KO mice than in WT mice across D4, D7, and D14 after injury (Figure 7E). Based on the flow cytometry gating strategy, subsequent analysis of Ly6C expression within the monocyte population and LYVE1 staining within the macrophage subset revealed that the decline of CCR2^+^Ly6C^+^ monocytes (Figure 7F) and the increase of CCR2^–^LYVE1^+^ macrophages (Figure 7G) were significantly inhibited in KO mice. Notably, this defective expansion of LYVE1^+^ TRMs persisted through the late regeneration phase and remained significantly impaired in KO mice even at D21 after injury (Supplemental Figure 8, C and D). Macrophages in GSDME-KO mice were skewed toward a proinflammatory phenotype, as evidenced by sustained high CD86 and low CD163 expression from D4 to D14 (Figure 7H). Meanwhile, cDC2 numbers remained unchanged across genotypes (Figure 7I), suggesting that the impaired regeneration is unlikely due to DC–mediated antigen presentation disturbances. Taken together, these findings suggest that GSDME deficiency disrupts the expansion of LYVE1^+^ TRMs by impairing the inflammatory niche shaped by recruited macrophages during muscle regeneration.

### GSDME–IL-18 axis activates KLF4/JUN to maintain TRMs.

**Figure 8:**
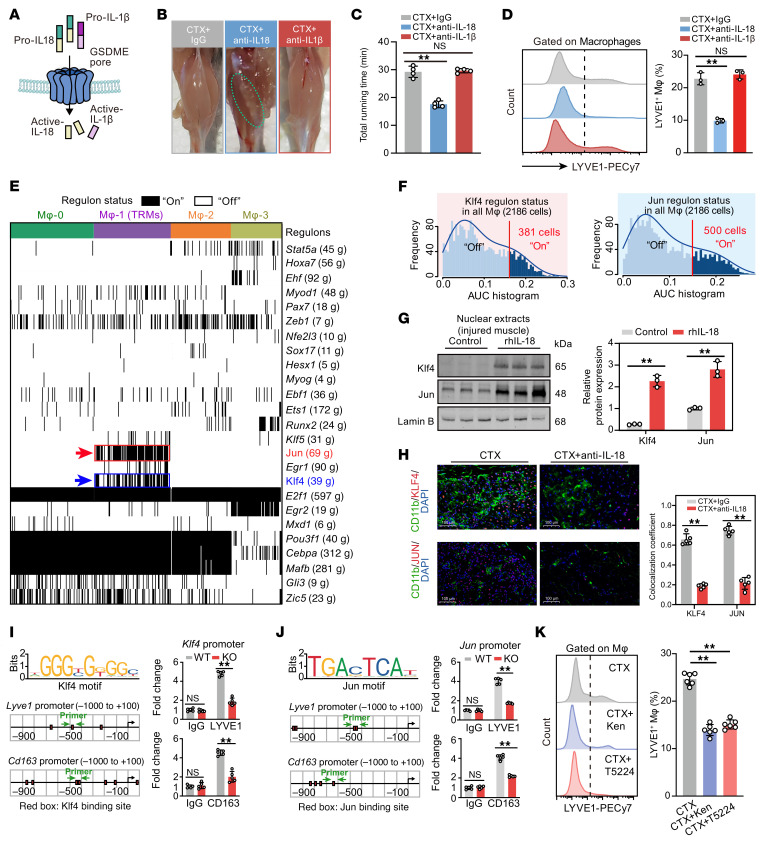
Regulation of GSDME on TRMs relies on IL-18–induced transcriptional regulators KLF4 and JUN. (**A**) Schematic model of GSDME-mediated pyroptosis. (**B**) Representative images of CTX-injured gastrocnemius muscle treated with neutralizing antibodies against IL-1β, IL-18, or control IgG. (**C**) Exercise performance of CTX-injured mice treated with anti–IL-1β, anti–IL-18, or IgG. (**D**) Expression levels of LYVE1^+^ macrophages in injured muscle were determined by flow cytometry. (**E**) SCENIC analysis of TF regulons in 4 macrophage subclusters, showing on/off status and number of target genes. Klf4 and Jun regulons are selectively activated in TRMs (Mφ-1). (**F**) The numbers of macrophages with “on” status of Klf4 and Jun regulons in all macrophages. (**G**) Nuclear protein levels of KLF4 and JUN in injured muscle from CTX+IgG and CTX+IL-18 mice. (**H**) Immunofluorescence of KLF4 and JUN in injured muscle sections from CTX+IgG or CTX+anti–IL-18 mice; colocalization with DAPI^+^ nuclei was quantified. Scale bars: 100 �m. (**I** and **J**) The binding of KLF4 and JUN to the promoters of *Lyve1* and *Cd163* was confirmed by ChIP-qPCR in injured muscle tissue from WT and KO mice. (**K**) The proportions of LYVE1^+^ macrophages in total CD11b^+^ myeloids isolated from the muscle of CTX-injured mice treated with the KLF4 inhibitor kenpaullone or the JUN inhibitor T5224. Data are presented as mean ± SEM. For **C**, **D**, and **K**, by 1-way ANOVA with Tukey’s post hoc test; for **I** and **J**, by 2-way ANOVA with Tukey’s post hoc test; for **G** and **H**, by unpaired 2-tailed Student’s *t* test. **P < 0.01.

Given the established role of GSDME-mediated pyroptosis in releasing IL-1β and IL-18 (Figure 8A), we sought to determine whether these cytokines contribute to muscle rehabilitation. Neutralizing IL-18, but not IL-1β, induced fatty replacement in the injured muscle (Figure 8B), as confirmed by gastrocnemius weight (Supplemental Figure 9A) and running performance test (Figure 8C and Supplemental Figure 9B). Blocking IL-18 potently inhibited the expansion of TRMs after injury (Figure 8D). CSA analysis, SDH staining, and fiber ratio experiments (Supplemental Figure 9, C and E) showed similar results. Thus, IL-18, but not IL-1β, drives muscle repair via TRM regulation.

To identify TFs for TRMs maintenance, we used Single-Cell Regulatory Network Inference and Clustering (SCENIC) (36) and found KLF4 and JUN to be 2 key TFs in *Lyve1*^+^*Cd163*^+^*Txnip*^+^ Mφ-1 (Figure 8E). At the transcriptional level, *Klf4* and *Jun* were primarily coexpressed in Mφ-1 (Supplemental Figure 9F). According to the SCENIC analysis of regulons within Mφ-1, there were 33 target genes specific to KLF4 and 68 target genes specific to JUN (Figure 8E). Additionally, 25 target genes overlapped between KLF4 and JUN targets (Supplemental Figure 9G). Among these, we found *Lyve1, Folr2*, and *Cd163*, 3 core marker genes for TRMs across organs (28, 29). Furthermore, based on the expression patterns observed in the UMAP visualization of macrophages in our scRNA-seq data, *Txnip*, *Ier2*, *Fcna*, *Cbx4*, *Fos*, *Egr1*, *Klf2*, *Irf1*, and *C4b* were predominantly expressed in Mφ-1, indicating they may serve as potential regulators of skeletal muscle TRMs (Supplemental Figure 9H). AUCell (36) was used to assess the activity of these regulons at the single-cell level, showing that approximately 17% and approximately 23% of macrophages displayed “on” status in KLF4 and JUN activity (Figure 8F). In support of these findings, IL-18 treatment increased nuclear KLF4 and JUN protein levels in macrophages isolated from injured muscle by FACS (Figure 8G), while neutralizing IL-18 reduced their levels (Supplemental Figure 9I). Immunofluorescence showed that neutralizing IL-18 inhibited nuclear KLF4 and JUN expression in injured muscle (Figure 8H). ChIP-qPCR confirmed KLF4 and JUN binding to the *Lyve1* and *Cd163* promoter area (5′ flanking regions from –1,000 to +100), which was reduced in KO mice (Figure 8, I and J). KLF4 inhibitor kenpaullone and JUN inhibitor T5224 reduced LYVE1^+^ cells in injured muscle (Figure 8K). These results highlight KLF4 and JUN as 2 crucial drivers of TRMs during muscle repair in response to IL-18.

### IL-18 treatment restores regeneration in aged muscle with impaired GSDME signaling.

Aging is associated with impaired skeletal muscle regeneration. To investigate whether the GSDME–IL-18 axis contributes to this defect, we compared muscle tissues from young (<45 years) and aged (>70 years) patients undergoing abdominal surgery. *GSDME* mRNA levels were significantly reduced in aged human muscle (Supplemental Figure 10, A and B). A similar decline was observed in mice, with gastrocnemius muscle from 24-month-old mice showing markedly lower *Gsdme* mRNA levels than 8-week-old counterparts (Supplemental Figure 10C). Furthermore, the cleaved (active) forms of GSDME and IL-18 were readily detected in injured muscles of young mice but were diminished in old mice (Supplemental Figure 10D), suggesting age-dependent impairment of this axis.

**Figure 9:**
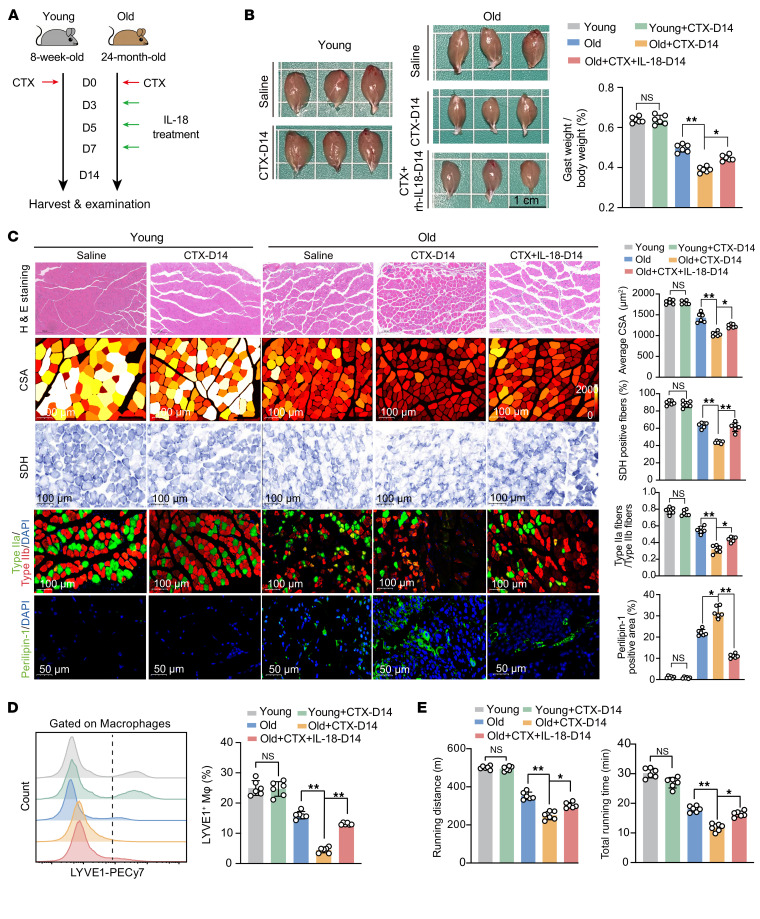
Administration of IL-18 rectifies aging-associated defective skeletal muscle repair. (**A**) Experimental design of CTX-induced muscle injury in young and aged mice with IL-18 administration to aged mice at 3 time points (**B**) Representative morphology and quantitative analysis of the gastrocnemius (Gast) weight/body weight ratio in repaired skeletal muscle from 5 groups of mice: young, young+CTX-D14, old, old+CTX-D14, and old+CTX-D14 treated with recombinant IL-18 (old+CTX+IL-18-D14). At D14, the injured skeletal muscle typically undergoes recovery. Scale bar: 1 cm. (**C**) Representative images and quantitative analyses of H&E staining and CSA analysis (muscle fiber structure), SDH staining (mitochondrial function and muscle fiber typing), MyHC fiber type-IIa/type-IIb immunofluorescence staining (muscle fiber typing), and perilipin-1 immunofluorescence staining (fatty accumulation) in injured skeletal muscle from 5 groups of mice: young, young+CTX-D14, old, old+CTX-D14, and old+CTX+IL-18-D14. Scale bars: 100 �m, 50 �m (bottom row). (**D**) Representative images and quantitative analyses of flow cytometry for LYVE1^+^ macrophages within total CD11b^+^ myeloids in repaired skeletal muscle from 5 groups of mice: young, young+CTX-D14, old, old+CTX-D14, and old+CTX+IL-18-D14. (**E**) The exercise performance of 5 groups of mice: young, young+CTX-D14, old, old+CTX-D14, and old+CTX+IL-18-D14. Data are presented as mean ± SEM. One-way ANOVA with Tukey’s post hoc test was performed. *P < 0.05, **P < 0.01.

To test its functional relevance, we induced muscle injury using CTX in young and aged mice and administered recombinant IL-18 to old mice (Figure 9A). Compared with young mice, aged mice displayed impaired regeneration at D14 after injury, which was partially rescued by IL-18 treatment (Figure 9, B and C). IL-18 restored muscle fiber structure, mitochondrial activity, and reduced fatty infiltration, as shown by H&E, SDH, MyHC, and perilipin-1 staining. IL-18 also restored the proportion of LYVE1^+^ TRMs (Figure 9D) and improved exercise performance (Figure 9E). These findings indicate that IL-18 treatment rejuvenates impaired muscle repair in aged mice.

## Discussion

In this study, we provide converging evidence that refines current understanding of how pyroptosis contributes to muscle regeneration. First, our data support a role of GSDME-driven inflammatory signaling in preventing maladaptive myosteatosis and supporting tissue restoration, suggesting a functional dimension beyond its canonical execution of cell death. Second, by integrating scRNA-seq and fate-mapping analyses, we show that GSDME-dependent pyroptotic signaling is functionally linked to recruited CCR2^+^ monocyte–derived macrophages and is coupled to the expansion of TRMs, thereby shaping the metabolic niche of injured muscle. Third, we delineate a GSDME–IL-18–KLF4/JUN regulatory axis that orchestrates TRM maintenance during regeneration. Finally, we reveal that attenuated IL-18 signaling underlies age-associated defects in TRM recovery and that supplementation of IL-18 can partially restore regenerative capacity in aged muscle. Together, these findings highlight GSDME-mediated pyroptotic signaling as a previously underappreciated regulatory layer in skeletal muscle healing.

Inflammation is the most important biological process in both muscle injury and rehabilitation; thus, understanding the inflammatory processes in greater detail may provide new precise therapeutic strategies. Although pyroptosis has been proven to be a conserved immune surveillance and defense mechanism for clearance of pathogens in infection (37, 38), its actions in noninfectious conditions are not fully understood. Our results were extremely unexpected since a large number of studies have showed the harmful action of highly proinflammatory pyroptosis in organ damage (13–15). It has been noted that while the upstream cleaver of GSDME caspase-3 is reported to be required for skeletal muscle differentiation and myogenesis (39, 40), the underlying mechanism remains unclear. It should be mentioned that another gasdermin, GSDMD, may function as a metabolism modulator during skeletal muscle repair (16, 17). Mehrotra et al. showed that GSDMD activates macrophages to adopt a subpyroptotic state without releasing IL-1β or IL-18, instead secreting a secretome containing prostaglandin E2, which promotes tissue repair properties (16). Additionally, Chi et al. elaborated that macrophages in injured muscle may bypass pyroptosis and transition into a hyperactive state, characterized by pore formation mediated by GSDMD, enabling the release of 11,12-epoxyeicosatrienoic acid to promote tissue repair without the release of IL-1β and IL-18 (17). These 2 investigations provide strong evidence that GSDMD functions as a nonimmune/inflammatory regulator in tissue repair. Thus, our study establishes GSDME as the dominant regulator in muscle repair, suggesting functional divergence among the gasdermins.

Skeletal muscle typically resolves inflammation and regenerates efficiently, but aging or chronic injury can drive FAPs toward adipogenesis, leading to pathological myosteatosis. In WT muscle, cluster 6 genes associated with anabolic metabolism, and myogenic progression steadily increased from D4 to D7, consistent with the peak phase of repair. In contrast, KO muscle showed a transient D4 elevation of cluster 6–associated transcripts but did not sustain regenerative transcriptional programs at D7, aligning with persistent inflammation and delayed resolution. By D14, lipid-remodeling signatures predominated in KO muscle, consistent with a progressive shift toward pathological remodeling. Consistent with this shift, unbiased lipidomic profiling revealed marked accumulation of multiple lipid classes, including TAG, PC, SHexCer, and complex sphingolipids, in GSDME-deficient muscle. Together, these observations indicate that the loss of GSDME weakens the normal transition from inflammatory resolution to regeneration, leaving injured muscle susceptible to maladaptive adipogenic remodeling and associated metabolic stress during the late healing phase. Rather than acting solely as a pore-forming effector of pyroptosis, GSDME appears to safeguard the fidelity and persistence of regenerative programs, thereby preventing excessive FAP-derived lipid accumulation. This functional expansion highlights an unanticipated role of GSDME in maintaining tissue metabolic homeostasis during skeletal muscle repair.

To understand why lipid accumulation increases under GSDME deficiency, we performed tissue-specific rescue experiments. Restoring GSDME in myeloid cells, but not in FAPs, normalized muscle repair and metabolic status, indicating that macrophage-intrinsic GSDME is essential. Transcriptional alterations observed in MuSCs, such as impaired OXPHOS, therefore likely reflect secondary consequences of disrupted immune–stromal crosstalk. Single-cell and flow cytometry profiling further revealed selective impairment in the expansion of intramuscular Lyve1^+^Cd163^+^Txnip^+^ TRMs in KO mice. These TRMs, originally fetal derived and tissue specialized (41), showed transcriptional features consistent with phagocytic and metabolic regulation, suggesting their role in maintaining an energetically supportive niche (42–44). However, skeletal muscle TRMs remain less well characterized compared with those in other organs (45). Recently, LYVE1, CD163, and TXNIP have been increasingly recognized as conserved markers of TRMs, including those in skeletal muscle (28–31). Emerging evidence indicates that macrophage–FAP interactions critically shape fibro-adipogenic remodeling (46–49), and our findings provide in vivo support for this evolving concept by demonstrating that compromised TRM integrity predisposes muscle to pathological adipogenesis. Mechanistically, we identified KLF4 and JUN as transcriptional regulators of TRMs expansion in vivo, with downstream targets including Lyve1, Folr2, and Cd163, consistent with prior studies (50, 51). Nevertheless, elucidating the signaling mechanisms by which TRMs restrain FAP adipogenesis represents an important direction for future investigation. While this will not fully resolve the multifaceted interplay between macrophages and FAPs during muscle repair, our findings provide a plausible mechanistic entry point to begin unraveling this complexity.

Interestingly, we identified IL-18 as a cytokine that promotes skeletal muscle repair by activating KLF4 and JUN transcriptional activity. KLF4 has been implicated in promoting myofiber repair and limiting fibrosis following injury, in part through remodeling the muscle regenerative niche (52, 53). Muscle-specific deletion of KLF4 has been shown to impair regeneration by regulating myoblast proliferation via p57 and promoting myocyte fusion via Myomixer (54). Likewise, JUN has been reported to antagonize MyoD transcriptional activity (55), and genomic analyses further demonstrated that JUN cooperates with MyoD to control muscle-specific enhancers (56). Together, these studies establish KLF4 and JUN as essential regulators of muscle repair. Consistent with these findings, our results suggest that GSDME–IL-18 signaling functions upstream to support the activation of these regenerative transcriptional programs in vivo. While IL-18 is a well-known inflammasome-related cytokine linked to inflammatory diseases, it also exhibits metabolic functions, including AMPK activation and enhanced fat oxidation in skeletal muscle (57). In line with these metabolic effects, IL-18 deficiency leads to lipid accumulation and obesity (58), highlighting its context-dependent pleiotropic activities, likely governed by tissue microenvironmental cues. Here, we show that IL-18 restrains myosteatosis during the muscle-healing process, potentially by sustaining mitochondrial OXPHOS in TRMs. Notably, our findings reveal that the GSDME–IL-18 axis is markedly impaired in aged humans and mice, suggesting an age-associated deficiency in pyroptosis-mediated regenerative signaling in skeletal muscle. Given IL-18’s established safety profile in clinical trials for cancer immunotherapy (59), these results underscore its translational potential for age-related myosteatosis, muscle atrophy, and sarcopenia. At the same time, we recognize that IL-18–dependent activation of KLF4 and JUN is unlikely to explain all aspects of macrophage–FAP communication during regeneration. Clarifying how these transcriptional programs contribute to maintaining the niche and limiting adipogenesis will require further in vivo perturbation studies, such as selective modulation of KLF4 or JUN in TRMs. These investigations fall beyond the present scope but will be an important direction to pursue.

In summary, our findings reveal that the GSDME–IL-18 axis orchestrates GSDME-dependent pyroptotic signaling in the context of recruited CCR2^+^ monocyte–derived macrophages to establish a proregenerative immune niche, sustaining TRM expansion, restraining FAP adipogenesis, and promoting muscle regeneration. This axis may provide a conceptual basis for future therapeutic strategies targeting myosteatosis, muscle atrophy, and age-related sarcopenia.

## Methods

### Sex as a biological variable.

Human muscle samples included both male and female patients; however, sex was not used as a stratification variable in the analysis. All mechanistic animal experiments were performed in male mice to reduce variability associated with sex hormone fluctuations and to maintain consistency in skeletal muscle injury models. While the human data suggest general relevance across sexes, this study was not designed to systematically evaluate sex-specific differences.

### Animals.

WT C57BL/6J mice were purchased from Sino-British SIPPR/BK Lab Animal Ltd. *Gsdme*-KO mice and *Gsdme*^S/S^ mice were described in our previous study (18, 20). *Lysm*-Cre mice [B6.129P2-*Lyz2*tm1(cre)Ifo/J, #004781] and *Pdgfra*-Cre mice [C57BL/6-Tg(*Pdgfra*-cre)1Clc/J, #013148] mice were obtained from The Jackson Laboratory. *Gsdme^S/S^ Lysm*-Cre and *Gsdme*^S/S^ *Pdgfra*-Cre mice were generated by crossbreeding Gsdme^S/S^ mice with *Lysm*-Cre mice or *Pdgfra*-Cre mice, respectively. Heterozygotes were crossed to each other to produce littermates either homozygous for the KO allele or the WT allele. Only male mice were used in the present study. All mice were kept under specific pathogen–free conditions and were housed in a temperature-controlled (23°C ± 2°C) and humidity-controlled (40% ± 5%) environment with access to water and diet, under a 12 h light/12 h dark cycle.

### Human skeletal muscle samples.

Thirty patients with abdominal surgery were enrolled between 2022 and 2024 at the Eastern Hepatobiliary Surgery Hospital affiliated with Naval Medical University. During abdominal surgery, a small amount of rectus abdominis (<0.5 g) was isolated and stored at –80°C.

### Mouse model and treatments.

For CTX-induced muscle injury, mice were anesthetized with i.p. injection of ketamine-xylazine in combination (10 mg/kg:2 mg/kg) and subsequently were injected with 50 μL of 10 μM CTX (*Naja pallida*, Latoxan Laboratory, L8102-1MG) in gastrocnemius muscle. In the Sham group, the same volume of saline was injected. Injured tissues were collected at D4, D7, and D14 time points for biochemical analysis. For pharmacologic blockade of CCR2 signaling, mice received daily injections of the CCR2 antagonist CVC (Biorbyt, orb402001, 20 mg/kg, i.p.) for 14 days as described previously (35). Vehicle controls received matched solvent alone. For in vivo cytokine injections, IL-18 (R&D Systems, catalog 9124-IL, 1 μg per injection) was administered i.p. individually. For neutralization of IL-1β or IL-18, anti–IL-18 antibody (Bio X Cell, BE0237, 200 μg) and anti–IL-1β antibody (Bio X Cell, BE0246, 200 μg) were administered i.m. coincident with CTX injection. To inhibit KLF4 and c-Jun signaling pathways, Kenpaullone (MedChemExpress, HY-12302, 10 mg/kg) and T-5224 (MedChemExpress, HY-12270, 3 mg/kg) were administered i.m. at the injury site concurrently with CTX injection and every other day thereafter. Vehicle controls matched solvent compositions. The same quantity of matched IgG was used as control.

### Voluntary wheel running activity.

Mice were individually housed in a cage with a running wheel as described previously (60). Mice were individually run in 8 separate cohorts at a time. No formal acclimation period was provided. At D14 after injury, running wheel activity was recorded twice. Data were collected on total distance, average speed, and the max speed.

### Grip strength test.

A grip strength meter (Bioseb, model BIO-GS3) was used to measure the hind limb grip strength of the mice. The strength meter was reset to 0 g after stabilization in each test. The metronome was used to give a beat of 3 s (3 s is sufficient to prepare for the next test round and compress the recovery time of the muscle). The mouse’s tail was pulled back at a constant speed after it grasped the grid during a measurement within 3 s. The peak force in grams was recorded at the time the mouse released its paws from the grid in each measurement. Five minutes later, the recording was repeated. The test was performed twice in 2 days, and the average numerical value was used. The order of mice tested on each day was randomized. All the tests were operated by 1 person to ensure reliability.

### Transmission electron microscopy.

Transmission electron microscopy was performed as described previously (61). Tissues were fixed with 2% paraformaldehyde and 2% glutaraldehyde in 0.1 mol/L phosphate buffer (pH 7.4), followed by postfixation for 8 h in 1.5% osmium tetraoxide. After dehydration with graded alcohols, samples were dehydrated in a graded ethanol series and embedded in epoxy resin. Samples were sectioned (80 nm), counterstained with uranylacetate and lead citrate, and observed with a transmission electron microscope (Hitachi H-800). Images were acquired digitally from a randomly selected area, and the number of membrane pore formations was calculated.

### Histology and IHC.

Muscle tissues were fixed in 4% PFA and paraffin embedded. Sections (8 μm) were stained with H&E, Oil Red O, or SDH using standard protocols. For IHC, sections underwent antigen retrieval in sodium citrate buffer. After blocking endogenous peroxidase and nonspecific binding, primary antibodies were applied for 4 h, followed by incubation with biotinylated secondary antibodies and HRP streptavidin. Signal was developed with DAB and counterstained with hematoxylin. All slides were imaged using an Olympus BX51 microscope. The antibodies used in IHC are listed in Supplemental Table 2.

### Tyramide signal amplification–based multiplex IHC analysis.

Muscle slices were treated in 0.03% H_2_O_2_ for 15 min to quench endogenous peroxidase activity, permeabilized, and blocked with 0.3% Triton X-100/5% BSA in PBS. Then, the slices were incubated with the first primary antibody followed by incubating with corresponding second antibody. After being washed thoroughly, signal amplification was achieved using specific tyramide signal amplification dye and terminated using PBS. Then, the slices were subjected to eluent solution to remove unbound antibodies and reblocked. Next, the slices were stained with the second primary antibody, followed by restaining with other antibodies repeatedly. After several rounds of staining, antifluorescence quenching sealing tablets were added with DAPI for 10 min, and imaging was performed using a Pannoramic MIDI (3DHISTECH Ltd.). The quantitative analysis was performed by HALO (Indica Labs). Detailed dilution and incubation times are provided in the figure legends.

### Seahorse metabolic analysis.

OCR and extracellular acidification rate (ECAR) were measured using an XFe24 Extracellular Flux Analyzer (Seahorse Bioscience) as described in our previous study (62). Extracts from muscle samples were seeded on a Seahorse 24-well analyzer and tested using XFe 24 assay with the Seahorse XF Real-Time ATP Rate Assay Kit (Agilent). OCR was measured in basal conditions and in response to 2 μM oligomycin (ATP synthase inhibitor), 2 μM rotenone (Complex I inhibitor) and 2 μM antimycin A (Complex III inhibitor). ECAR was measured during the entire experiment. The proportions of ATP contents produced by OXPHOS or glycolysis were calculated. Analysis was performed using Seahorse Wave Desktop Software (Agilent).

### O2K metabolic analysis.

The assessment of mitochondrial respiration in myofibers isolated from control mice and CTX injury mice was performed using an Oroboros O2K high-resolution fluororespirometer (Oroboros Instruments; data were recorded with Data Lab 7.4 software from Oroboros Instruments) at 37°C in 2 mL of buffer. Five milligrams (wet weight) of myofibers was weighed and added to the respiration chamber. The following substrates were added sequentially: 10 mM glutamate, 5 mM malate (G + M), 2 mM ADP, 10 mM succinate, and 400 μM antimycin A. Respiration rates were normalized as nanomoles of dioxygen per minute per milligram of wet muscle mass. The oxygen consumption was analyzed with the software (Oroboros Instruments).

### Deep learning–based muscle segmentation and quantification.

We calculated the average CSA of myofiber using a customized workflow. Brieﬂy, the muscle sections were stained with H&E to visualize myofibers. First, the whole cross section was detected through a Gaussian-blurred version of the laminin signal (Σ = 5 μm), followed by an absolute threshold (threshold value: 5). Then, these images were processed through the Cellpose deep-learning algorithm (63), a general tool used to automatically segment individual fibers from extracellular matrix.

### Magnetic activated cell sorting.

Magnetic activated cell sorting was performed according to the manufacturer’s instructions (Miltenyi Biotec). Brieﬂy, injured muscles were harvested and mechanically and enzymatically digested with 3 mL sCelLive tissue dissociation solution (Singleron) in a Singleron PythoN tissue dissociation system at 37°C for 30 min. Cells were isolated using the CD11b MicroBead Kit (Miltenyi Biotec, 130-097-142) or PDGFRa MicroBead Kit (Miltenyi Biotec, 130-101-502).

### FACS.

Single-cell suspensions were prepared as described above. Cells were washed twice with HBSS and then stained with Zombie Violet viability dye (BioLegend) for 15 min at room temperature in the dark. Subsequently, cells were incubated for 30 min at 4°C with a cocktail of fluorochrome-conjugated antibodies diluted in Brilliant Stain Buffer (BD Biosciences). The antibody panel included anti-CD45, anti-CD11b, anti-F4/80, and anti-CCR2. Following staining, cells were washed and resuspended in ice-cold sorting buffer. Macrophages were isolated as live (Zombie Violet^–^) CD45^+^CD11b^+^F4/80^+^ cells, while CCR2^+^ recruited macrophages were isolated as live CD45^+^CD11b^+^F4/80^+^CCR2^+^ cells using a BD FACSAria III cell sorter (BD Biosciences).

### Multiplex flow cytometry and high-dimensional analysis.

Single-cell suspension was preincubated with 0.025 μg of TruStain FcX anti-CD16/32 antibody (BioLegend) for 10 min on ice to block Fc receptors. Then, the cells were stained with appropriate antibodies at 4°C in the dark for 45 min. Dead cells were excluded using LIVE/Dead (Thermo Fisher Scientific L34981) staining. The cells were clustered using multiplexing flow cytometry with antibodies (1:50 dilution), as shown in Supplemental Figure 7A, for 30 min at 4°C. When assessing intracellular markers, cells were first washed in PBS and then incubated with LIVE/Dead for 45 min at 4°C, protected from light before following the approach detailed above. After the final wash step, cells were subsequently fixed and permeabilized using the Transcription Factor Staining Buffer Set (eBioscience), and intracellular staining was performed using antibodies detailed in Supplemental Table 2. Samples were acquired using a CytoFLEX LX (Beckman) and subjected to high-dimensional analysis using an unbiased nonlinear dimensionality reduction algorithm (UMAP) in FlowJo software (version 10; BD Biosciences). The quality control of events was managed using FlowAI (version 2.3.2; BD Bioscience). The manual gating strategy is presented in Supplemental Figure 7B. FACS was performed on a BD FACSAria III system controlled by FACSDiva software (v8.0.2).

### Immunoblotting.

Skeletal muscle tissue and cultured cells were lysed in RIPA buffer containing protease/phosphatase inhibitors. Protein extracts were separated on 8%–12% SDS-PAGE gels and transferred to nitrocellulose membranes. After blocking with 5% nonfat milk, membranes were incubated overnight at 4°C with primary antibodies. Following washes, membranes were incubated with IRDye 800CW-conjugated secondary antibodies. Blots were imaged using an Odyssey system and analyzed with ImageJ. The catalog numbers of antibodies are listed in the Supplemental Table 2.

### qPCR.

RNA was extracted using TRIzol reagent, and its quality was verified by a NanoDrop spectrophotomer (Thermo Fischer Scientific) at a 260/280 nm ratio (1.8–2.0). Following reverse transcription with a gDNA removal kit, qPCR was performed on a Bio-Rad CFX96 system using SYBR Green. GAPDH served as the internal control, and all samples were run in duplicate. Primer sequences are listed in Supplemental Table 2. Ct values were recorded, and the **^ΔΔ^**CT method was used to quantify fold changes of genes.

### ChIP-qPCR.

ChIP was performed based on a previously published protocol (64). Single cells isolated from injured muscle were cross-linked with DTBP/DSP (3.3 and 1 mg/mL, respectively) for 15 min, followed by 4% paraformaldehyde fixation for another 15 min at room temperature. After quenching with glycine, nuclei were isolated, lysed, and sonicated using a microtip probe (30% power, 10 cycles of 30 s on/45 s off). Chromatin was precleared with IgG and Protein A/G beads, and equal aliquots were incubated overnight at 4°C with 10 μg of anti-LYVE1, anti-CD163, or control rabbit IgG. Immunoprecipitated DNA was eluted and purified by phenol/chloroform extraction. Promoter regions of *LYVE1* and *CD163* were amplified by qPCR (SYBR Green) using specific primers (Supplemental Table 2). Fold enrichment relative to IgG control was calculated from input-standardized Ct values and normalized to *GAPDH*. The results are expressed as the fold change in pull-down samples relative to the IgG control.

### RNA-seq.

Two batches of RNA-seq were performed. In the first batch, the total RNA was extracted from the injured gastrocnemius muscle and the contralateral non-injury muscle (*n* = 3 per group) at 24 h after injury. In the second batch, the total RNA was isolated from the injured gastrocnemius of injured WT or KO mice at 4 different time points (D1, D4, D7, and D14, *n* = 3 per group at every time point) using TRIzol reagent according the manufacturer’s instructions (Invitrogen), and genomic DNA was removed using DNase I (TaKara). Then, RNA quality was determined with a 2100 Bioanalyzer (Agilent) and quantified using the ND-2000 spectrophotometer (NanoDrop Technologies). Libraries were size selected for cDNA target fragments of 300 bp on 2% low-range ultra agarose followed by PCR amplification using Phusion DNA polymerase (New England Biolabs) for 15 cycles. After quantification by the TBS-380 fluorometer (Turner BioSystems), the paired-end RNA-seq library was sequenced with the Illumina HiSeq 4000 sequencer.

### Lipidomic analysis.

The lipid analysis of injured muscle tissue from 10 mice (*n* = 5 per group) was performed using liquid chromatography–tandem mass spectrometry as described previously (65). Approximately 5 mg of tissue was homogenized in 200 μL water and 480 μL MTBE/MeOH (5:1, with internal standard). After vortexing, samples underwent 3 cycles of homogenization (35 Hz, 4 min) and sonication (5 min, ice bath), followed by incubation at –40°C for 1 h and centrifugation (1,000*g* 15 min, 4°C). Supernatants were vacuum-dried, reconstituted, clarified by centrifugation, and subjected to liquid chromatography–mass spectrometry analysis with the following parameters: mobile phase A, 40% water/60% acetonitrile with 10 mM ammonium formate; mobile phase B, 10% acetonitrile/90% isopropanol with 10 mM ammonium formate; gradient, 40%→100% B (1.0–12.0 min), hold 100% B (12.0–13.5 min), reequilibrate to 40% B (13.5–18.0 min); column temperature, 55**°**C; autosampler, 4**°**C; and injection volume, 2 μL. Mass spectrometry data were acquired on a Q Exactive Orbitrap mass spectrometer (UPLC-QE-Orbitrap-MS, Thermo Fisher Scientific) in DDA mode (MS1, 70,000; MS2, 17,500; NCE 15/30/45). Raw files were converted to mzXML (ProteoWizard) and processed in XCMS (CentWave; minfrac, 0.5; cutoff, 0.3). Lipids were annotated by spectral matching against the LipidBlast database (https://fiehnlab.ucdavis.edu/projects/LipidBlast).

### scRNA-seq.

Single cells were prepared from the injured skeletal muscle tissue of WT and KO mice (*n* = 5 per group) as mentioned above and subjected to scRNA-seq (NovelBio Bio-Pharm Technology Co.) as described in our previous study (66). Cells were passed through a 40 μm cell strainer and enumerated by the Cellometer 2000 immediately before loading onto a 10x Chromium controller for cell capture (targeting 5,000 cells per sample) using the Chromium Single Cell 5′ Reagent Kit (v2 Chemistry Dual Index) (10x Genomics, catalog 1000263). Gene expression libraries were constructed following protocols from 10x Genomics. Quality control of the final libraries was performed using the Agilent TapeStation and then sequenced using Illumina’s Novaseq 6000, targeting 50,000 reads per cell.

### scRNA-seq data analysis.

Sequencing data were demultiplexed using CellRanger (v7.0.1) and processed with Seurat (v4.3.0) to obtain the UMI count matrix. After doublet removal (DoubletFinder, v2.0.4) and filtering of cells with >10% mitochondrial genes, 14,393 high-quality cells were retained. The top 2,000 highly variable genes were selected, and data were integrated across samples using Harmony (v1.2.0). Clustering at resolution of 0.8 identified 20 clusters, which were visualized via UMAP. Marker genes were identified with Seurat and annotated with SingleR (v2.0.0). Differential expression and enrichment analyses (KEGG and GO) were performed. Pathway activity was assessed with GSVA (v1.46.0), and cell–cell communication (particularly between SMCs and immune cells) was analyzed using CellChat (v1.6.1) (67). To validate the trajectory of cell subpopulation, monocle (v2.2.6) was used to infer pseudotime trajectories of selected subsets. To identify gene regulatory networks and infer transcription factor activity, SCENIC was used with the pySCENIC package (v0.12.1) in Python. To capture dynamic gene expression and predict cell state changes, RNA velocity analysis was performed using the scVelo package (v0.2.5) in Python. Loom files were generated from scRNA-seq data using Cell Ranger. These files, containing spliced and unspliced mRNA counts, were then used to estimate RNA velocities, allowing for the prediction of future cell states. The raw data of scRNA-seq are available in the Gene Expression Omnibus database under accession code GSE270614.

### Statistics.

All results are presented as mean ± SEM. The distribution of data within all individual groups was verified by the Shapiro-Wilk test of normality. For comparisons between 2 groups, statistical differences were assessed using an unpaired 2-tailed Student’s *t* test when data were normally distributed with equal variances, and Wilcoxon’s rank-sum test was applied for non-normally distributed data. For comparisons among more than 2 groups with equal variances, 1-way ANOVA (1 factor) or 2-way ANOVA (2 factors) was performed, followed by Tukey’s post hoc multiple-comparison test. Sample sizes were based on standard protocols in the field. The number of animals/samples in each group is indicated in the figure legends. A *P* value less than 0.05 was considered significant. All statistical analyses were performed using GraphPad Prism 8.

### Study approval.

All animal procedures were approved by the Institutional Animal Care and Use Committee of Naval Medical University and conformed to the NIH Guide for the Care and Use of Laboratory Animals (8th edition). Human studies were approved by the Research Ethics Committee of Eastern Hepatobiliary Surgery Hospital (EHBHKY2020-K-045), and written informed consent was obtained from all participants in accordance with the Declaration of Helsinki.

### Data availability.

Processed scRNA-seq data and bulk RNA-seq of this study were deposited in the NCBI Gene Expression Omnibus under accession codes GSE270614 and GSE271087. The lipidomics data have been deposited to MetaboLights repository with the study identifier MTBLS10509. Values for all data points are available in the Supporting Data Values file. All other data supporting the findings of this study are available as source data and from the corresponding author upon request.

## Author contributions

PW conceived and designed research. QC, JL, GH, SYW, GDL, YH, YTC, ZZ, JTF, and SJS performed experiments. XFC assisted with lipidomic analysis. CLZ, CQS, and FMS analyzed data. QC and PW wrote the manuscript. QC, DJL, and PW provided funding. All authors contributed with productive discussions and knowledge to the final version of this manuscript. Author order among co–first authors was determined by mutual agreement.

## Conflict of interest

The authors have declared that no conflict interest exists.

## Funding support

- National Natural Science Foundation of China (82373927 and 82073915 to PW, 82274030 and 82204537 to DJL).
- Shanghai Science and Technology Commission (LJ2024083 to PW).

## Supplementary Material

Supplemental data

Unedited blot and gel images

Supporting data values

## Version 1 02/17/2026

In-Press Preview

## Version 2 04/15/2026

Electronic publication
