# Supplementary material for: GSDME–IL-18 pyroptotic axis prevents myosteatosis by expanding tissue-resident macrophages to promote muscle regeneration
Source: J Clin Invest. 2026 Feb 17;136(8):e198076. doi: 10.1172/JCI198076 (PMC13078871; doi:10.1172/JCI198076)
Supplement: Supplemental data [file jci-136-198076-s277.pdf]

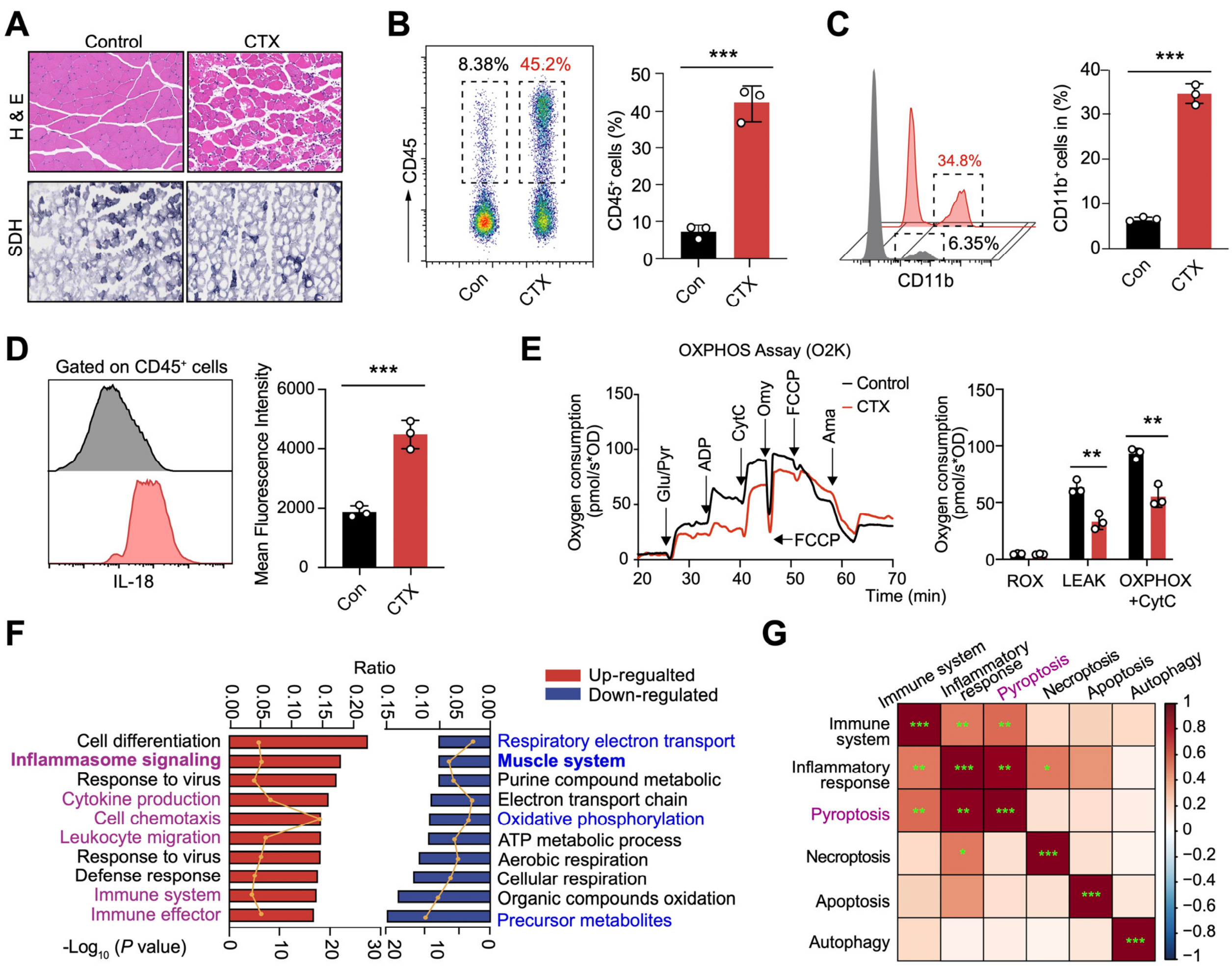

Supplemental Figure 1. Pyroptosis is activated in injured muscle.

(A) Representative images of HE staining and SDH staining in normal muscle and CTX-injured muscle at day 4 (D4) post-injury.

(B) Infiltration of $CD45^+$ leukocytes into injured muscle at D4 post-injury was evaluated using flow cytometry.

(C) Infiltration of $CD11b^+$ myeloid cells into injured muscle at D4 post-injury was evaluated using flow cytometry.

(D) A representative histogram and mean fluorescence intensity (MFI) quantification of IL18 expression from CTX-injured muscle of mice collected from Day 4.

(E) Evaluation of mitochondrial respiratory function in injured muscle at D4 post-injury using the O2K system. Glu/Pyr: glutamate/pyruvate; ADP: adenosine-5'-diphosphoric acid; CytC: cytochrome C; Omy: oligomycin; FCCP: carbonyl cyanide-4 (trifluoromethoxy) phenylhydrazone; Ama: antimycin A.

(F) Combined KEGG analysis showing upregulated and downregulated biological functions in muscle tissue from CTX-injected mice compared to control mice.

(G) Association between immune system activation and inflammatory response with several

types of regulated cell death (RCD), including pyroptosis, necroptosis, apoptosis, and autophagy.

Data are presented as mean ± SEM. Unpaired Student's t-tests between WT and KO were performed. *P<0.05, **P<0.01, ***P<0.001 KO vs WT.

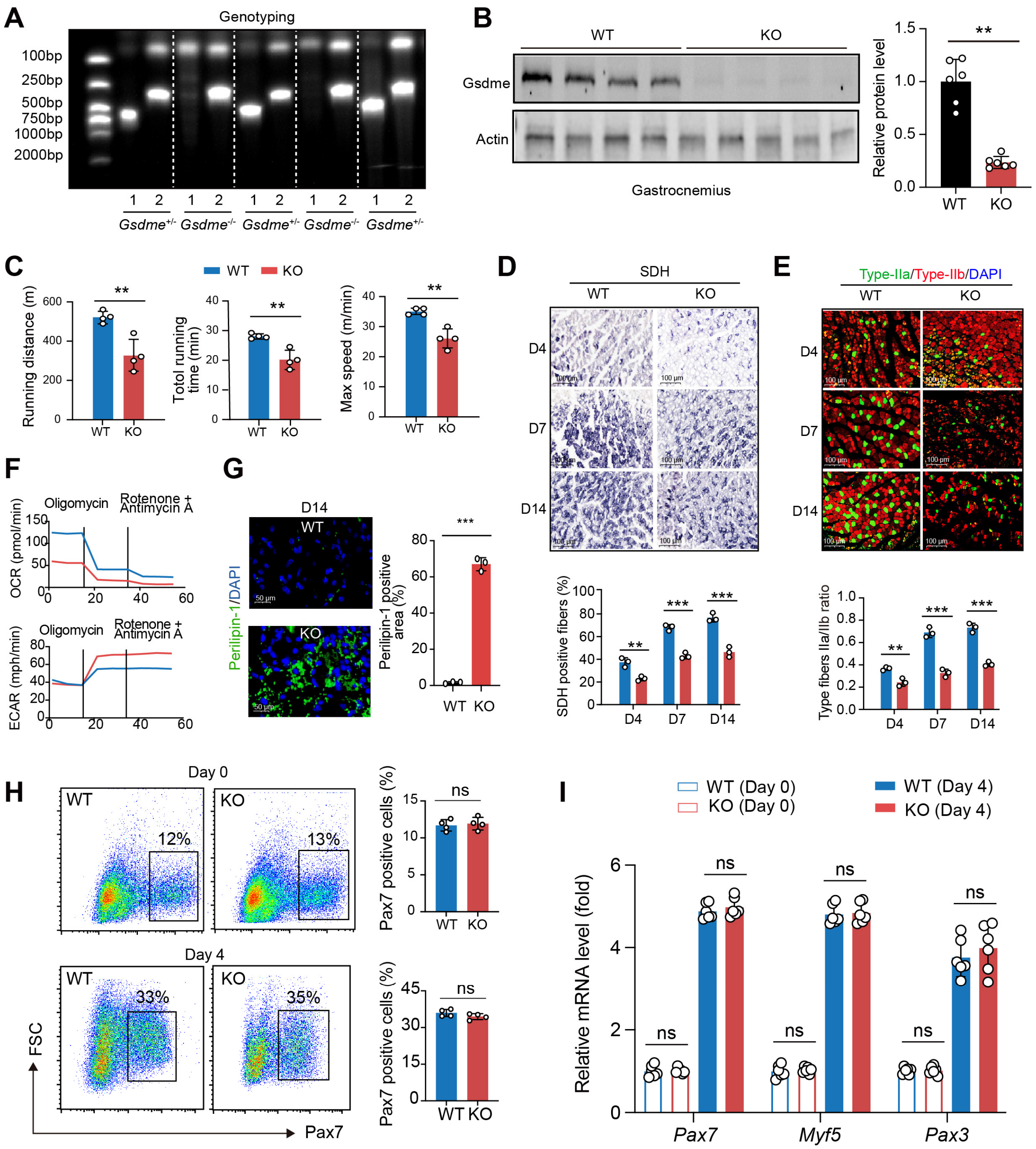

Supplemental Figure 2. Influence of GSDME deletion on exercise performance and ATP contents.

(A) Genotyping of *Gsdme*$^{+/-}$ and *Gsdme*$^{-/-}$ mice in agarose gel.

(B) Immunoblotting analysis confirmed the successful deletion of GSDME protein in skeletal muscle of *Gsdme*$^{-/-}$ mice.

(C) Exercise performance of WT and KO mice in a voluntary wheel running test at D14 post-injury.

(D) Representative images and quantification of SDH staining.

(E) Representative images and quantification of MyHC type-IIa and MyHC type-IIb fibers in the injured muscle using tyramide signal amplification (TSA)-based multiplex immunohistochemistry (dilution of anti-MyHC-type-IIa and anti-MyHC-type-IIb: 1:1,000).
(F) Metabolic profile analysis of injured skeletal muscle tissue using Seahorse technology. OCR and ECAR curves are presented (upper panel).
(G) Representative images of perilipin-1 immunofluorescence staining and quantification of lipid accumulation.
(H) Flow cytometry demonstrating the proportion of $Pax7^{+}$ cells in $CD45^{-}$ parenchymal cells of skeletal muscle of WT and KO mice at D0 and D4 post-injury.
(I) The mRNA levels of *Pax7*, *Myf5* and *Pax3* in mice skeletal muscle of WT and KO mice at D0 and D4 post-injury.
Data are presented as mean ± SEM. Unpaired Student's t-test between WT and KO were performed. $^{**}P<0.01$ KO vs WT. ns, no significance.

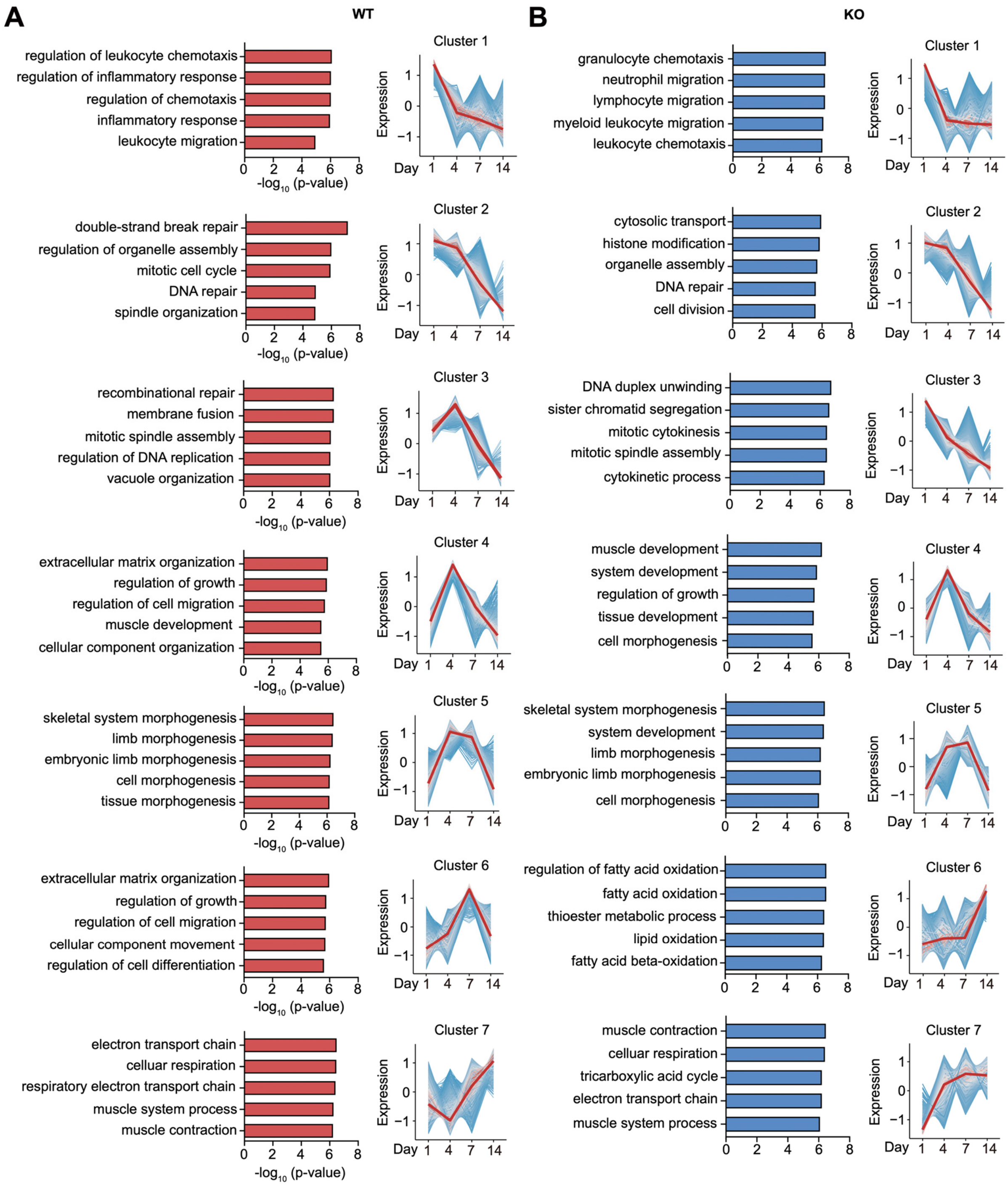

Supplemental Figure 3. Lipid metabolism and fat acid oxidation represent the most significantly altered molecular events during muscle repair in KO mice.

(A-B) Enriched biological functions in the seven clusters of gene signatures during skeletal muscle injury and repair processes in WT **(A)** and KO **(B)** mice. The Cluster 6 exhibits a noticeable difference between WT and KO mice, whereas the other clusters remain similar in both WT and KO mice.

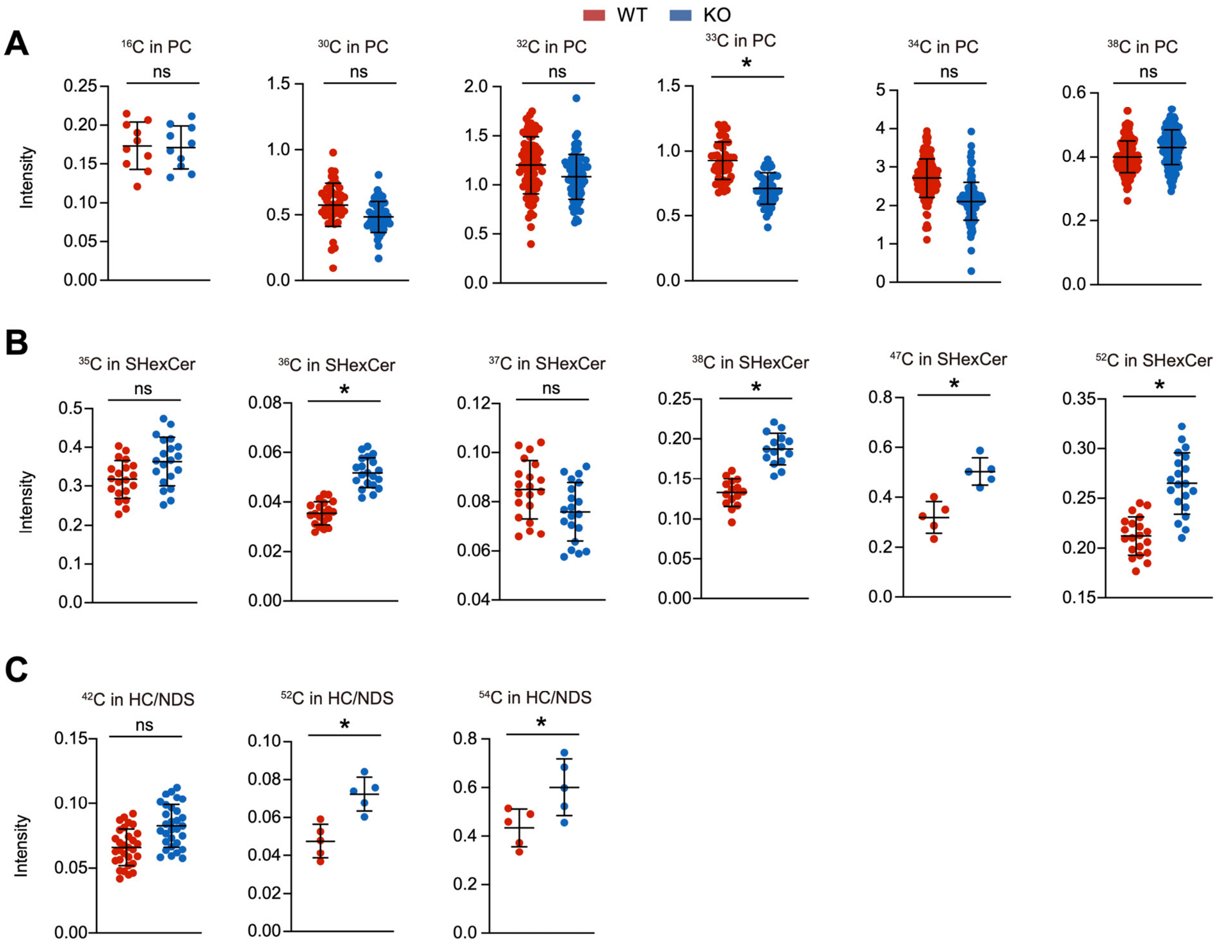

Supplemental Figure 4. Lipidomic study of injured skeletal muscle in WT and KO mice.

(A) Levels of PC (phosphatidylcholine) of short-chain fatty acids in skeletal muscles of WT and KO mice.

(B) Levels of SHexCer (sulfur hexosylceramide hydroxy fatty acid) in skeletal muscles of WT and KO mice.

(C) Levels of HC/NDS (hexosylceramide non-hydroxyfatty acid-dihydrosphingosine) in skeletal muscles of WT and KO mice.

Data are presented as mean ± SEM. Unpaired Student's t-test between WT and KO were performed. *P<0.05 KO vs WT. n=5 per group.

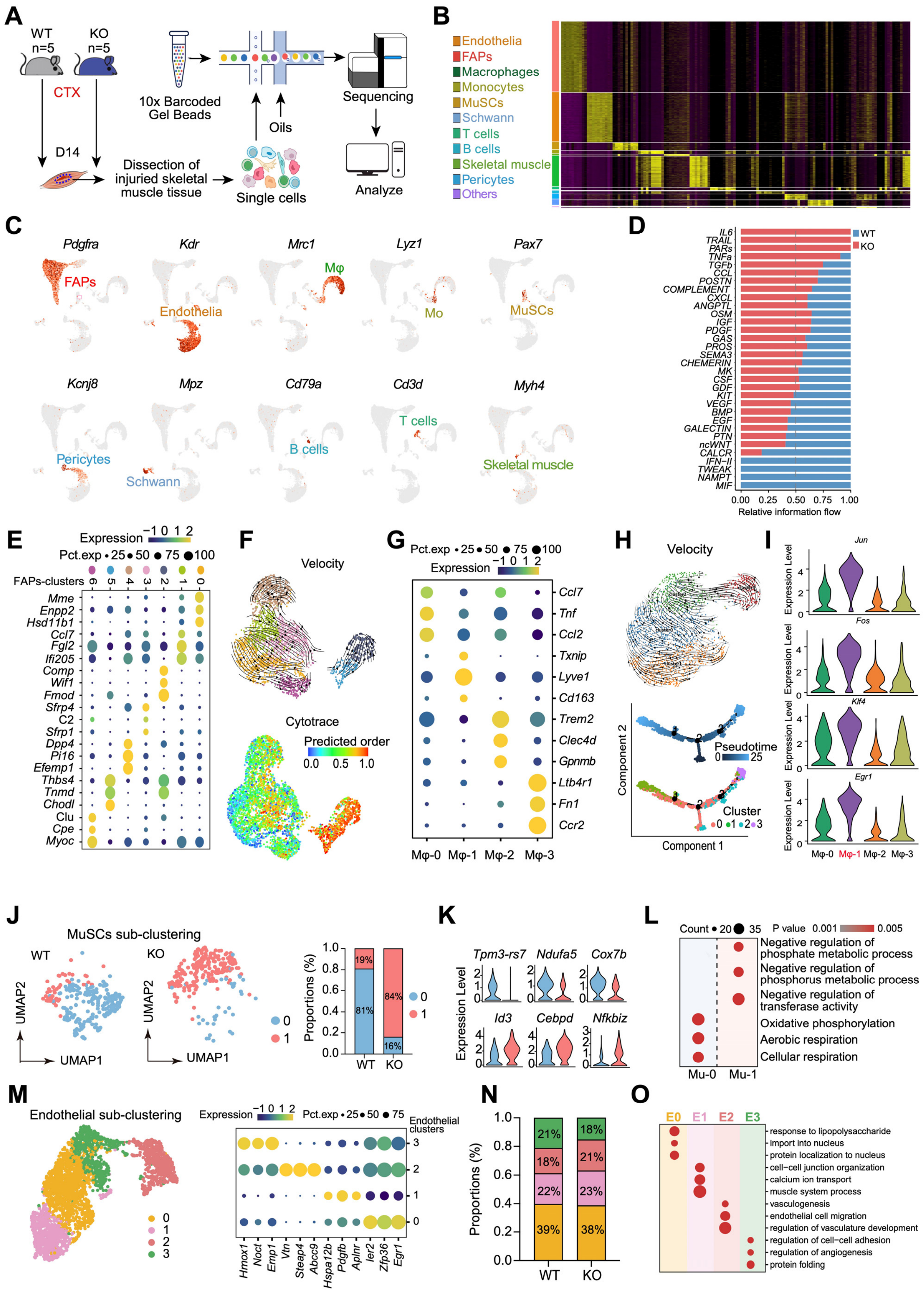
A
WT n=5
KO n=5
CTX
D14
10x Barcoded Gel Beads
Oils
Sequencing
Analyze
Dissection of injuried skeletal muscle tissue
Single cells
B
Endothelia
FAPs
Macrophages
Monocytes
MuSCs
Schwann
T cells
B cells
Skeletal muscle
Pericytes
Others
C
Pdgfra
Kdr
Mrc1
Lyz1
Pax7
Kcnj8
Mpz
Cd79a
Cd3d
Myh4
D
Relative information flow
E
FAPs-clusters
F
Velocity
Cytotrace
Predicted order
G
Mφ-0 Mφ-1 Mφ-2 Mφ-3
H
Velocity
Pseudotime
Cluster
Component 1
Component 2
I
Jun
Fos
Klf4
Egr1
Expression Level
J
MuSCs sub-clustering
Proportions (%)
K
Tpm3-rs7
Ndufa5
Cox7b
Id3
Cebpd
Nfkbiz
L
Negative regulation of phosphate metabolic process
Negative regulation of phosphorus metabolic process
Negative regulation of transferase activity
Oxidative phosphorylation
Aerobic respiration
Cellular respiration
M
Endothelial sub-clustering
Endothelial clusters
N
O
response to lipopolysaccharide
import into nucleus
protein localization to nucleus
cell−cell junction organization
calcium ion transport
muscle system process
vasculogenesis
endothelial cell migration
regulation of vasculature development
regulation of cell−cell adhesion
regulation of angiogenesis
protein folding

Supplemental Figure 5. scRNA-seq reveals the influences of GSDME deletion on the molecular status of different types of cells within injured muscle.

(A) Experimental design of scRNA-seq based on the 10x Genomics platform in the skeletal muscle tissues dissected from the CTX-injured site from WT and KO mice (n=5 per group).

(B) Heatmap showing the global expression pattern of marker genes in each cluster.

(C) Feature plot in UMAP showing the specific expression of marker genes in different cell types of injured skeletal muscle tissue.

(D) Summary of altered secreted factors for each listed pathway between WT and KO mice. The inhibited or induced secreted factors in KO muscle compared with WT muscle can be determined by the ratio of blue and red bars.

(E) Bubble plot showing the marker genes of the 7 sub-clusters of FAPs.

(F) Trajectory analysis of FAPs based on RNA Velocity and CytoTRACE pseudotime tools.

(G) Bubble plot showing the marker genes of the 4 sub-clusters of Mφ.

(H) Trajectory analysis of Mφ pseudotime order based on CytoTRACE and Monocle 2 tools.

(I) High expression of transcriptional factors, including Jun, Fos, Klf4, and Egr1 in the Mφ sub-cluster 1 (TRMs).

(J) UMAP and proportions of MuSC subclusters in injured skeletal muscle of WT and KO mice

(K) Marker genes of MuSC subgroups shown in a Violin plot.

(L) GO enrichment analysis of major biological functions in MuSC subclusters.

(M) Further sub-clustering of endothelia into 4 groups.

(N) Bubble plot showing the marker genes of the 4 sub-clusters of endothelia.

(O) Proportions of the 4 sub-clusters of endothelia in skeletal muscle of WT and KO mice.

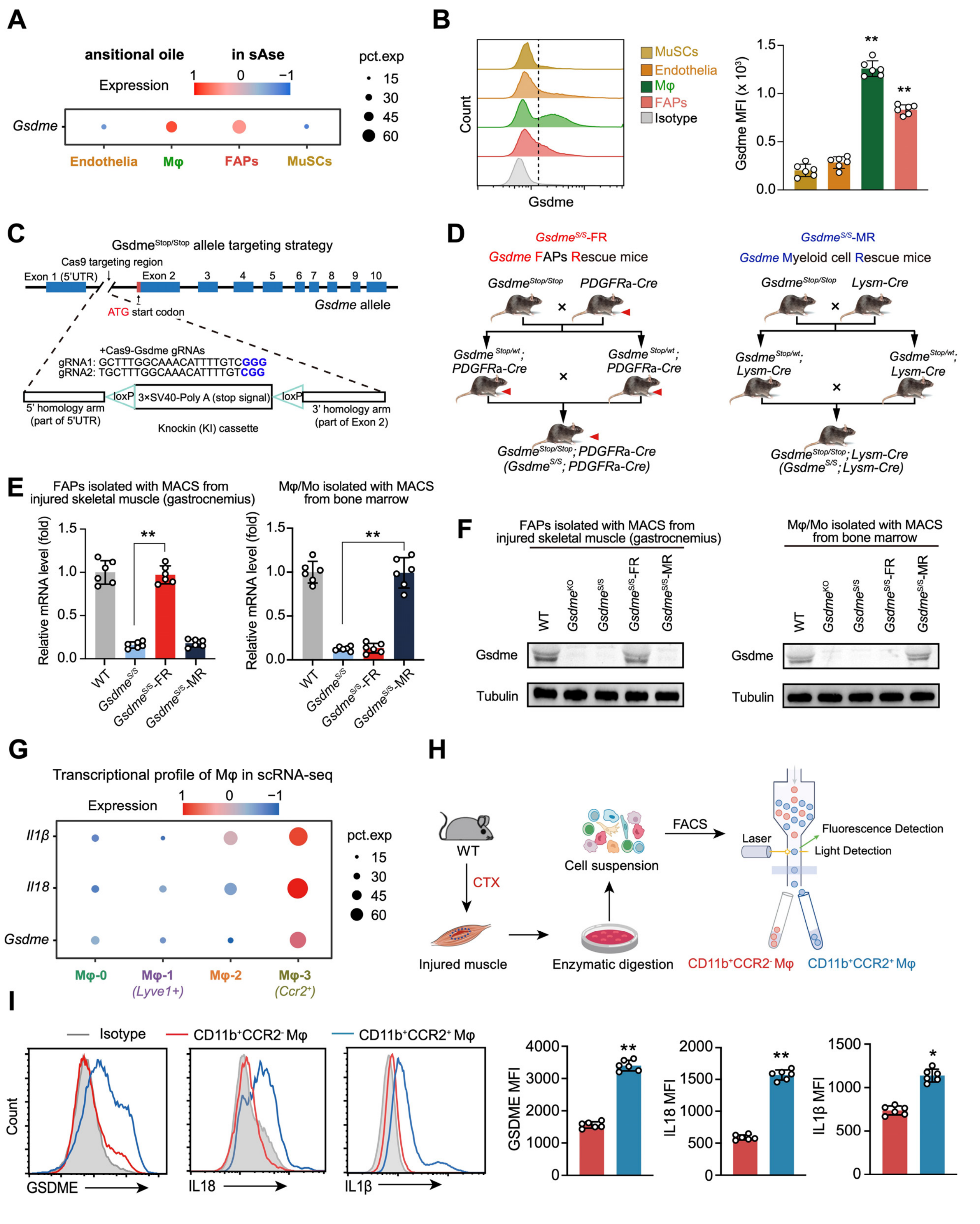

Supplemental Figure 6. Generation of conditional Gsdme rescue mice and scRNA/FACS analyses reveal cell-type specific expression with enrichment in CCR2⁺ macrophages

(A) Bubble plot showing the *Gsdme* expression of the Endothelia, Mφ, FAPs and MuSCs.

(B) Representative expression of *Gsdme* with corresponding mean fluorescence intensity (MFI) quantification (right panel) in Endothelia, Mφ, FAPs and MuSCs.

(C) Schematic diagram showing the gene targeting strategy for generating a mouse strain carrying a transcriptional Stop element flanked by loxP recombination sites (loxP-Stop-loxP, LSL) upstream of the ATG start codon of the Gsdme gene. The gRNA1 and gRNA2 to the mouse Gsdme gene, the donor vector containing the "part of 5'UTR-loxP-3*SV40-Poly A -loxP-part of E2" cassette, and Cas9 mRNA were co-injected into fertilized mouse eggs to generate targeted conditional knockin offspring (GsdmeStop/Stop). The sequences of gRNA1 and gRNA2 are also shown. The Stop element before the ATG start codon is expected to terminate the transcription of the Gsdme gene.

(D) The GsdmeStop/Stop mouse strain was crossed with *Pdgfa*-Cre or *Lysm*-Cre mouse to produce *Gsdme*$^{Stop/Stop}$;*Pdgfa*-Cre mouse (FAPs rescue of GSDME, referred as *Gsdme*$^{S/S}$-FR) or *Gsdme*$^{Stop/Stop}$;*Lysm*-Cre mouse (myeloid cell rescue of GSDME, referred as *Gsdme*$^{S/S}$-MR). The Cre expression specific to FAPs or myeloid cells can delete the Stop element to allow *Gsdme* re-transcription.

(E) qPCR analysis of GSDME mRNA levels in primary VSMCs and BMDMs from WT, *Gsdme*$^{-/-}$ (KO), *Gsdme*$^{S/S}$(*Gsdme*$^{Stop/Stop}$), *Gsdme*$^{S/S}$-FR and *Gsdme*$^{S/S}$-MR mice. GSDME protein was rescued in FAPs or Mφ/Mo. FAPs were isolated from injured skeletal muscle using MACS technology with the PDGFRα MicroBead Kit (Miltenyi Biotec 130-101-502). BMDMs were isolated from bone marrow of mice using MACS technology with the CD11b MicroBead Kit (Miltenyi Biotec 130-097-142).

(F) Immunoblotting analysis of GSDME protein levels in FAPs or Mφ/Mo from WT, *Gsdme*$^{-/-}$ (KO), *Gsdme*$^{Stop/Stop}$, *Gsdme*$^{S/S}$-FR and *Gsdme*$^{S/S}$-MR mice as in **(E)**. Tubulin was used as a loading control.

(G) Bubble plot showing the *Gsdme*, *Il18* and *Il1β* expression of the 4 sub-clusters of Mφ.

(H) Experimental design of FACS of CD11b^{+}CCR2^{+} and CD11b^{+}CCR2^{-} macrophages in the skeletal muscle tissues dissected from the CTX-injured site from WT mice (n=6).

(I) Representative expression of *Gsdme, Il18*, and *Il1b* (left panel) with corresponding mean fluorescence intensity (MFI) quantification (right panel) in FACS-sorted CD11b^{+}CCR2^{+} and CD11b^{+}CCR2^{-} macrophages.

Data were presented as mean ± SEM. ANOVA analysis followed by Tukey hoc-test was performed. *P<0.05, **P<0.01, KO vs WT. ns, no significance.

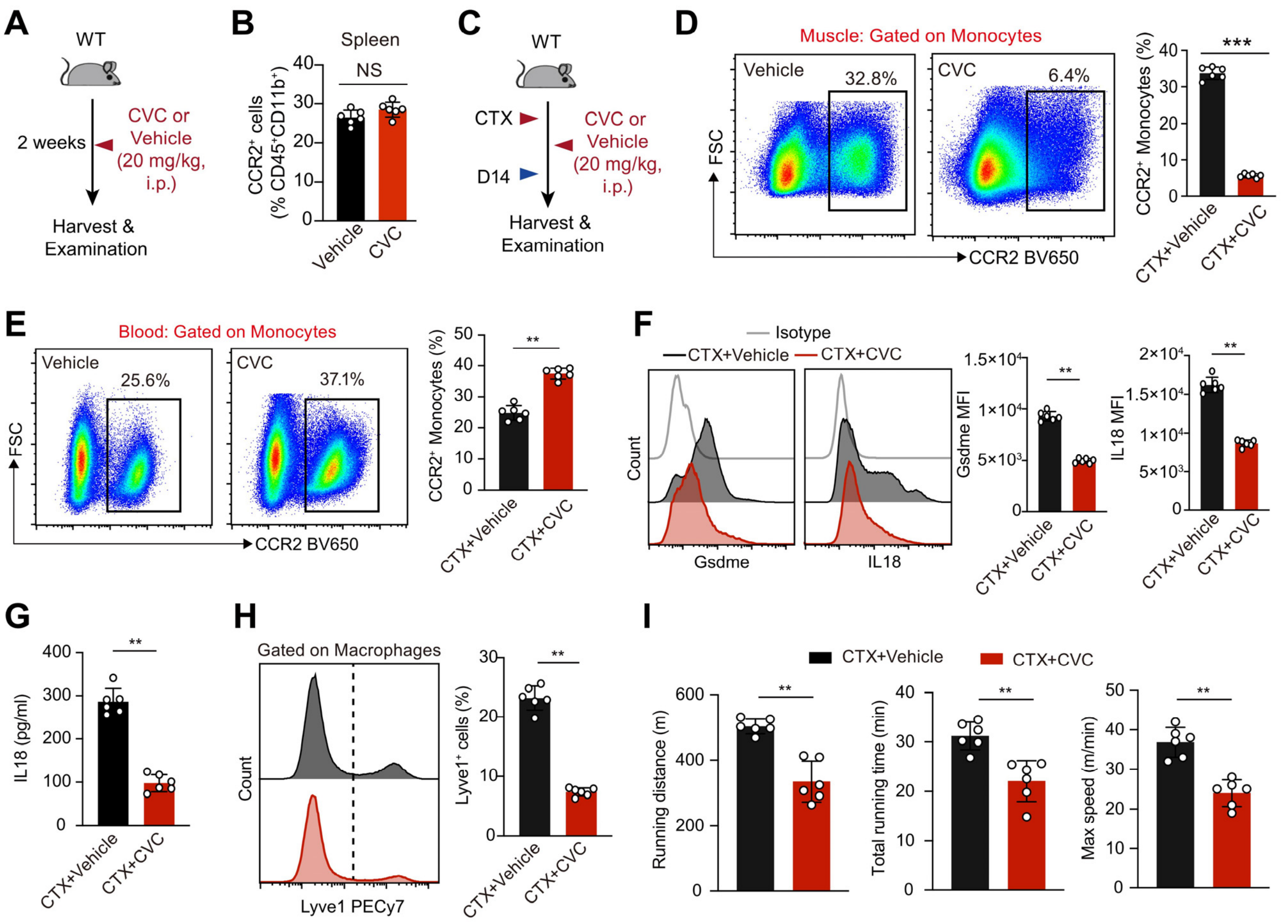

Supplemental Figure 7. Blocking monocyte recruitment to muscle impairs the GSDME-IL18 axis and prevents effective regeneration.

(A) Experimental design for the C-C chemokine receptors type 2 antagonist cenicriviroc treatment study. WT mice were administered daily with either the CVC or vehicle for 14 days. Mice were sacrificed at day 14, and the spleen were collected for flow cytometry.

(B) Splenocytes isolated from WT mice were treated with either the CVC or vehicle. Flow cytometry was used to determine the percentage of $CCR2^+$ cells within $CD45^+$ $CD11b^+$ cells in the spleen.

(C) Schematic of the experimental design for CVC treatment. WT mice received CTX injections to induce muscle injury, followed by daily administration of either CVC or vehicle for 14 days. On day 14 post-CTX injection, mice were sacrificed, and muscle and blood samples were collected for flow cytometry.

(D) Representative flow cytometry plots and quantification showing the proportion of $CCR2^+$ cells among monocytes isolated from the skeletal muscle of CTX-injured mice treated with vehicle or CVC.

(E) Representative flow cytometry plots and quantification showing the proportion of $CCR2^+$ cells among monocytes isolated from the blood of CTX-injured mice treated with vehicle or

CVC.

(F) Representative flow cytometry plots showing Gsdme and Il18 expression, with corresponding mean fluorescence intensity (MFI) quantification (right panel) in CTX-injured mice treated with vehicle or CVC.

(G) Quantification of IL-18 levels in skeletal muscle by ELISA in CTX-injured mice treated with vehicle or CVC.

(H) Flow cytometric analysis showing the proportion of Lyve1$^+$ macrophages in CTX-injured mice treated with vehicle or CVC.

(I) Exercise performance of CTX-injured mice treated with vehicle or CVC, assessed by voluntary wheel running at day 14 post-injury.

Data are presented as mean ± SEM. Unpaired Student's t-test was performed between vehicle- and CVC-treated groups in CTX-injured mice. ***P*<0.01, ****P*<0.001. ns, no significance.

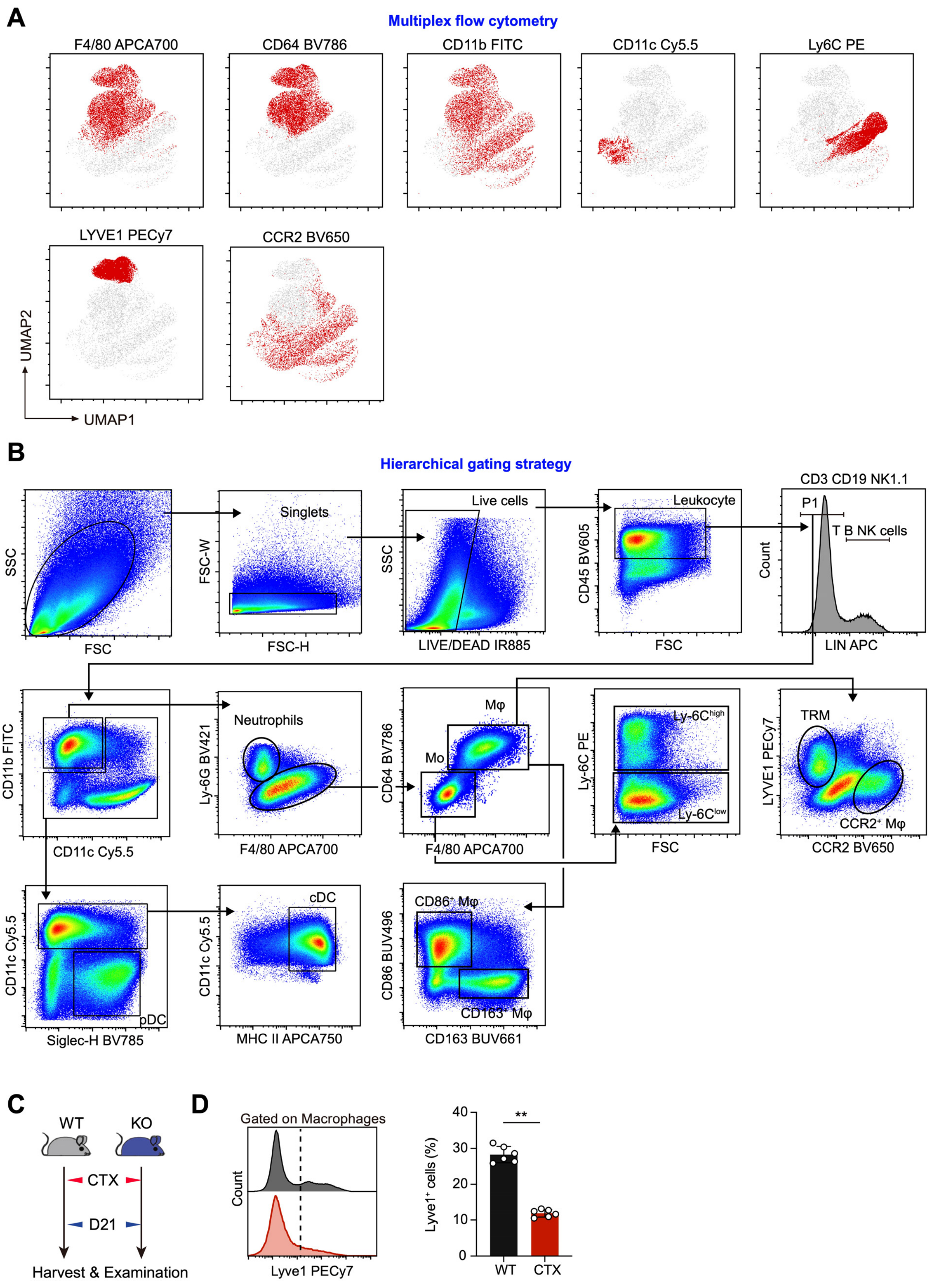

Supplemental Figure 8. Flow cytometry analysis of intramuscular immune cell reprogramming

(A) High-dimensional analysis of multiplexed flow cytometry using an unbiased UMAP

algorithm to identify subpopulation clustering in intramuscular CD45^{+} cells gated with antibodies (F4/80-APCA700, CD64-BV786, CD11b-FITC, CD11c-PercpCy5.5, Ly6C-PE, LYVE1-PECy7 and CCR2-BV650). The multiplexed flow cytometry results were concatenated, transformed, and plotted in 2D UMAP plots using R software.

(B) Gating strategy for identifying distinct immune cell subsets in the injured muscle tissues of mice.

(C) Experimental design for the CTX-induced muscle injury model in WT and GSDME-KO mice.

(D) Flow cytometric analysis showing the proportion of Lyve1^{+} macrophages.

Data are presented as mean ± SEM. Unpaired Student's t-tests between WT and KO were performed. ***P*<0.01, KO vs WT.

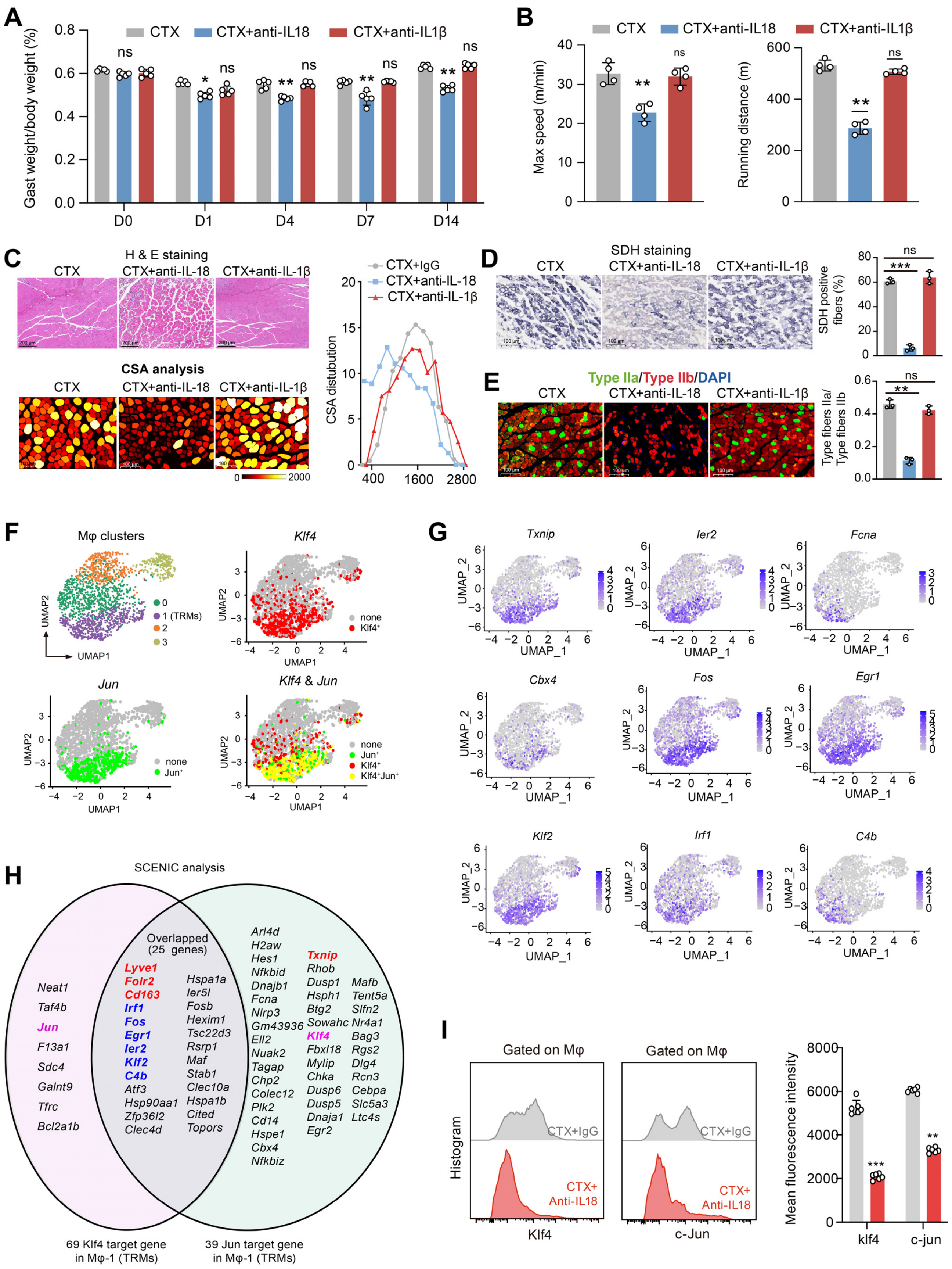
A
CTX
CTX+anti-IL18
CTX+anti-IL1β
Gast weight/body weight (%)
D0
D1
D4
D7
D14
ns
B
Max speed (m/min)
Running distance (m)
C
H & E staining
CTX
CTX+anti-IL-18
CTX+anti-IL-1β
CSA analysis
CTX+IgG
CSA distubution
D
SDH staining
SDH positive fibers (%)
E
Type IIa/Type IIb/DAPI
Type fibers IIa/ Type fibers IIb
F
Mφ clusters
1 (TRMs)
UMAP1
UMAP2
Klf4
Jun
Klf4 & Jun
none
Klf4⁺
Jun⁺
Klf4⁺Jun⁺
G
Txnip
Ier2
Fcna
Cbx4
Fos
Egr1
Klf2
Irf1
C4b
UMAP_1
UMAP_2
H
SCENIC analysis
Overlapped (25 genes)
Neat1
Taf4b
Jun
F13a1
Sdc4
Galnt9
Tfrc
Bcl2a1b
Lyve1
Folr2
Cd163
Irf1
Fos
Egr1
Ier2
Klf2
C4b
Atf3
Hsp90aa1
Zfp36l2
Clec4d
Hspa1a
Ier5l
Fosb
Hexim1
Tsc22d3
Rsrp1
Maf
Stab1
Clec10a
Hspa1b
Cited
Topors
Arl4d
H2aw
Hes1
Nfkbid
Dnajb1
Fcna
Nlrp3
Gm43936
Ell2
Nuak2
Tagap
Chp2
Colec12
Plk2
Cd14
Hspe1
Cbx4
Nfkbiz
Txnip
Rhob
Dusp1
Hsph1
Btg2
Sowahc
Klf4
Fbxl18
Mylip
Chka
Dusp6
Dusp5
Dnaja1
Egr2
Mafb
Tent5a
Slfn2
Nr4a1
Bag3
Rgs2
Dlg4
Rcn3
Cebpa
Slc5a3
Ltc4s
69 Klf4 target gene in Mφ-1 (TRMs)
39 Jun target gene in Mφ-1 (TRMs)
I
Gated on Mφ
Histogram
CTX+IgG
CTX+ Anti-IL18
Klf4
c-Jun
Mean fluorescence intensity
klf4
c-jun

Supplemental Figure 9. KLF4 and JUN co-regulate tissue-resident macrophages identity and modulate muscle repair.

(A) Gastrocnemius muscle weight to body weight ratio in CTX-injured mice treated with neutralizing antibodies against IL-18, IL-1β, or normal IgG.

(B) Maximum speed and running distance in exercise performance of CTX-injured mice treated with neutralizing antibodies against IL-18, IL-1β, or normal IgG.

(C) Representative HE staining and quantitative CSA analysis of injured muscle with CTX+IgG, CTX+anti-IL1β, and CTX+anti-IL-18.

(D) Representative SDH staining and quantification in the same treatment groups.

(E) Representative immunofluorescence of MyHC type IIa and type IIb fibers and quantitative analysis of their ratio in injured muscle under the indicated treatments.

(F) Restricted expression of *Klf4* and *Jun* in the Mφ sub-cluster 1 and their co-expression.

(G) Venn plot showing the 33 KLF4 target genes and 68 JUN target genes in Mφ-1 (TRMs). Among them, 25 genes, including several well-known marker genes of TRMs such as *Lyve1, Folr2,* and *Cd163*, are target genes for both KLF4 and JUN.

(H) Expression pattern of nine genes (*Txnip, Ier2, Fcna, Cbx4, Fos, Egr1, Klf2, Irf1,* and *C4b*), which are target genes of both KLF4 and JUN in the UMAP plot.

(I) Proportions of Mφ positive for Klf4 or Jun in total Mφ were determined by flow cytometry. In-direct flow cytometry of Klf4 or Jun was performed in the $CD11b^+$ Mφ isolated from injured muscle tissue using MACS technology.

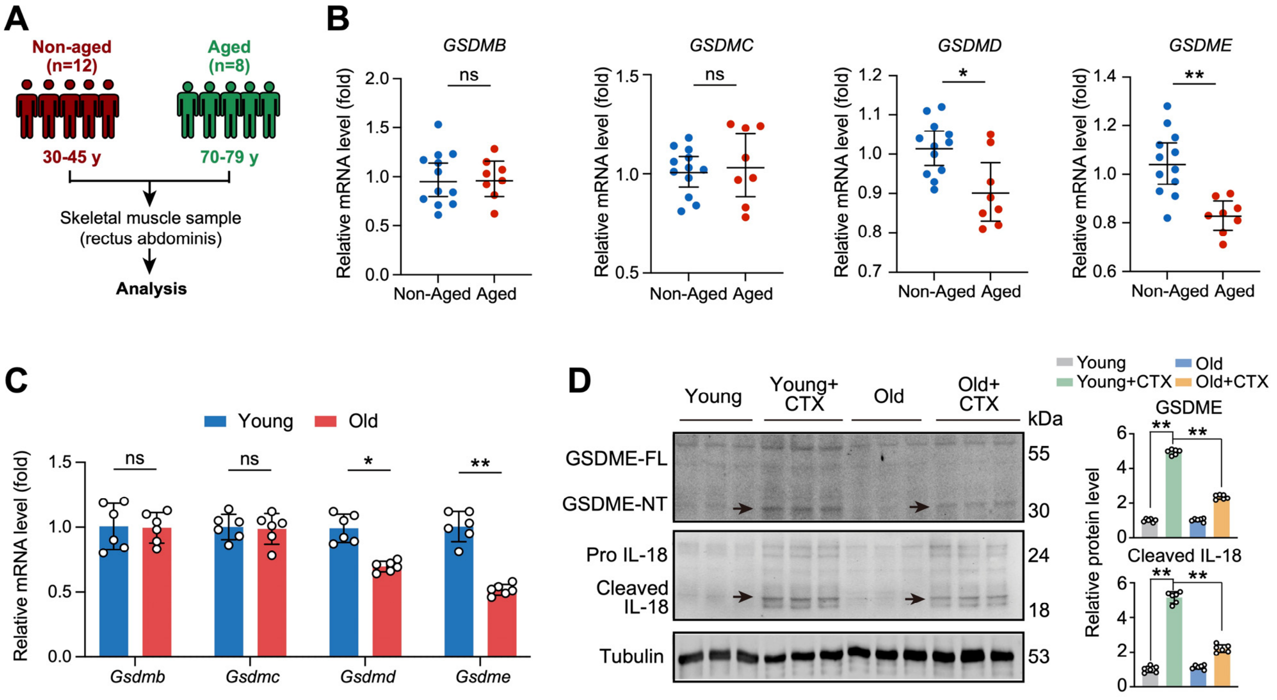

Supplemental Figure 10. GSDME-IL18 pyroptosis pathway is impaired in aged human and murine muscle.

(A) Study design for analyzing the role of GSDME in skeletal muscle samples obtained during rectus abdominal surgery from two age groups: young adults aged 30-45 years (n=12) and elderly individuals aged 70-79 years (n=8).

(B) Comparison of transcriptome profiles of gasdermin family members (*GSDMB, GSDMC, GSDMD,* and *GSDME*) between young and old individuals.

(C) Comparison of transcriptome profiles of gasdermin family members (*GSDMB, GSDMC, GSDMD,* and *GSDME*) between 8-week-old young and 24-month-old mice.

(D) Western blotting analysis of the cleaved N-terminal of GSDME and cleaved IL-18 in injured skeletal muscle of 8-week-old young and 24-month-old mice at Day 4 post-injury. Arrows indicate the cleaved GSDME and IL18 (active form)

Data are presented as mean ± SEM. Unpaired Student's t-tests between WT and KO were performed. *P<0.05, **P<0.01, KO vs WT. ns, no significance.

Table S1. Differentially Expressed Genes (DEGs) at four timepoints (D4, D7 and D14) post-injury in injured muscle between WT and KO mice

Gene name	WT-1 (Tpm)	WT-2 (Tpm)	WT-3 (Tpm)	KO-1 (Tpm)	KO-2 (Tpm)	KO-3 (Tpm)	Fold Change	*P* value	Timepoint (post-injury)
Gm38182	7 75	9 05	5 34	1 35	1 05	1 17	0 261336516	8 76475E-08	Day 4
Tmem181b-ps	11 35	8 27	6 73	4 61	2 19	3 83	0 46439523	1 37114E-06	Day 4
Nuak2	9 25	7 4	5 74	3 37	1 89	3 27	0 454115794	1 82527E-06	Day 4
Junb	118 69	78 44	82 42	49 62	18 56	34 36	0 373526809	4 3997E-06	Day 4
Gm43951	0 14	0 11	0 06	0 83	0 38	0 46	1 410876133	8 08581E-06	Day 4
Arrdc2	10 83	8 05	7 09	15 12	78 77	33 54	4 5022437	1 5914E-05	Day 4
Nr4a3	1 68	1 47	2 21	3 85	3 48	4 06	1 721291866	1 60516E-05	Day 4
Pck1	3 27	2 43	1 21	6 57	20 87	5 37	3 613521695	2 25313E-05	Day 4
Retnla	49 06	117 68	89 45	416 63	137 18	265 85	3 173965045	2 93592E-05	Day 4
Ppl	0 34	0 18	0 23	0 69	0 9	0 54	1 368	4 50369E-05	Day 4
Rgs6	0 09	0 36	0 25	0 64	0 53	0 67	1 308108108	5 21052E-05	Day 4
Gm37240	0 03	0	0	6 01	3 09	0 12	4 0330033	6 29387E-05	Day 4
Egr1	19 53	43 03	39 95	12 56	5 2	9 8	0 289640792	7 23003E-05	Day 4
Il1b	28 24	6 35	5 98	2 95	0 31	1 35	0 174661464	7 56117E-05	Day 4
Gm10645	1 2	1 11	1 58	0	0 13	0	0 454281567	0 000111198	Day 4
Myo5c	0 47	0 65	0 94	1 72	4 08	1 63	2 061264822	0 000116878	Day 4
Amy1	2 03	1 83	1 6	3 27	6 42	4 49	2 030732861	0 000131855	Day 4
Tmem266	0 23	0 32	0 31	0 43	2 01	1 55	1 810880829	0 000147995	Day 4
3230206H07Rik	0 15	0 11	0 15	0 51	1 08	0 35	1 448680352	0 000154385	Day 4
Fos	17 81	29 9	31 57	13 62	4 66	7 09	0 34479825	0 000187285	Day 4
Olfr78	0 37	0 37	0 36	1 3	1 21	0 73	1 52195122	0 000188863	Day 4
Ces1d	4 91	2 85	2 93	4 08	17 39	5 97	2 223520818	0 000201023	Day 4
Casr	0 41	0 41	0 6	0 95	1 44	1 17	1 484162896	0 000206322	Day 4
Cyp2e1	8 64	8 5	5 82	11 91	61 11	17 63	3 607473035	0 000250844	Day 4
Gm49380	0 18	0 15	0 06	0 47	1 12	0 34	1 454277286	0 000262344	Day 4
Tspan8	6 98	11 98	8 71	18 25	22 41	19 72	2 066514509	0 000322953	Day 4
Ppp1r3c	23 83	55 65	59 18	89 64	236 86	145 13	3 350487082	0 000341716	Day 4
Cdo1	5 83	3 21	2 4	7 19	16 02	6 58	2 270775623	0 000341997	Day 4
Dach2	0 03	0	0 03	0 2	0 52	0 08	1 241830065	0 000377178	Day 4
Scd1	60 15	37 29	22 27	88 14	106 03	61 09	2 104636949	0 000380562	Day 4
Prss2	0 1	0 09	0 15	0	8 79	1 26	3 907185629	0 000430604	Day 4

Pappa2	0 68	0 07	0 44	0 88	0 57	0 6	1 205250597	0 000430764	Day 4
Amigo2	2 23	1 31	1 24	3 49	3 84	2 21	1 611825193	0 000541283	Day 4
Fmo2	1 31	1 98	1 95	3 91	5 89	3 22	1 944174757	0 000565182	Day 4
Gm54759	0 73	0 61	0 41	0 93	1 92	1 5	1 547368421	0 000602442	Day 4
Gpr183	6 12	4 1	3 76	2 1	0 56	1 97	0 449352179	0 000623475	Day 4
Mup14	0 36	0 45	0 12	1 26	3 24	1 07	2 180661578	0 000641337	Day 4
Pnlip	0 04	0 04	0	0	3 15	0 62	2 198051948	0 00065104	Day 4
Acer2	1 01	0 68	0 78	1 63	3 02	1 43	1 659963437	0 00065371	Day 4
mt-Co2	89 43	101 68	193 19	285 29	313 03	276 08	2 265427317	0 000716816	Day 4
Gm12928	3 11	1 89	2 32	0 35	0	0	0 324612403	0 000724291	Day 4
Thrsp	12 17	24 82	16 25	32 17	55 05	40 74	2 32859175	0 000748652	Day 4
Chac1	2 42	3 79	4 63	8 45	7 05	7 02	1 843930636	0 00077283	Day 4
Inmt	1 7	1 01	2 14	3 54	11 88	3 04	2 733757962	0 00087088	Day 4
Mstn	3 82	3 87	2 64	4 26	9 25	9 24	1 931732933	0 000880302	Day 4
Zfp969	1 56	1 81	1 67	3 59	2 45	3 33	1 538557214	0 000905074	Day 4
Erdr1	17 81	31 56	43 37	80 56	48 65	71 8	2 130875287	0 000951826	Day 4
Smco3	0 22	0 1	0 23	0 61	0 86	0 52	1 405633803	0 000955172	Day 4
Fam107a	0 53	0 94	0 86	1 86	4 23	0 8	1 855534709	0 001041681	Day 4
F5	0 79	0 45	0 34	1 1	0 51	1 05	1 23580786	0 001168378	Day 4
Cfd	281 19	124 83	92 79	267 41	978 08	264 38	3 014826329	0 001178114	Day 4
Ddit4l	17 83	29 16	34 04	50 52	99 87	60 09	2 540521242	0 00118838	Day 4
BC049352	0 19	0 35	0 11	0 71	1 54	0 94	1 695890411	0 001191663	Day 4
Sult1a1	9 41	8 41	6 96	13 68	24 54	12 51	1 93412527	0 001201405	Day 4
Cel	0 19	0	0 02	0 07	1 63	0 41	1 591900312	0 001217224	Day 4
Azin2	14 39	12 16	10 9	6 83	2 19	5 63	0 436341162	0 001231419	Day 4
Adhfe1	2 22	3 11	1 94	4 28	12 58	4 19	2 341772152	0 001237965	Day 4
Aldh1a7	0 42	0 41	0 3	0 59	2 43	0 98	1 694915254	0 001336551	Day 4
Shisa3	0 24	0 26	0 47	0 74	1 74	0 66	1 546599496	0 001432315	Day 4
Klhl33	3 22	5 26	6 43	7 07	25 5	12 55	2 686767169	0 001459029	Day 4
C130080G10Ril	0 8	1 6	1 35	1 75	3 6	4 06	1 838518519	0 001533076	Day 4
Tfcp2l1	0 14	0 25	0 23	0 37	0 85	0 49	1 301104972	0 001549046	Day 4
Gm32200	0 31	0 57	1 09	1 22	4 9	1 5	2 136820926	0 0015555	Day 4
Lyve1	5 77	2 79	5 85	10 62	9 91	6 35	1 716255026	0 001567428	Day 4
Slc25a25	18 16	21 5	16 01	21 15	94 74	41 49	2 733594682	0 001568003	Day 4
Timp4	0 78	0 31	4 32	4 78	18 01	3 86	3 525564804	0 001581178	Day 4
Gm43857	1 07	0 88	0 51	0 28	0 21	0 18	0 672161172	0 001625045	Day 4
Cacna1b	0 9	0 13	0 42	0 15	0 1	0 24	0 784269663	0 001645865	Day 4
Capn11	0 35	0 62	0 76	0 15	0 04	0	0 674418605	0 001651331	Day 4

Trem1	2 37	0 16	0 08	0 08	0	0 03	0 554367201	0 001717359	Day 4
Scara5	10 75	12 05	15 81	25 92	22 08	18 07	1 659937515	0 001787145	Day 4
Klf15	2 34	5 04	5 34	8 22	21 53	8 61	2 631043257	0 001888743	Day 4
Mcf2l	6 4	5 47	8 25	11 31	12 99	9 08	1 573529412	0 001903991	Day 4
Grem2	2 9	1 96	1 63	2 67	4 03	4 61	1 507903056	0 001916373	Day 4
Cpb1	0 02	0	0 05	0 05	0 83	0 16	1 315960912	0 001973062	Day 4
Mup9	1 32	0 08	0 1	3 45	6 8	3 04	3 62	0 002108953	Day 4
Smpd3	0 66	0 72	0 49	0 91	2 06	0 96	1 422997947	0 002136866	Day 4
mt-Rnr2	78 11	114 63	103 66	144 61	541 94	159 63	2 836272545	0 002148066	Day 4
Teddm2	0 12	0 21	0 22	0 43	0 46	0 48	1 230985915	0 002178825	Day 4
Mylk4	16 6	19 93	22 56	19 98	85 17	51 88	2 577387663	0 002191317	Day 4
Kcna5	1 95	2 71	3 77	6 22	12 9	4	2 285214348	0 00220221	Day 4
Alkal2	0 04	0 32	0 21	0 81	1 31	0 55	1 588235294	0 002240503	Day 4
Ocln	0 32	0 03	0 08	0 86	0 42	0 19	1 303206997	0 002265876	Day 4
Gpt2	36 23	38 35	38 02	48 58	80 36	72 51	1 768598616	0 002376792	Day 4
Pnpla3	0 51	1 16	0 99	1 93	5 88	1 5	2 174911661	0 002420871	Day 4
Rin1	10 28	4 98	5 87	3 47	0 81	1 6	0 368006631	0 002429752	Day 4
Mn1	4 12	5 44	6 19	9 29	11 65	7 69	1 686933333	0 002447776	Day 4
Svs5	0 14	0	0	0	5 6	0 62	2 936305732	0 002461958	Day 4
Slc15a5	0 35	0 05	0 2	0 68	2 84	0 14	1 85	0 002632581	Day 4
Xpnpep2	0 48	0 35	0 69	1 94	1 19	2 06	1 811946903	0 002640456	Day 4
Garem2	0 96	0 5	0 37	0 25	0 03	0 11	0 701863354	0 002733419	Day 4
Slc9a3	0 06	0 04	0 03	0 1	0 45	0 13	1 17571885	0 002780447	Day 4
Ptprz1	0 12	0 11	0 07	0 25	0 27	0 21	1 13030303	0 002899456	Day 4
Mup22	14 82	5 2	1 62	15 79	27 46	16 24	2 53612013	0 002908679	Day 4
Gm21860	3 08	1 32	1 44	4 38	1 83	3 98	1 492081448	0 002969832	Day 4
Gm2007	1 46	0 64	2 92	0 36	5 2	0 08	1 077306733	0 003024937	Day 4
Lrrc30	22 36	37 98	34 58	47 85	77 93	60 49	1 932904412	0 003042274	Day 4
Gm20662	0	0	0 4	0 49	0 55	1 43	1 608823529	0 003052221	Day 4
Gm47283	35 5	66 69	59 14	123 29	61 11	104 24	1 774721597	0 003061375	Day 4
Cytl1	2 83	2 18	2 91	5 44	15 59	2 91	2 467032967	0 00309267	Day 4
Ankrd9	24 2	40 18	41 14	45 01	82 8	86 98	2 006911168	0 0031328	Day 4
Gm26740	7 21	7 03	5 37	7 96	11 43	7 47	1 320654578	0 003164929	Day 4
Ccng1	75 32	102 95	70 6	112 03	149 84	147 49	1 63719379	0 003220691	Day 4
Pde4d	8 86	12 61	16 29	21 17	35 42	23 89	2 048086359	0 003276892	Day 4
A530016L24Rik	0 73	0 31	0 35	0 57	3 11	1 25	1 806378132	0 00330978	Day 4
Ces1f	0 45	0 39	0 33	0 54	2 05	1 28	1 647482014	0 003422955	Day 4
Gm38102	0	0	0	0 42	0 45	0	1 29	0 003475374	Day 4

Prss3	0	0	0	0	1 6	0 81	1 803333333	0 00348199	Day 4
Mcpt4	17 01	10 64	12 97	20 24	25 56	18 21	1 536221917	0 003564118	Day 4
Mlf1	19 68	59 61	45 19	70 15	151 81	92 49	2 49019454	0 003576914	Day 4
2310047D07Rik	1 48	3 51	2 96	4 64	13 81	5 5	2 461187215	0 003580154	Day 4
Aldh6a1	13 33	15 65	15 48	21 37	28 84	21 79	1 580278129	0 003593538	Day 4
Tlcd4	0 66	0 94	1 05	1 15	2 78	1 88	1 559292035	0 003669453	Day 4
Carnmt1	14 49	27 55	11 95	21 78	28 87	21 88	1 325320232	0 003677104	Day 4
Kcna2	0 18	0 22	0 22	0 4	0 8	0 25	1 229281768	0 003690841	Day 4
Npy1r	1 08	1 25	0 49	1 5	2 65	1 78	1 534364261	0 003713907	Day 4
Mrln	63 37	61 73	52 79	71 24	100 94	103 64	1 541378738	0 003765641	Day 4
Cdnf	2 64	4 3	4 93	6 38	11 05	7 45	1 874915938	0 003868335	Day 4
Cxcl13	0 3	0 27	0 09	0 67	0 87	0 75	1 445355191	0 003941272	Day 4
Ostn	0 42	1 6	1 33	1 54	7 37	3 24	2 385826772	0 003952081	Day 4
Rnf212b	0 15	0 13	0 68	1 02	5 49	0 51	2 53030303	0 004042913	Day 4
9330159F19Rik	2 03	2 6	2 79	3 05	5 55	5 05	1 597888676	0 004066088	Day 4
Lrrc38	4 11	7 19	7 69	8 78	28 88	11 51	2 372442019	0 004071854	Day 4
Gapdh-ps16	0 56	1 23	1 49	2 54	2 7	2 62	1 729299363	0 004094725	Day 4
Ass1	38 54	14 79	14 21	11 25	3 43	8 65	0 373263397	0 004130341	Day 4
Klf9	9 14	15 11	11 44	17 63	22 61	19 67	1 626001551	0 004175854	Day 4
Tdrp	3 97	3 27	1 99	3 27	7 26	6 26	1 618152085	0 004202342	Day 4
Misp3	1 79	2 88	1 67	2 04	5 16	5 32	1 661670236	0 004203038	Day 4
Sar1b	96 17	137 5	134 94	166 89	336 8	216 21	1 945319017	0 004211392	Day 4
Prss53	0 2	0	0 34	0 83	0 98	0 76	1 573446328	0 004271297	Day 4
Plcd4	7 04	11 52	13 81	10 2	52 25	25 96	2 584393554	0 004396234	Day 4
Hlf	0 9	0 86	0 46	0 93	1 16	1 18	1 201149425	0 004398036	Day 4
Lao1	0	0	0	0 04	0 16	0 34	1 18	0 004434428	Day 4
Bmp3	0 38	0 2	0 28	0 74	0 45	0 51	1 21761658	0 004444175	Day 4
1700025L06Rik	4 64	1 49	3 55	1 21	0 23	0 89	0 420347003	0 004444262	Day 4
Gm8116	0 38	0 49	0 4	0 47	2 17	3 72	2 192037471	0 004455546	Day 4
2310065F04Rik	4 01	5 27	7 31	4 14	57 59	9 3	3 778968862	0 004526312	Day 4
Adig	3 09	0 14	1 5	4 54	15 85	2 27	3 319534282	0 004527075	Day 4
Gm5884	2 16	4 49	4 86	7 93	5 9	8 23	1 727084769	0 004535099	Day 4
Mup18	6 16	2 01	0 65	5 46	13 2	6 59	2 39001692	0 00454624	Day 4
Gm47441	97 11	0	68 41	201 22	126 7	582 32	5 419178732	0 004658177	Day 4
Mettl21e	0 04	0 57	0 28	0 6	1 93	1 02	1 683804627	0 004661793	Day 4
Gm49477	2 19	4 32	2 56	4 76	13 13	5 56	2 191383596	0 004682689	Day 4
Phkg1	38 95	71 67	86 89	89 13	226 12	147 36	2 322128572	0 004688414	Day 4
Gm44850	6 22	3 66	5 25	1 03	0	1 14	0 285162714	0 004727135	Day 4

Myh4	1563 68	2631 18	2448 29	2566 34	5362 39	5538 7	2 026801983	0 004819647	Day 4
Gm15543	3 11	5 5	14 36	16 14	95 99	6 06	4 666538313	0 00482033	Day 4
Alb	0 4	0 31	0 09	0 22	2 53	0 49	1 642105263	0 004822535	Day 4
Ybx2	0 7	1 3	1 47	2 32	6 9	1 54	2 126738794	0 004824159	Day 4
Upp1	23 43	6 29	9 89	8 54	2 14	3 57	0 404834546	0 004840445	Day 4
Cidec	14 58	4 94	3 33	10 14	36 32	10 91	2 335396518	0 004854617	Day 4
Micu3	1 73	1 76	0 97	2 32	2 18	3 3	1 44772118	0 00493842	Day 4
Gpcpd1	16 33	15 93	15 17	19 29	40 96	24 34	1 736862978	0 004983755	Day 4
Dio3	6 88	3 01	2 22	1 76	0 24	1 45	0 426869623	0 005032237	Day 4
Gm42634	0 15	0	0	4 98	0	4 75	4 041269841	0 005078982	Day 4
Slc36a2	1 86	0 68	0 97	2 02	6 67	1 23	1 984639017	0 00508203	Day 4
Gm38392	4 25	1 36	2 67	6 46	5 89	4 59	1 767730496	0 005084117	Day 4
Ugt1a1	0 19	0 19	0 08	0	0	0	0 867052023	0 005088228	Day 4
Ppp1r3a	3 06	6 21	4 12	6 71	10	8 45	1 718120805	0 005137408	Day 4
Dusp5	8 58	4 65	3 71	2 99	0 65	2 22	0 444332999	0 005287477	Day 4
F730311O21Rik	0 23	0 12	0 12	0 34	0 3	0 25	1 121037464	0 005349254	Day 4
Gm49797	0 85	0 45	0 53	1 41	0 98	1 56	1 438923395	0 005357971	Day 4
Pdzd7	1 22	1 79	1 48	1 7	5 48	2 31	1 667556742	0 005414552	Day 4
Rims1	0 07	0 07	0 04	0	0	0 01	0 946540881	0 005434411	Day 4
Fgfr3-ps	0	0	0	0	0 9	0 47	1 456666667	0 005450515	Day 4
4833412C05Rik	1 31	1 61	1 09	0 46	0 12	0 17	0 534950071	0 005460523	Day 4
Car3	336 97	700 85	409 34	660 37	930 94	1118 16	1 87046257	0 005505693	Day 4
Pvalb	1537 88	2900 33	3030 04	2279 97	10516 76	6582 98	2 594306174	0 005513606	Day 4
Kcng4	1 16	1 33	6 38	3 25	27 06	6 5	3 353833193	0 005536111	Day 4
Ppp1ccb	1 03	5 4	9 64	17 25	18 95	15 56	2 871525957	0 005563448	Day 4
Myoc	7 28	13 84	25 47	33 94	48 64	29 51	2 320830813	0 005625232	Day 4
Pkia	57 69	107 52	92 53	137 36	229 38	161 14	2 036051239	0 005644842	Day 4
Svip	1 53	2 66	2	3 28	4 83	3 09	1 54515778	0 005687455	Day 4
B3galnt2	23 37	21 99	23 87	35 36	42 69	33 29	1 582998754	0 005712037	Day 4
Krt31	0 98	0 97	0 76	0 29	0	0 25	0 619964974	0 005879351	Day 4
Mybpc2	279 14	552 42	576 11	550 3	1261 44	1257 85	2 178106857	0 005893797	Day 4
Wnk2	2 08	4 15	5 8	5 76	17 77	8 19	2 310046574	0 005937165	Day 4
Gm28959	0	0	0	0 81	1 49	0 15	1 816666667	0 005945665	Day 4
Ppargc1a	1 95	2 8	3 11	3 32	3 67	3 92	1 280847145	0 00608096	Day 4
Gm5420	0 19	0 51	0 58	1 64	3 81	0 99	2 205607477	0 006090959	Day 4
mt-Rnr1	181	192 04	216 96	248 78	729 62	280 77	2 128448567	0 006107847	Day 4
Gm28437	8127 88	12987 11	10676 66	16589 7	31388 54	14469 45	1 964188629	0 006171703	Day 4
Asb14	12 45	18 76	17 21	21 63	53 75	28 43	2 077207312	0 006184636	Day 4

Ch25h	3 9	2 48	2 12	1 44	0 86	0 63	0 515652174	0 006239264	Day 4
Serpina3g	11 84	2 92	2 89	2 14	0 55	1 29	0 338014528	0 006310015	Day 4
2410018L13Rik	1 39	0 88	0 53	0 36	0 09	0 33	0 651724138	0 006315514	Day 4
St6gal2	0	1 43	0	8 8	14 84	4 2	6 961625282	0 006417551	Day 4
Lnx1	1 78	1 41	1 52	2 1	3 17	2 28	1 368352789	0 006487385	Day 4
Ar	1 42	1 96	2 08	2 53	4 79	3 15	1 592198582	0 006498375	Day 4
Trdn	37 27	68 82	32 4	76 35	50 83	103 59	1 652201569	0 006547609	Day 4
Gm14319	0 14	0 32	0 47	0 29	1 13	0 49	1 249363868	0 006579951	Day 4
Mtln	42 7	57 5	61 79	76 47	133 5	83 58	1 797381659	0 006597031	Day 4
Unc93a	0 5	0 31	0 12	0 04	0 04	0 05	0 796437659	0 006654686	Day 4
Tmem267	8 81	11 13	9 22	19 6	22 65	10 17	1 723258706	0 006772751	Day 4
BC023105	3 14	5 3	1 69	3 19	8 56	8 76	1 790555979	0 006813076	Day 4
Tlcd3b	2 04	3 52	6 13	4 84	17 04	7 38	2 196051736	0 006824739	Day 4
Plekhb1	10 22	19 84	21 22	27 1	61 02	24 77	2 135040531	0 00685677	Day 4
Gm10358	0 46	1 12	1 01	1 66	2 3	2 18	1 635062612	0 007037537	Day 4
Vwa3a	0 07	0 13	0 44	0 4	0 84	1 08	1 461538462	0 007096772	Day 4
Gm6139	0	0	0	0 29	0 23	0 46	1 326666667	0 007149634	Day 4
Tmem233	36 64	63 36	45 16	57 22	118 91	105 48	1 920963823	0 00715307	Day 4
Paqr6	0 62	0 58	1 2	1 36	3 71	1 26	1 727777778	0 00716587	Day 4
Zg16	0	0	0 11	0	2 96	0 57	2 099678457	0 00717571	Day 4
D130058E05Rik	2 45	0 72	1 04	0 58	0 07	0 53	0 579750347	0 007224116	Day 4
Gm14493	1 84	0	4 45	0	0	0	0 322927879	0 007233063	Day 4
Kcnn2	0 28	0 08	0 39	0 35	1 79	0 62	1 536	0 00724817	Day 4
Ccl24	5 67	5 89	6 61	15 13	9 22	8 23	1 680680208	0 007249708	Day 4
Rab33a	1 13	0 65	0 37	0 14	0	0 11	0 631067961	0 007255805	Day 4
2310016D23Rik	0 54	2 38	2 6	4 21	11 07	4 39	2 660798122	0 007282141	Day 4
Reg1	0	0	0	0	1 87	0 37	1 746666667	0 007285551	Day 4
Hoxd10	0 48	0 98	0 86	1 51	1 28	1 76	1 419172932	0 007380132	Day 4
Klhl4	0 88	0 55	0 69	1 84	0 98	0 76	1 28515625	0 007398321	Day 4
Gpd1	78 25	152 83	172 5	162 36	389 21	314 15	2 136652073	0 00740547	Day 4
2310016D03Rik	0 04	0 11	0 35	0 4	2 15	0 29	1 668571429	0 007440472	Day 4
Pfkfb1	3 46	6 28	10 82	9 26	35 72	12 12	2 550933786	0 007505696	Day 4
Abcd2	4 5	3 3	3 63	3 72	5 78	5 02	1 214137214	0 007536255	Day 4
Mettl21c	17 55	30 75	18 65	17 88	75 58	60 26	2 24045747	0 00755069	Day 4
Lilra5	0 06	0 13	0 14	0 37	0 34	0 34	1 216216216	0 007577886	Day 4
St3gal6	18 33	38 7	33 78	43 64	56 51	63 41	1 775503678	0 007587237	Day 4
Ugt8a	0 72	0 44	1 08	1 31	4 67	0 72	1 851145038	0 007619231	Day 4
mt-Ts1	0	0	0	0	0	0	1	0 00766353	Day 4

Ctrl	0 22	0 39	0	0 17	2 56	1 71	2 060941828	0 007684401	Day 4
Cib2	19 17	29 85	39 9	32 15	107 31	64 06	2 246736292	0 007695958	Day 4
Cadm3	5 74	6 04	8 81	12 6	13 74	8 16	1 589656634	0 007758374	Day 4
C7	1 34	1 5	3 09	2 17	9 69	3 73	2 08174692	0 007775321	Day 4
Kif24	3 54	2 46	2 37	1 68	0 71	1 18	0 577836412	0 007951738	Day 4
Amd1	48 77	44 56	45 39	29 84	226 83	92 68	2 486240474	0 007952329	Day 4
Plin4	11 12	13 69	22 12	22 79	66 3	21 7	2 278990587	0 007976281	Day 4
Prima1	0 87	0 55	0 87	0 83	5 96	1 3	2 096408318	0 007983082	Day 4
Patj	11 26	3 8	5 37	3 48	0 63	3 1	0 435766112	0 008095156	Day 4
Maob	3 79	3 43	4 83	6 29	8 98	5 38	1 571428571	0 008173346	Day 4
4932441J04Rik	0 87	0 64	0 56	0 21	0 19	0 43	0 755424063	0 008229715	Day 4
A330023F24Rik	0 62	0 72	0 72	1 28	1 52	1 08	1 359683794	0 008265929	Day 4
Klhl38	1	4 13	5 35	7 34	20 32	5 37	2 672848665	0 00828787	Day 4
Gsta3	0 22	0 42	0 12	0 43	1 53	0 24	1 382978723	0 00839411	Day 4
Lman1l	9 61	4 68	5 71	2 24	0 07	2 16	0 324782609	0 008426629	Day 4
Smim5	41 84	20 68	17 44	14 25	2 79	12 31	0 389946962	0 008434362	Day 4
Smco1	2 29	7 23	13 37	11 62	38 8	18 85	2 791425261	0 008453493	Day 4
Gm12033	35 27	62 12	60 59	68 06	117 19	103 64	1 81320661	0 008470193	Day 4
Plin1	11 81	2 54	2 16	8 47	20 81	6 49	1 987186058	0 008547787	Day 4
Phkb	46 3	64 69	58 2	67 75	88 28	110 51	1 565363842	0 008618982	Day 4
Prss46	3 7	0 62	0 75	0 36	0	0 3	0 453531599	0 008676826	Day 4
Rnase2a	0	0	0 19	4 15	0 11	0 43	2 410658307	0 008686069	Day 4
Fer1l6	0 28	0 76	0 98	0 99	4 55	1 5	2	0 008703489	Day 4
Usp2	29 86	45 39	37 03	50 07	63 4	59 85	1 529493407	0 008707608	Day 4
Gm10925	4479 79	5792 64	5364 03	7491 27	11004 2	6830 2	1 619536097	0 008746457	Day 4
Gm45051	1 08	0 42	1 69	1 26	5 89	2 87	2 103392569	0 008746459	Day 4
Pcolce2	5 17	2 76	4 24	6 41	8 68	5 69	1 567567568	0 008760535	Day 4
Lgalsl	8 14	10 43	9 93	12 75	21 54	13 51	1 612698413	0 008842669	Day 4
Mup10	3 13	0 19	0 19	2 09	4 24	3 85	2 024577573	0 008866898	Day 4
Oip5os1	23 81	28 8	24 23	33 15	45 57	36 12	1 475951904	0 0089401	Day 4
Pon1	0 86	1 54	0 23	0 75	3 94	0 95	1 534635879	0 00895228	Day 4
Has3	0 56	0 31	0 49	0 67	0 65	0 58	1 123853211	0 00912381	Day 4
Fbxo32	4 39	8 72	11 12	15 32	25 61	11 28	2 027543151	0 00915738	Day 4
Gm47155	0 9	1 24	0 79	1 03	2 62	2 38	1 522765599	0 009177257	Day 4
Dusp8	3 54	4 55	4 63	5 94	13 42	5 57	1 776717557	0 009287589	Day 4
Gm30794	0 93	2 47	2 47	2 61	7 5	4 25	1 957158963	0 009310016	Day 4
Cebpe	1 53	0 65	0 77	2 51	0 94	1 99	1 418487395	0 009372457	Day 4
Stx11	8 3	4 25	4 83	3	0 81	2 56	0 459764475	0 009477699	Day 4

Hpn	4 63	4 59	5 12	6 42	9 73	5 21	1 404844291	0 00952371	Day 4
Acyp2	17 43	43 7	34 51	45 4	106 99	54 52	2 128041363	0 009550673	Day 4
Lrrc36	0 41	0 21	0 29	0 29	1 1	0 79	1 324808184	0 009555801	Day 4
Nptx1	2 09	0 73	0 39	0 36	0 04	0 31	0 59742351	0 00962319	Day 4
Iftap	18 05	24 82	14 42	21 96	30 65	36 52	1 528114115	0 009683686	Day 4
Hsbp1l1	0 32	0 72	0 47	0 9	2 91	1	1 731707317	0 009731974	Day 4
Osbpl6	1 93	3 9	4 4	6 26	7 96	5 03	1 681783825	0 009755519	Day 4
Gjc3	0 35	0 24	0 23	0 45	0 81	0 28	1 188481675	0 009835882	Day 4
Riiad1	3 73	4 44	3	6 59	6 12	5 58	1 502470007	0 009876067	Day 4
2310040G24Rik	21 19	42 83	33 14	36 24	93 75	70 23	2 028953674	0 009901136	Day 4
Ptprr	0 36	0 53	0 47	0 84	1 65	0 62	1 401376147	0 009928586	Day 4
Gpd2	14 54	18 7	20 15	20 19	42 16	33 6	1 754743749	0 009969295	Day 4
Pagr1a	1 14	7 8	5 42	2 87	0	1 75	0 438940092	0 010005168	Day 4
Gid4	9 73	9 83	9 68	12 47	17 64	13 15	1 434863524	0 010006484	Day 4
Gm29216	3327 25	5639 6	6802 99	8327 57	20519 2	6224 68	2 223724453	0 010038253	Day 4
Chpt1	18 51	22 92	21 73	29 88	44 57	23 62	1 527660218	0 0101346	Day 4
Mirt2	2 17	0	0 18	0	0	0	0 560747664	0 010248515	Day 4
Chdh	0 22	0 09	0 18	0 33	0 7	0 23	1 220630372	0 010288835	Day 4
Mbp	40 87	19 7	80 06	78	356 05	37 35	3 302931143	0 010305842	Day 4
Cth	0 45	0 41	0 6	0 99	1 6	0 49	1 3632287	0 010339429	Day 4
Thrb	1 09	1 6	1 45	2 07	3 41	2 23	1 5	0 010360614	Day 4
Cd209b	0 39	0 58	0 29	0 46	1 15	0 92	1 298122066	0 010450296	Day 4
Ighg2c	0 94	3 61	2 11	0 92	0 28	0 26	0 461697723	0 010579075	Day 4
Tst	5 43	7 8	8 72	8 8	28	10 99	2 035671343	0 010594011	Day 4
Cadm4	5 99	3 51	8 19	8 67	31 36	5 51	2 346060899	0 010597573	Day 4
Ly6k	0 8	0 63	0 86	0 08	0 09	0	0 599243856	0 010635904	Day 4
Clps	0	0	0	0	3 2	0 35	2 183333333	0 010724446	Day 4
Slc7a10	1 54	0 71	0 55	1 95	2 24	0 96	1 405172414	0 010732282	Day 4
Zfp382	1 86	1 44	2 99	1 88	0 24	0 54	0 609257266	0 010776425	Day 4
Retn	8 27	4 96	1 35	6 66	21 55	7 49	2 201365188	0 010786589	Day 4
Dmbt1	0 15	0 06	0	0 02	0 56	0 08	1 140186916	0 011041962	Day 4
Kcnj12	8 37	10 23	11 31	11 05	19 89	17 18	1 553327256	0 011073463	Day 4
Nt5c1a	0 87	2 25	3 02	2 17	9 84	4 42	2 125820569	0 011101345	Day 4
Gria4	0 09	0 1	0 08	0 17	0 24	0 25	1 119266055	0 011190751	Day 4
Myo15b	0 75	0 45	0 46	0 2	0 09	0 22	0 753218884	0 01127383	Day 4
Ccr7	7 84	3 59	2 4	2 12	0 34	1 7	0 425430778	0 011297619	Day 4
Cmtm5	0 26	0 23	0 89	1 1	3 24	0 8	1 858447489	0 011345974	Day 4
Pxmp2	6 91	10 84	11 3	15 27	28 6	12 74	1 859906396	0 011358518	Day 4

Reg2	0 19	0 69	0	0 93	1 51	1 72	1 845360825	0 011361434	Day 4
Gm45203	0 03	0	0	0 19	0 31	0 03	1 165016502	0 011384619	Day 4
Mal	2 49	1 32	3 74	4 9	12 42	2 24	2 138388626	0 011428828	Day 4
Aqp5	0 65	0 51	0 74	0 11	0 08	0 09	0 669387755	0 011593057	Day 4
Prkaa2	7 18	11 5	12 09	13 66	19 88	17 79	1 6088244	0 011619009	Day 4
Ttll7	4 67	5 45	4 14	5 3	8 23	7 97	1 419466976	0 011629797	Day 4
Snurf	1 37	0	2 28	13 36	49 1	0	9 843609023	0 011642516	Day 4
Gpm6a	0 87	0 18	0 43	1 02	1 46	0 7	1 379464286	0 011674375	Day 4
Tmem181c-ps	0 51	0 14	0 08	0	0	0	0 804289544	0 011799641	Day 4
Zyg11a	0 11	0 05	0 08	0 19	0 35	0 24	1 166666667	0 011800326	Day 4
Tmem121	2 99	1 57	2 01	1 3	0 26	0 73	0 55276907	0 011838203	Day 4
Tmtc1	2 71	4 07	5 46	6 33	11 35	5 84	1 74015748	0 011914315	Day 4
Lsamp	0 13	0 21	0 19	0 22	0 4	0 36	1 127478754	0 011917762	Day 4
Ptprb	6 52	6 19	6 27	9 66	11 85	7 15	1 440400364	0 011940912	Day 4
Gm14698	0 54	0 71	0 39	0 73	0 62	1 9	1 346982759	0 011952255	Day 4
Hmox1	300 99	61 79	74 36	56 14	14 35	52 76	0 286840551	0 011991677	Day 4
Dact2	0 92	1 65	1 74	3 11	2 44	1 39	1 359781122	0 01208031	Day 4
Cdc6	4 43	1 85	1 56	1 26	0 43	0 74	0 500922509	0 012137802	Day 4
Gm5737	0 07	0 13	0 49	0 71	1 68	0 61	1 62601626	0 012155217	Day 4
Gm13398	1 36	0 75	0 36	0 22	0 02	0 3	0 647166362	0 012197388	Day 4
Cxcr6	1 02	0 84	0 38	0 26	0 09	0 23	0 683206107	0 012313063	Day 4
Il1a	0 57	0 2	0 44	0 11	0	0 09	0 760095012	0 012325611	Day 4
Amy2a1	0	0	0	0	8 88	2 61	4 83	0 012359013	Day 4
Hif3a	0 17	0 22	0 11	0 32	0 23	0 31	1 102857143	0 012518562	Day 4
Cpa1	0 57	0 2	0 38	0 55	2 46	0 47	1 561445783	0 012596639	Day 4
Magix	1 86	3 35	8 62	7 17	23 96	6 38	2 407011289	0 012730422	Day 4
Ppara	0 68	1 19	1 81	2 6	5 67	1 21	1 868263473	0 012764548	Day 4
1110006O24Rik	2 49	2 46	1 87	0 9	0 47	0 73	0 519348269	0 012781793	Day 4
Odf3l2	0 11	1 24	1 01	0 47	5 97	3 95	2 498134328	0 012801904	Day 4
Chaf1b	10 52	4 48	3 67	3 17	0 61	3 01	0 45177665	0 012819606	Day 4
Npc1l1	0 04	0 05	0 05	0 08	0 19	0 16	1 092356688	0 013064551	Day 4
9830004L10Rik	1 5	2	2 05	2 22	4 17	3 29	1 483040936	0 013075281	Day 4
Apoc1	13 07	5 15	2 26	8 76	17 24	13 17	1 795996593	0 013107356	Day 4
Mybl2	7 24	2 3	1 74	1 51	0 26	1 27	0 422969188	0 013168618	Day 4
Cdt1	18 21	8 05	8 22	6 11	1 62	5 16	0 423959445	0 013268169	Day 4
Gm10705	3 17	3 91	0	0	0	0	0 297619048	0 013330401	Day 4
Cldn19	1 77	0 74	2 78	4 32	12 72	1 37	2 582629674	0 013335451	Day 4
Alpl	1 17	2 3	4 39	4 07	15 81	2 93	2 376611418	0 013633266	Day 4

Bpgm	17 88	24 79	27 49	27 05	49 42	41 46	1 652952433	0 013820384	Day 4
Kcna1	0 27	0 26	0 61	0 84	1 38	0 42	1 362318841	0 013880329	Day 4
Gm31166	2 75	3 06	3 54	3 51	8 07	5 71	1 64291498	0 013901599	Day 4
Zbtb16	1 84	3 67	5 93	6 03	12 57	6 42	1 940443213	0 013933862	Day 4
Gm42984	1 33	1 52	1 28	1 97	2 84	2 03	1 380084151	0 013946516	Day 4
Ctrb1	9 02	0 94	2 01	2 52	27 53	7 29	2 694722779	0 014157452	Day 4
Prom1	0 57	0 99	0 72	1 47	1 52	0 82	1 289772727	0 014185653	Day 4
Gm47708	0 41	1 53	0 64	3 1	4 03	1 12	2 016129032	0 014189045	Day 4
Ephx2	4 07	5 87	9 78	10 32	27 72	7 52	2 137323944	0 014216447	Day 4
Cfap100	0 32	0 29	0 45	0 84	1 78	0 31	1 460591133	0 014223982	Day 4
Sh3rf2	0 31	1 56	1 03	1 96	3 57	1 69	1 73220339	0 014235385	Day 4
Dcun1d2	16 98	20 8	22 43	24 82	38 2	29 6	1 512735327	0 014242913	Day 4
6430571L13Rik	3 44	7 38	11 01	8 62	24 73	15 79	2 099879178	0 014287048	Day 4
2410137M14Rik	0	0	0	0 21	0 47	0 16	1 28	0 014313514	Day 4
Gpr37l1	0 81	0 32	0 61	1 09	2 82	0 48	1 55907173	0 014366745	Day 4
Lims2	12 72	17 77	29 05	32 93	72 65	23 16	2 106491845	0 014379502	Day 4
Clcn1	10 85	22 88	27 24	25 51	61 93	33	1 929654526	0 014499212	Day 4
Gm13652	0 07	0 13	0 11	0 01	0	0	0 909365559	0 014520321	Day 4
Car8	0 57	0 87	0 92	1 24	2 3	1 03	1 412313433	0 014810678	Day 4
Tnfrsf25	2 74	0 97	0 36	1 7	1 13	1 76	1 073550212	0 014828425	Day 4
Klhdc7a	0 27	0 15	0 13	0 32	0 88	0 14	1 222535211	0 01484941	Day 4
Aldh1l1	3 11	3 02	2 3	5 06	6 31	2 62	1 486439195	0 014883876	Day 4
Orm1	3 38	1 12	0 8	2 92	5 9	2 72	1 751807229	0 014891565	Day 4
Glb1l2	2 47	1 13	0 64	2 14	4 72	1 53	1 57320442	0 014960561	Day 4
Pcx	13 08	13 45	15 91	17 09	37 19	19	1 678697183	0 014980152	Day 4
Cyp1a1	0 07	0 08	0 2	0 33	0 79	0 14	1 271641791	0 015013398	Day 4
Phka1	22 76	46 76	49 95	46 03	92 62	87 34	1 869764024	0 015088271	Day 4
Fa2h	0 56	0 49	1 95	1 79	6 54	0 94	2 045	0 015269183	Day 4
Dpp6	1 37	2 47	1 55	1 08	0 36	0 79	0 623361144	0 015269397	Day 4
Cyp4b1	2 51	2 79	3 88	3 68	8 51	4 4	1 608374384	0 015281659	Day 4
Actn3	975 18	1423 95	1666 54	1373 11	3061 66	2743 78	1 765085396	0 015300941	Day 4
Lsmem1	5 5	9 2	5 07	8 3	10 06	11 93	1 462011419	0 015334864	Day 4
Mettl11b	1 36	3 63	3 63	4 24	8 64	5 31	1 823580034	0 015359958	Day 4
Myhas	17 57	27 41	23 65	22 95	54 97	43 57	1 737958956	0 015398148	Day 4
Sapcd2	4 82	2 63	2 49	1 56	0 42	0 87	0 452086553	0 015472912	Day 4
Gm49405	0	0	0	0 06	0 18	0	1 08	0 015533401	Day 4
Wnt7b	2 09	0 19	0 12	0 08	0 09	0 13	0 611111111	0 01553985	Day 4
Jph1	28 6	41 68	39 46	41 2	59 74	67 84	1 52368281	0 015547817	Day 4

Gal	88 37	13 95	8 48	9 69	0 6	8 77	0 193848858	0 015750742	Day 4
Gm37829	5 29	8 17	7 44	8 26	10 93	13 28	1 484100418	0 015910428	Day 4
Adnp	7 64	3 84	2 25	2 6	1 17	2 06	0 527794381	0 016006008	Day 4
mt-Atp8	13634 86	19530 77	17598 22	23221 3	30014 66	18740 42	1 417842155	0 016015427	Day 4
Gm42047	14 39	6 87	5 36	4 15	1 03	5 07	0 447332883	0 016112734	Day 4
Ky	17 63	37 62	31 57	33 25	71 62	57 95	1 846136718	0 016169702	Day 4
Asb10	7 33	17 75	22 95	21 29	57 7	23 66	2 070350774	0 01620427	Day 4
Pygm	598 54	1097 01	1412 89	1181 17	2639 05	2161 28	1 923385956	0 016280281	Day 4
Pkhd1l1	0 04	0 02	0 04	0 13	0 11	0 24	1 122580645	0 016304868	Day 4
Gpr4	2 21	1 93	2 01	2 97	4 06	2 3	1 347540984	0 016333981	Day 4
Amy2a4	0	0	0	0	8 35	1 19	4 18	0 016411255	Day 4
Gm50210	0 07	0 1	0 09	0 35	0 08	0 39	1 171779141	0 016423993	Day 4
Gm49024	0 84	1 22	1 49	1 62	6 18	2 36	2 009160305	0 016455006	Day 4
Kif1a	0 55	0 27	1 04	1 1	2 99	0 76	1 615226337	0 016474411	Day 4
Lpl	64 17	61 2	67 34	92 9	189 93	60 27	1 768432885	0 01654984	Day 4
Gm12070	25 89	41 33	46 82	45 01	78 81	69 85	1 680365687	0 016598742	Day 4
Rnase1	0 29	0	0 06	0 17	1 97	0 19	1 591044776	0 016610857	Day 4
Kcnc3	0 54	1	0 92	1 14	2 12	1 67	1 452380952	0 016629601	Day 4
Tnfrsf17	0 57	0 43	0 45	1 21	0 77	1 43	1 440449438	0 016631474	Day 4
Gm10644	0 06	0 09	0 23	0 67	0 4	0 38	1 316568047	0 016645455	Day 4
Ppp1r1a	3 59	7 12	13 71	6 67	56 59	11 27	2 827498177	0 016660014	Day 4
Me1	23 86	32 01	31 58	31 34	56 55	47 94	1 53488115	0 016758243	Day 4
A530058N18Rik	2 34	1 87	1 84	2 11	9 15	1 67	1 760220994	0 016794801	Day 4
Cmya5	32 21	57 11	60 17	66 62	95 16	84 48	1 63459899	0 016831919	Day 4
Kcns1	0 48	0 44	0 52	0 16	0 16	0 12	0 774774775	0 016896683	Day 4
Mylk2	29 84	77 89	104 93	82 27	238 35	150 52	2 198553278	0 017000254	Day 4
B4galnt3	0 32	0 36	0 32	0 56	0 61	0 49	1 165	0 017015701	Day 4
Uckl1os	4 63	13 7	14 72	15 78	39 39	18 01	2 113176144	0 017072663	Day 4
Wnt4	1 18	2 05	1 18	1 44	4 1	2 85	1 537112011	0 017111134	Day 4
Gdap1	0 51	0 59	0 64	0 52	1 68	1 34	1 379746835	0 017161163	Day 4
C2cd2	14 14	19 04	22 54	25 71	50 95	30 34	1 873297003	0 017309092	Day 4
Gm32219	2 19	2 55	3 44	4 34	5 93	2 99	1 454382826	0 017319853	Day 4
Sla2	0 7	0 27	0 19	0 08	0 08	0 08	0 778846154	0 017360974	Day 4
Mansc4	0	0	0 1	0 29	0 33	0 33	1 274193548	0 017399344	Day 4
Efna4	2 97	3 01	2 66	1 69	0 36	1 43	0 556701031	0 017448886	Day 4
Cntf	1 49	1	3 44	5 35	11 93	1 08	2 39193729	0 017460248	Day 4
Prr14l	0 5	0 38	1 97	1 6	0	0 12	0 806837607	0 017484939	Day 4
Slc10a4	0 35	0 35	0 28	0 07	0 03	0 12	0 809045226	0 017490367	Day 4

Gp2	0	0	0	0	0 45	0 06	1 17	0 01758137	Day 4
Gm21104	1 38	0 49	0 59	2 83	2 21	1 09	1 672161172	0 01768738	Day 4
Gm15833	0 15	0 48	0 52	0 37	1 96	0 83	1 484337349	0 017839691	Day 4
Gm50194	0 4	1 6	3 03	2 55	12 01	1 99	2 434620174	0 017883439	Day 4
Sbspon	0 54	0 28	0 65	0 83	1 85	0 56	1 395973154	0 018106753	Day 4
Cela2a	0	0	0	0	10 28	1 4	4 893333333	0 018128284	Day 4
Pgm1	235 98	360 82	398 65	377 81	587 35	633 85	1 60449697	0 018231972	Day 4
Gm10599	0 09	0 09	0 4	0 58	0 59	0 51	1 30726257	0 01830162	Day 4
Prxl2a	6 68	7 89	6 66	9 1	16 32	8 97	1 543128353	0 018358704	Day 4
Klhdc1	3 16	4 51	4 39	4 98	12 1	6 36	1 75564409	0 018364438	Day 4
Agl	56 38	87 47	90 67	95 07	136 25	126 58	1 519450994	0 01838046	Day 4
Tnfsf4	0 44	0 16	0 19	0 05	0	0	0 80474934	0 018407441	Day 4
Acyp1	12 66	17 13	17 34	18 49	35 89	23 29	1 609216038	0 01872359	Day 4
Ctsw	5 91	1 58	1 93	1 02	0 15	1 14	0 427536232	0 018738738	Day 4
Prkab2	26 36	38 44	32 73	32 03	64 49	57 16	1 558539739	0 01876217	Day 4
Stab2	0 6	0 87	0 63	0 89	1 15	1 44	1 270588235	0 018900039	Day 4
Gm12319	49 92	77 04	87 81	69 71	201 99	127 02	1 844698535	0 018976495	Day 4
Gm9392	1 27	1 59	2 8	3 1	4 14	4 12	1 658198614	0 019136007	Day 4
Rhbdl1	0 63	1 98	4 11	3 32	7 81	4 58	1 924897119	0 019157783	Day 4
Gm5559	24 51	43 11	36 24	36 48	72 81	65 84	1 666947408	0 019259344	Day 4
Art3	41 06	61 7	68 16	68 34	154 93	77 21	1 744940202	0 019324238	Day 4
Igkv6-25	0	0	0 61	0 3	0	18 03	5 908587258	0 019339157	Day 4
Ppm1l	6 75	11 77	14 45	15 26	31 09	19 36	1 910202947	0 019697471	Day 4
Eva1c	0 6	0 19	0 24	0 75	1 16	0 43	1 325062035	0 019844612	Day 4
Gm42417	0 15	0 11	0 96	1 1	0 4	1 89	1 514218009	0 019896274	Day 4
Tubb4a	2	1 53	2 69	2 99	4 53	2 74	1 438177874	0 019977293	Day 4
Myoz1	443 57	832 88	1016 83	884 51	1824 9	1479 12	1 825356664	0 019988441	Day 4
Tnfsf9	5 15	1 28	2 15	1 45	0 33	0 79	0 481001727	0 020022887	Day 4
Rpl3l	40 74	113 29	205 64	171 76	486 01	216 03	2 417624838	0 02008619	Day 4
Serpina3c	1 93	0 78	0 58	1 76	3 1	1 23	1 445151033	0 020104495	Day 4
Pitpnc1	5	7 6	6 08	11 84	22 52	6 5	2 023062731	0 020150335	Day 4
Pitpnm3	0 53	0 4	0 36	0 54	1 22	0 61	1 251748252	0 020218972	Day 4
Gm10221	240 36	520 62	516 41	646 5	1006 29	567 86	1 736697413	0 020232771	Day 4
Tmem45b	1 29	0 48	0 65	1 75	4 28	0 5	1 758302583	0 02025389	Day 4
Ltb	5 47	3 09	2 13	2 02	0 4	1 26	0 487947407	0 020365356	Day 4
Tmod4	138 17	290 53	260 29	310 95	410 26	396 1	1 618968482	0 020392521	Day 4
Avil	1 12	0 95	1 4	1 99	2 13	1 54	1 338485317	0 02039604	Day 4
Kif26b	4 04	1 45	1 59	1 38	0 21	0 97	0 551587302	0 020518528	Day 4

Slc30a2	1 06	1 92	2 86	2 28	8 53	2 75	1 873303167	0 020597677	Day 4
Klra7	1 14	0 9	0 25	0 18	0	0 13	0 625708885	0 020601893	Day 4
Nctc1	15 65	28 99	24 9	30 65	40 61	39 04	1 561896884	0 02067541	Day 4
Gm34256	0 56	0 58	0 31	0 73	0 86	1 02	1 260674157	0 020885524	Day 4
Ifitm1	57 46	9 43	6 9	6 68	3 54	5 28	0 240916786	0 021048539	Day 4
Mab21l3	1 22	0 53	0 73	0 41	0 02	0 29	0 678832117	0 021105588	Day 4
Tbc1d30	1	0 58	0 59	0 14	0 22	0 5	0 746615087	0 021175233	Day 4
Kl	0 04	0 01	0 07	0 11	0 17	0 11	1 086538462	0 021294406	Day 4
Camk2a	42 75	60 08	60 95	58 44	101 19	93 34	1 534776352	0 02133594	Day 4
Col4a4	0 19	0 2	0 38	0 32	0 87	0 39	1 214854111	0 021436047	Day 4
Gm10184	0	0	0	0 21	0	0 32	1 176666667	0 021446912	Day 4
Fam131a	12 79	17 57	20 36	17 62	50	22 36	1 730826508	0 021515223	Day 4
Dhrs7c	185 84	202 83	196 28	204 24	309 23	284 16	1 36173144	0 021574195	Day 4
Epm2a	6 67	10 95	13 81	13 21	25 02	16 54	1 677897183	0 021658653	Day 4
Clec4d	32 83	5 52	5 02	4 4	0 51	5 07	0 279922364	0 021744649	Day 4
Kcnj15	6 49	4 4	1 63	2 47	0 24	1 68	0 476159794	0 021831498	Day 4
Zfp683	0 09	0 24	0 21	0	0	0	0 847457627	0 021964954	Day 4
Tcea3	31 5	79 32	95 25	88 48	221 53	107 93	2 013392644	0 022004439	Day 4
Lrrn3	0 09	0 07	0 13	0 23	0 31	0 15	1 121580547	0 022045564	Day 4
Pfn2	23 52	41 78	40 13	49 06	64 36	53 05	1 562943835	0 022059159	Day 4
Gldn	0 6	0 5	0 69	1 06	1 63	0 55	1 302713987	0 022090057	Day 4
Gm8424	2 29	7 17	7 76	8 62	15 58	9 94	1 836795252	0 02219763	Day 4
Svs4	0 22	0 1	0	0	3 32	0 2	1 963855422	0 02221121	Day 4
Atp2a1	2391 4	4239 03	5115 67	4057 01	8065 52	7161 59	1 64158276	0 022247388	Day 4
Me3	6 03	18 51	28 05	22 62	62 55	26 4	2 060982191	0 022281436	Day 4
Pla2g5	0 58	0 4	0 89	0 66	2 52	0 41	1 353182752	0 022356471	Day 4
Gm37035	0 05	0 05	0 05	0 29	0 32	0 05	1 161904762	0 022391097	Day 4
Gm33432	0 63	1 6	3 34	2 41	8 72	2 99	1 997666278	0 022413271	Day 4
Gm16759	0 34	0 4	0 34	0 17	0 06	0 1	0 816176471	0 022674827	Day 4
4632428C04Rik	0 17	0 4	0 45	0 61	1 46	0 51	1 388059701	0 02271173	Day 4
Dusp15	0 84	0 59	1 42	2 41	7 65	0 58	2 331623932	0 022849641	Day 4
Gm26781	0 66	0 2	0 44	0 07	0	0 12	0 741860465	0 022985333	Day 4
Prx	5 34	2 92	10 31	9 2	33 36	4 1	2 302271674	0 023057598	Day 4
Nkain4	0 18	0 72	0 42	1 59	2 31	1 48	1 939814815	0 023079804	Day 4
0610040J01Rik	2 99	1 7	2 8	3 39	6 49	3 08	1 521448999	0 023156027	Day 4
Sohlh2	1 77	0 99	0 9	0 67	0 05	0 39	0 617117117	0 023312526	Day 4
Catsper4	0	0 16	0 09	0 58	0 23	0 23	1 243076923	0 023396893	Day 4
Hpse2	9 03	9 64	11 04	12 54	21 79	11 48	1 492204219	0 023439419	Day 4

Bbox1	0 48	0 3	0 17	0 04	0 08	0 04	0 8	0 023507573	Day 4
Lingo3	1 7	3 22	5 31	4 46	8 57	6 8	1 725623583	0 023718048	Day 4
Dnajc28	1 96	3 11	3 08	3 49	5 28	4 35	1 44573991	0 023791404	Day 4
Tnfsf14	4 28	1 12	0 82	0 89	0 18	0 57	0 503253796	0 023921174	Day 4
Pptc7	9 85	13 8	21 88	22 46	33 51	21 01	1 648052751	0 023924039	Day 4
Nlrp3	2 84	1 3	0 86	0 94	0 12	0 54	0 575	0 023951512	Day 4
Cd59b	1 95	3 41	5 78	6 47	11 33	6 97	1 963932107	0 023977854	Day 4
Gm47898	0 25	1 14	0 84	1 17	2 6	1 24	1 531548757	0 024023959	Day 4
Ccdc85c	2 86	3 37	5 45	3 94	14 03	5 15	1 779291553	0 024197932	Day 4
Lrp2bp	0 65	1 17	1 21	1 26	1 8	2 33	1 391376451	0 024246901	Day 4
Cdh19	0 36	0 18	0 13	0 61	0 35	0 26	1 14986376	0 024371429	Day 4
Gzma	13 31	5 71	5 97	4 42	0 76	3 7	0 424437299	0 024399562	Day 4
Hepacam	0 16	0 14	0 22	0 35	0 87	0 14	1 238636364	0 024456271	Day 4
Adcy8	0 04	0 04	0 03	0 07	0 21	0 02	1 061093248	0 02453945	Day 4
Mylf-ps	4 55	5 63	3 33	4 75	10 36	5 98	1 459115687	0 024567691	Day 4
Try4	0	0	0	0	10 49	2 5	5 33	0 024583172	Day 4
Gdf10	7 19	2 5	6 64	9 7	8 85	6 48	1 4500776	0 024646235	Day 4
Gm14703	0 14	0 14	0 23	0 23	1 1	0 24	1 301994302	0 024818223	Day 4
Gm20560	0 78	0 26	0 17	0 15	0 05	0	0 760095012	0 024824628	Day 4
Gys2	0	0	0	0 05	0 12	0 02	1 063333333	0 024851453	Day 4
Ddn	0 43	0 14	1 1	0 83	3 07	0 75	1 638115632	0 024928978	Day 4
Fanca	8 35	2 15	5 14	3 13	0 25	2 92	0 498927039	0 024969083	Day 4
Asb11	10 15	26 46	42 81	45 19	69 26	44 92	1 970031546	0 024979362	Day 4
Foxd3	0 68	0 48	0 63	0 87	1 49	0 75	1 275574113	0 024992957	Day 4
Gm12199	0 68	0 9	0 91	0 81	5 89	2 94	2 302367942	0 025010662	Day 4
2210408F21Rik	3 75	13 86	17 06	16 58	53 41	16	2 362357314	0 025111168	Day 4
Dtnbos	0 05	0 04	0	0 12	0 17	0 19	1 126213592	0 025168153	Day 4
Slc37a4	28 4	33 21	33 2	29 73	59 9	58 44	1 5445251	0 025237051	Day 4
Wfdc1	18 4	39 61	43 79	39 26	96 59	55 96	1 858874046	0 02523878	Day 4
Foxf1	0 95	0 64	0 62	0 36	0 25	0 22	0 73512476	0 025254587	Day 4
Gkn3	1 25	1 7	0 56	2 82	2 96	1 34	1 55453149	0 02531212	Day 4
Rorc	2 08	8 12	13 8	13 02	38 16	10 08	2 38	0 025312247	Day 4
Rnf39	0 08	0 15	0 22	0 32	0 22	0 29	1 110144928	0 025440053	Day 4
Lypd2	1 22	0 91	2 22	2 22	8 7	1 77	2 134693878	0 025468813	Day 4
Bmp7	0 24	0 15	0 27	0 44	0 95	0 23	1 262295082	0 025480709	Day 4
Has2	5 46	2 12	1 49	1 76	0 22	0 94	0 490472245	0 025486043	Day 4
Asb2	76 2	109 45	148 92	125 19	284 76	168 35	1 722013212	0 025522036	Day 4
Slc6a19	0 55	1 16	1 15	0 49	0 01	0 26	0 641638225	0 025555387	Day 4

Crmp1	1 31	1 22	1 1	0 43	0 55	0 15	0 622926094	0 025617756	Day 4
Scn4b	6 03	19 99	40 34	29 31	112 84	32 05	2 554786621	0 025717863	Day 4
Pfkfb3	35 49	23 07	15 75	26 93	57 07	29 43	1 506014746	0 025870321	Day 4
Gm37403	0 15	0 23	0 99	1 26	2 9	0 6	1 775743707	0 025933513	Day 4
Abhd6	2 15	1 76	2 5	2 83	4 04	2 62	1 327311371	0 025989433	Day 4
Gm2663	0	0	0	0	1 02	0 13	1 383333333	0 026124021	Day 4
Gm44117	0	0 03	0 06	0 09	0 39	0 13	1 16828479	0 026226653	Day 4
Gm36235	0 77	1 58	1 57	1 24	2 58	3 36	1 471098266	0 026276036	Day 4
P2rx1	2 13	1 09	1 57	1 98	3 64	1 98	1 36071887	0 026301743	Day 4
Ubxn2a	5 67	8 94	7 14	7 95	14 11	10 51	1 437171717	0 026326543	Day 4
Gm5860	0 46	1 67	2 31	2 58	7 53	2 64	2 116935484	0 026400256	Day 4
Col4a3	0 06	0 14	0 41	0 16	0 62	0 23	1 110803324	0 026600708	Day 4
Gm48420	0 11	0	0	1 8	0 81	0	1 803858521	0 026767845	Day 4
Egln1	25 82	34 31	40 24	43 08	78 72	39 79	1 592241463	0 026795578	Day 4
Spp1	1312 05	196 62	94 22	205 99	17 4	112 2	0 210842586	0 026798777	Day 4
Sycn	0	0	0	0	1 41	0 76	1 723333333	0 026887189	Day 4
Map2k6	5 62	7 22	7 66	7 47	12 24	10 23	1 401702128	0 027013449	Day 4
Art4	0 41	0 13	0 45	0 82	0 87	0 38	1 270676692	0 027034742	Day 4
Mmrn1	0 98	0 15	1 35	3 35	1 14	1 54	1 647810219	0 027057282	Day 4
Ntrk3	0 3	0 3	0 56	0 33	1 42	0 28	1 209134615	0 027152482	Day 4
AA474408	0 11	0 16	0 04	0 27	0 29	0 17	1 126888218	0 02722922	Day 4
Fcrla	1 68	1 04	1 98	0 65	0 07	0 44	0 54025974	0 027271469	Day 4
Gm10334	0	0	0	0	0 63	0 48	1 37	0 027294028	Day 4
Ncmap	3 07	1 18	2 46	3 98	13 28	1 16	2 205973223	0 027513439	Day 4
Gm12537	48 26	63 06	75 61	68 4	114 58	98 43	1 497446428	0 027567863	Day 4
Gm15179	2 5	7 12	14 06	11 25	35 25	12 93	2 339955022	0 02763555	Day 4
Aldh4a1	14 82	23 44	24 73	24 1	48 96	30 3	1 611759357	0 027665296	Day 4
Kifc1	17 47	7 15	6 36	6 29	1 44	4 21	0 439670394	0 027738062	Day 4
Syt14	0 44	0 37	0 11	0 12	0 03	0 09	0 826530612	0 027849661	Day 4
Gabrq	0	0	0 01	0 03	0 15	0 01	1 059800664	0 027868965	Day 4
Gm47512	0	0	0	0 38	0 18	0	1 186666667	0 027872479	Day 4
Snai1	36 58	13 45	16 95	14 51	2 64	8 92	0 415404401	0 027876001	Day 4
Mpc1	154 92	269 8	218 75	280 56	496 63	258 12	1 606122481	0 027921482	Day 4
Atp2b3	0 81	1 53	1 87	1 58	3 58	2 23	1 441054092	0 027961415	Day 4
Smox	53 66	62 61	113 73	52 12	327 9	112 55	2 126909871	0 028116057	Day 4
4833422M21Rik	0 25	0 29	0 1	0	0 02	0 05	0 843406593	0 028137048	Day 4
Fbxl2	9 45	5 2	4 78	4	0 75	3 04	0 481052162	0 028429965	Day 4
Txlnb	27 14	40 35	40 65	42 6	75 71	48 38	1 526813029	0 028511586	Day 4

Gvin-ps1	0 04	0 08	0 04	0 01	0	0 01	0 955696203	0 028516393	Day 4
Plk5	0 03	0 05	0 21	0 3	1 24	0 05	1 395136778	0 028544514	Day 4
Mthfd1l	8 58	2 39	2 38	2 58	0 55	1 46	0 464220183	0 028641308	Day 4
St8sia2	11 26	2 81	4 45	2 94	0 45	2 82	0 427973978	0 028744367	Day 4
Akr1c18	0 17	0 06	0	0 23	0 48	0 31	1 244582043	0 028781668	Day 4
Gm10549	0 6	0 25	0 76	0 88	2 69	0 78	1 594360087	0 028843416	Day 4
Nxpe5	25 6	8 04	5 41	6 99	0 86	4 33	0 360998811	0 028882712	Day 4
Wnt6	1 19	0 44	0 85	1 33	1 27	0 91	1 187956204	0 02888284	Day 4
Pmp22	125 01	98 32	156 31	160 07	376 42	102 76	1 678470625	0 02892579	Day 4
Tubb3	28 1	7 06	8 04	8 15	0 94	4 23	0 353246753	0 028982847	Day 4
Ush1c	0 09	0 18	0 28	0 35	0 46	0 77	1 290140845	0 029203009	Day 4
Il18rap	2 95	0 74	0 65	0 64	0 2	0 74	0 623978202	0 029207842	Day 4
Gzmc	0 75	1 11	0 52	0 22	0	0 22	0 639405204	0 0292549	Day 4
Dgkg	0 18	0 11	0 31	0 44	0 31	0 4	1 152777778	0 029360277	Day 4
Tmem52	7 45	32 94	60 4	45 8	149 51	54 73	2 437999807	0 029372716	Day 4
Gm15510	1 06	2 42	2 25	2 37	6 37	3 13	1 703321879	0 029417424	Day 4
Adipoq	47 04	12 7	10 29	36 41	58 99	24 21	1 678899083	0 029464901	Day 4
Gm42940	0 4	0 69	0 29	0 71	0 65	1	1 223744292	0 0295113	Day 4
Cxcl2	9 37	0 32	0 55	0 6	0	0 24	0 290030211	0 029578383	Day 4
Lrrc14b	2 92	10 78	16 74	10 66	43 96	18 81	2 285586124	0 029714642	Day 4
Sox10	1 94	0 83	1 99	2 21	6 13	1 33	1 632731959	0 029750552	Day 4
Dnaaf3	2 83	1 58	1 28	0 83	0 18	1 06	0 583429229	0 029778626	Day 4
Gm8956	0 18	0 11	0	0 27	0 31	0 84	1 343465046	0 029800822	Day 4
Ccdc85a	0 63	0 94	1 03	1 61	2 11	1 13	1 401785714	0 029828746	Day 4
2310014F06Rik	1 16	1 81	2 26	2	6 03	2 15	1 60145808	0 03001396	Day 4
Gm14261	0 39	0 46	0 68	0 58	1 86	0 93	1 406181015	0 030200435	Day 4
Spaar	0 31	0 35	0 35	0 34	1 08	0 44	1 211970075	0 030271352	Day 4
Igfals	1 06	0 84	1 32	1 26	4 33	1 31	1 591639871	0 03031561	Day 4
Gm45021	0 2	1 04	0 67	2 21	1 77	2 69	1 969450102	0 030343011	Day 4
B230322F03Rik	0 11	0 1	0 23	0 41	0 68	0 24	1 25872093	0 030347831	Day 4
Synm	15 23	32 72	31 63	31	54 16	48	1 648825381	0 030538599	Day 4
S100b	2 68	2 41	4 24	5 23	9 39	2 69	1 647201946	0 030643873	Day 4
Asrgl1	5 08	7 32	7 69	7 52	14 42	9 65	1 498051104	0 030739914	Day 4
Slc6a20b	0 04	0 04	0 02	0	0	0	0 967741935	0 030843609	Day 4
Pde4a	5 92	7 78	12 71	10 2	29 42	10 71	1 8133288	0 030876332	Day 4
Trp53cor1	3 4	1 99	1 68	1 41	0 22	1 38	0 596822244	0 030892152	Day 4
Fosb	1 15	4 7	2 46	1 41	0 99	0 7	0 539345712	0 030908288	Day 4
Acsm3	0 16	0	0 12	0 09	1 19	0 16	1 353658537	0 030921837	Day 4

Gsn	424 02	423 74	614 37	566 96	1079 13	595 92	1 532294063	0 031000733	Day 4
Tgif1-ps	0	0 46	0 41	1 9	2 47	0 69	2 082687339	0 031002209	Day 4
4933411E06Rik	0 11	0 16	0 16	0	0	0	0 874635569	0 031081843	Day 4
Rtn2	271 8	426 68	480 08	406 3	815 72	645 4	1 583008903	0 031403514	Day 4
Gm28438	2067 66	3316 99	4537 62	3901 09	9644 98	3878 89	1 755917975	0 031406623	Day 4
Ckm	3162 62	6485 26	7886 77	5819 96	16149 27	10861 37	1 872177857	0 031436294	Day 4
Gm15417	9 55	18 43	16 3	16 01	32 86	21 48	1 551395939	0 031543559	Day 4
Qrfp	0 97	0 17	0 06	0 08	0	0 07	0 75	0 031696174	Day 4
Ppp1r3b	4 72	1 47	0 99	1 21	0 35	0 85	0 531434185	0 031866849	Day 4
Gm12689	0	0 09	0 08	0 43	0 13	0 3	1 217665615	0 032075322	Day 4
Mpc2	71 27	129 37	137 59	150 45	273 58	137 06	1 653107874	0 032131347	Day 4
-	0	0 79	0	4 07	16 42	14 36	9 986807388	0 032158589	Day 4
Gm32282	0 38	0 87	1 05	1 21	3 43	1 85	1 790566038	0 032234541	Day 4
Pkdcc	31 63	39 63	53 65	50 78	104 86	52 77	1 652802752	0 032282728	Day 4
Smtnl2	25 47	54 55	78 65	62 41	164 27	79 47	1 912228614	0 032399251	Day 4
Cfi	1 36	0 16	0 31	0 12	0	0 05	0 6563147	0 032430828	Day 4
Trim63	59 97	85 8	116 71	113 38	169 7	123 83	1 544033449	0 032446772	Day 4
Creg2	0 58	0 19	0 07	0 09	0 03	0 06	0 828125	0 032517647	Day 4
Gm11518	300 4	544 15	144 93	67 02	139 44	60 05	0 271552072	0 032543108	Day 4
Sez6	0 03	0 05	0 07	0 17	0 71	0 09	1 26031746	0 032551061	Day 4
Rrm2	48 35	15 3	11 36	12 84	2 14	9 82	0 356364569	0 032577911	Day 4
Pramel13	0 61	0 45	0 5	0 21	0 09	0 2	0 76754386	0 032646588	Day 4
3110045C21Rik	0 07	0 13	0 24	0 39	0 44	0 53	1 26744186	0 032728286	Day 4
Rhpn2	0 25	0 6	0 47	0 79	0 97	0 68	1 259259259	0 032728571	Day 4
Rasd2	1 12	3 37	2 74	3 28	3 49	5 83	1 524926686	0 032752267	Day 4
Gm48882	0 2	0 06	0 4	0 43	1 76	0 3	1 5	0 032829221	Day 4
Gm28374	0 13	0	0	0 2	0 51	0 36	1 300319489	0 032893814	Day 4
Slc6a12	0 27	0 25	0 15	0 79	0 82	0 18	1 305177112	0 033032066	Day 4
1810009J06Rik	0	0	0	0	0 78	0 25	1 343333333	0 03309592	Day 4
Amd2	0 21	0 2	0 24	0 17	0 98	0 37	1 238356164	0 033098551	Day 4
Gm48128	0	0	0 11	0 4	0 18	0 11	1 186495177	0 03320165	Day 4
Gstk1	11 19	16 79	21 18	20 5	46 46	19 04	1 706288344	0 033240813	Day 4
L2hgdh	4 2	5 29	5 25	5 15	10 02	6 72	1 403043968	0 033341376	Day 4
Gm38431	1 76	1 18	2 26	0 49	0 5	1 01	0 609756098	0 033404105	Day 4
Rps2-ps3	0 5	0 4	0 79	1	1 9	0 84	1 437100213	0 0334728	Day 4
Emid1	3 26	3 71	5 79	5 2	11 04	5 89	1 594543147	0 033550689	Day 4
Nek2	7 07	3	3 28	2 78	0 44	1 67	0 482568807	0 033567502	Day 4
Ctnnd2	0 8	0 64	0 24	0 52	0 14	0 45	0 878205128	0 033703703	Day 4

Gm43778	0	8 7	2 89	12 93	39 33	6 76	4 250856751	0 033705764	Day 4
Slc6a1	0 22	0 04	0 1	0 11	0 34	0 02	1 032738095	0 033718248	Day 4
Rpsa-ps10	0	0	0	0 14	0 13	0 35	1 206666667	0 033784931	Day 4
A430110C17Rik	0	0	0	0 03	0 01	0 07	1 036666667	0 033845067	Day 4
Entpd4	22 26	32 58	45 62	41 55	73 45	48 69	1 611154069	0 033897006	Day 4
Csmd1	0 08	0 05	0 17	0 16	0 12	0 14	1 036363636	0 033947647	Day 4
Gcsam	0 72	0 34	0 72	0 32	0 12	0 21	0 763598326	0 03415941	Day 4
Pla2g4e	1 85	5 1	3 96	4 41	7 77	6 54	1 561466571	0 034172467	Day 4
4930590J08Rik	0 14	0 06	0 2	0 21	0 38	0 52	1 208823529	0 034294896	Day 4
Akr1c14	0 9	0 43	0 19	0 41	0 83	0 59	1 068584071	0 03433516	Day 4
Nt5dc3	8 35	10 78	14 69	12 9	21 64	16 44	1 466051059	0 034466999	Day 4
Kcnc4	2 55	8 74	9 53	7 41	18 2	16 01	1 873215785	0 0345753	Day 4
Ocstamp	1 63	0 37	0 29	0 28	0 09	0 24	0 68241966	0 034576813	Day 4
AA467197	18 68	4 62	4 02	5 09	0 55	2 1	0 354221636	0 034581396	Day 4
Egflam	4 93	5 38	6 73	6 5	24 96	9 95	2 216067864	0 034657966	Day 4
Gm15756	1 04	2 46	3 6	1 99	7 16	5 89	1 786138614	0 034709375	Day 4
Rps2-ps13	2 43	1 55	1 44	0 51	0	0 84	0 516627078	0 034737641	Day 4
Ankrd23	284 83	663 31	799 13	667 7	1668 04	869 26	1 832860073	0 034754844	Day 4
Ptprn	11 37	1 18	1 52	1 65	0 13	1 27	0 354422964	0 034866343	Day 4
Myh11	13 04	9 4	12 83	14 94	35 1	9 35	1 630258688	0 034892159	Day 4
Eepd1	16 69	16 57	19 01	18 29	41 81	19 61	1 496471865	0 034913171	Day 4
Gm38560	1 74	3 22	4 3	3 3	9 73	4 78	1 697389886	0 034967217	Day 4
Nr4a1	31 43	67 01	75 05	64 36	133 88	84 41	1 618505298	0 034989454	Day 4
Nat8l	0 53	0 33	0 32	0 55	0 75	0 4	1 124401914	0 035028843	Day 4
Adssl1	302 11	509 71	610 13	575 23	926 65	716 04	1 558595038	0 035119451	Day 4
Padi2	13 25	17 27	38 59	34 86	67 39	26 12	1 821800028	0 035176806	Day 4
Adh1	9 9	14 44	13 57	13 63	26 79	17 63	1 492300171	0 035186462	Day 4
Fxyd7	0 24	0 39	0 38	0 38	2 5	0 55	1 603491272	0 035285742	Day 4
Gm16062	4 14	6 36	14 42	12 55	23 53	12 72	1 85530086	0 035300743	Day 4
Asb18	1 58	6 04	6 73	6 12	13 27	7 12	1 700864553	0 035322148	Day 4
A430073D23Rik	0 53	0 7	0 2	0	0	0	0 677200903	0 035488191	Day 4
Slc7a4	1 7	1 56	2 23	0 85	0 08	0 59	0 532391048	0 035597462	Day 4
Chrm2	0	0 01	0	0 06	0 02	0 03	1 033222591	0 035671271	Day 4
Asb15	3 01	4 65	5 07	4 57	8 93	6 22	1 444373808	0 035671796	Day 4
Spock3	0 02	0 03	0 11	0 13	0 3	0 12	1 123417722	0 035767646	Day 4
Pllp	2 93	1 31	5 73	3 54	11 12	1 06	1 443330763	0 035894249	Day 4
Gm20692	0 75	0 57	0 39	0 1	0	0	0 658174098	0 035907003	Day 4
Gm16271	0 15	0	0 34	0 8	1 05	2 2	2 020057307	0 036085257	Day 4

Gm42892	0 03	0 05	0 03	0 13	0 11	0 09	1 07073955	0 036107227	Day 4
Mrap	3	1 06	0 95	2 37	6 78	1 24	1 671660424	0 036155162	Day 4
Try5	0	0	0	0	7 06	1 47	3 843333333	0 036255549	Day 4
Map3k21	0 07	0 07	0 14	0 03	0 01	0 02	0 932926829	0 036357427	Day 4
Prf1	1 24	0 49	0 69	0 5	0 14	0 19	0 706642066	0 036502438	Day 4
Angptl7	3 21	6 83	23 41	13 06	49 36	20 71	2 362962963	0 03651631	Day 4
Adk	31	27 52	37 45	32 61	59 56	35 52	1 320501162	0 036608341	Day 4
2010204K13Rik	7 06	6 04	2 3	2 53	1 15	1 98	0 470652174	0 036705797	Day 4
Gm20300	0 62	1	0 32	0 49	1 47	1 71	1 350202429	0 036720111	Day 4
Tmem143	14 78	22 43	26 46	25 6	48 16	27 61	1 565471726	0 037037056	Day 4
Map3k19	0 8	0 31	0 42	0 22	0 01	0 23	0 763796909	0 03704576	Day 4
Efcab6	1 21	1 09	1 22	1 22	5 77	2 33	1 889570552	0 037177999	Day 4
Gm13340	0 28	0 44	0 44	0 58	1 01	0 66	1 262019231	0 037240585	Day 4
Pgap6	21 76	7 09	4 24	5 97	1 56	4 1	0 40537545	0 037404671	Day 4
Cnga3	0 03	0 15	0 31	0 14	0 49	0 26	1 114613181	0 037412753	Day 4
Vldlr	4 72	10 52	11 01	13 48	19 57	10 08	1 577094017	0 037479489	Day 4
Gm49701	0 09	0 14	0 3	0 42	0 75	0 71	1 382436261	0 037652736	Day 4
Disp2	0 67	0 31	0 35	0 24	0 05	0 21	0 808314088	0 037675676	Day 4
E330011O21Rik	0 4	0 78	0 56	1 25	2	1 9	1 719409283	0 037852175	Day 4
Lilr4b	50 66	15 98	7 77	10 82	1 39	7 25	0 290143392	0 03789401	Day 4
Ppp2r2c	0 07	0 1	0 1	0 23	0 22	0 13	1 094801223	0 037967207	Day 4
Aldh1a1	7 3	10 99	15 42	12 74	26 01	16	1 573140834	0 03804517	Day 4
Folr1	5 94	2 29	3	1 89	1 9	3 92	0 752635278	0 038203644	Day 4
Zan	0 28	0 21	0 31	0 1	0 1	0 11	0 871052632	0 038364507	Day 4
Ahrr	0 34	0 32	0 24	0 22	0 44	0 49	1 064102564	0 038369442	Day 4
Cdh2	19 81	7 19	6 61	6 5	1 49	5 2	0 442228899	0 038401634	Day 4
Prdm16os	0 12	0 09	0 15	0 16	0 57	0 15	1 154761905	0 038441026	Day 4
Adrb3	0 48	0 13	0 14	0 69	1 13	0 16	1 328	0 038683882	Day 4
Mcm10	6 64	2 95	1 69	2 56	0 38	1 57	0 525910364	0 03871326	Day 4
Sapcd1	0 8	0 72	0 54	0 35	0	0 23	0 707509881	0 038815309	Day 4
Sfmbt2	0 27	0 46	0 16	0 09	0	0 11	0 822622108	0 039016836	Day 4
Gm37855	0 11	0 13	0 06	0 03	0	0 01	0 921212121	0 039072756	Day 4
Map7d3	0 51	0 66	0 45	0 31	0 09	0 32	0 805194805	0 039392956	Day 4
Apol8	0 88	0 52	0 13	0 25	0 04	0 06	0 739514349	0 039404822	Day 4
Fam240a	0 47	0 93	1 21	2 7	5 73	0 37	2 103386809	0 039429209	Day 4
Colec11	1 48	0 58	1 33	1 8	2 04	1 49	1 303599374	0 039521871	Day 4
Prr33	21 88	35 42	45 39	41 38	89 74	48 57	1 728545747	0 039571434	Day 4
5430401H09Rik	0 07	0 55	0 52	1 08	3 1	0 12	1 763285024	0 03959689	Day 4

Gm8113	0 74	0 39	0 16	0 14	0 08	0 12	0 778554779	0 039660845	Day 4
Tnnt3	5534 81	10788 34	11042 43	8984 63	20039 69	15830 06	1 639010135	0 039801516	Day 4
Slc1a1	0 21	0 34	0 83	0 68	1 54	0 47	1 299086758	0 039802365	Day 4
B430119L08Rik	0 12	0 06	0 02	0 05	0 34	0 33	1 1625	0 039925863	Day 4
Cox8b	364 55	923 81	1387 77	1093 12	3235 28	985 28	1 984480036	0 039963679	Day 4
Gm44732	0 68	1 88	0	0	0	0	0 539568345	0 04004207	Day 4
Clvs1	0 9	2 3	0 83	3 25	0 34	7 76	2 041251778	0 040113343	Day 4
Gpr165	0 02	0 09	0 06	0 07	0 15	0 15	1 063091483	0 040200436	Day 4
Slc25a4	758 62	1424 2	1354 79	1333 26	2547 21	1601 86	1 549261285	0 040242608	Day 4
Ccne1	10 01	4 14	2 54	2 98	0 46	2 84	0 471305231	0 040254961	Day 4
G730003C15Rik	0 39	0 22	0 21	0 4	0 54	0 44	1 146596859	0 040328184	Day 4
Rps12-ps4	4 39	2 03	0	0	0	0	0 318471338	0 040383858	Day 4
Gm29094	0 49	0 5	0 3	0	0	0	0 699300699	0 040528376	Day 4
Ndufc1	233 51	323	362 21	340 67	579 54	392 53	1 427483401	0 040550174	Day 4
Timp1	246 94	123 14	127 86	115 9	14 95	68 94	0 40481894	0 040582834	Day 4
-	0 72	0 22	0 15	0	0	0 08	0 753056235	0 040607113	Day 4
Gm11149	0 44	0 31	0 3	0 04	0	0 1	0 775308642	0 040626917	Day 4
Gm21955	0 55	0 22	0 32	0 48	0 77	0 44	1 146699267	0 040713454	Day 4
Tdrd6	0 01	0 01	0 01	0 2	0 47	0 02	1 217821782	0 040736274	Day 4
mt-Nd3	640 46	1210 31	1566 27	1236 45	3953 13	1121 9	1 846317587	0 041078873	Day 4
Isca1	68 41	104 27	108 64	110 17	190 96	116 11	1 478052898	0 041083879	Day 4
Pf4	577 12	136 47	89 51	116 82	52 54	91 04	0 326758467	0 041188457	Day 4
Hr	2 85	4 38	12 65	9 2	22 42	9 86	1 944055944	0 041211585	Day 4
Ltb4r2	0 38	0 27	0 34	0 1	0 07	0 24	0 854636591	0 041229534	Day 4
Gadd45g	93 4	58 29	44 63	56 2	169 54	72 27	1 510184628	0 041361323	Day 4
Dnajc27	6 24	8 77	13 16	11 64	24 09	11 42	1 608918832	0 041552423	Day 4
Plet1os	0 1	1 98	1 31	1 99	4 97	2 59	1 96400626	0 041630397	Day 4
Gm36356	0 05	0 02	0 04	0 13	0 36	0 02	1 128617363	0 041704895	Day 4
Aldoa	4703 16	8208 86	11749 15	9154 61	17147 04	13939 67	1 63169164	0 04170701	Day 4
Lrrc20	20 03	36 47	45 47	33 64	93 43	48 45	1 700676384	0 041872951	Day 4
4930525G20Rik	0 04	0	0 02	0 03	0 16	0 09	1 071895425	0 041922623	Day 4
Lgi1	0 6	1	0 22	1 43	2 39	0 24	1 46473029	0 042195898	Day 4
B230312C02Rik	0 74	1 39	1 26	1 05	2 71	2 42	1 436619718	0 042237065	Day 4
Ccr3	1 15	0 82	0 41	1 67	0 47	1 73	1 276951673	0 042264271	Day 4
Gm8543	0	0 08	0 09	0 24	0 11	0 6	1 246056782	0 042313448	Day 4
Gm16184	2 16	2 25	1 17	0 78	0	0 44	0 491841492	0 042551822	Day 4
Tpm1	5070 28	10042 86	9655 38	7699 39	17842 25	14567 71	1 619293043	0 042595672	Day 4
Ak1	319 55	581 16	587 83	526 07	972 31	769 17	1 522285691	0 042616258	Day 4

Ccr1	18 53	5 36	3 01	4 92	0 57	2 71	0 37458194	0 042627925	Day 4
Timd4	0 34	0 18	0 58	0 58	1 32	0 45	1 304878049	0 042687299	Day 4
Slc25a42	5 42	8 21	11 7	10 9	18 8	10 69	1 531591952	0 042732998	Day 4
Gpr88	0 59	1 13	0 17	0 3	0 02	0 25	0 73006135	0 042756266	Day 4
Ptk2b	27 83	12 2	9 75	9 84	2 18	8 74	0 450170519	0 042800819	Day 4
Ccl22	3 81	2 88	2 02	2	0 17	1 06	0 532023911	0 042969054	Day 4
Dnd1	0 92	0 92	0 37	0 13	0 21	0 16	0 671785029	0 04300711	Day 4
A730020M07Ril	0 23	0 24	0 14	0 18	0 24	0 34	1 041551247	0 043088755	Day 4
Cd209g	1 91	2 07	2	2 13	4 41	2 68	1 360801782	0 043229246	Day 4
Fabp5	614 78	156 26	126 06	154 9	43 58	118 18	0 355138318	0 043315008	Day 4
Gm13283	0 76	0 09	0 42	0 08	0 04	0 09	0 75175644	0 043368151	Day 4
Mreg	3 71	7 97	9 98	9 04	20 66	8 69	1 678426602	0 043392056	Day 4
Metrn	27 13	20 17	26 57	22 08	58 97	24 92	1 417588136	0 043393932	Day 4
Adamts4	18 42	12 65	16 75	12 47	1 29	5 8	0 443919717	0 043418485	Day 4
Plaat1	2 84	13 57	10 18	11 97	21 09	12 87	1 653599189	0 043436776	Day 4
3230119M05Ril	0	0 07	0 06	0 06	0 48	0 39	1 255591054	0 0434779	Day 4
Tcf15	5 1	8 91	9 12	12 08	16 86	7 86	1 523153463	0 043593971	Day 4
Tnni2	3849 63	8436 08	9560 38	7308 08	18228 4	11662 34	1 70267137	0 043602397	Day 4
Cxcl1	8 57	1 91	1 48	1 27	0 88	0 95	0 407754011	0 043638506	Day 4
Egf	3 74	6 89	6 62	6 71	9 2	9 49	1 402469136	0 043684476	Day 4
Cox7a1	151 42	473 07	653 51	499 49	1596 67	498 58	2 027900078	0 043694265	Day 4
Cela3b	1 12	0 29	0	0 32	3 88	0 85	1 825396825	0 043733463	Day 4
Prr29	2 41	3 45	3 43	3 61	6	3 68	1 32546786	0 043760277	Day 4
Gm43802	2 13	3 46	1 22	0 31	0 65	0 76	0 481141692	0 043797298	Day 4
Gm43863	1 97	0 69	0 35	0 4	0 06	0 22	0 612312812	0 043803541	Day 4
Slc2a4	38 04	92 06	155 75	122 93	293 58	129 07	1 899186429	0 043831011	Day 4
Rps6kl1	0 48	0 38	0 61	0 71	0 84	0 64	1 161073826	0 043868781	Day 4
Cd209f	7 46	6 08	4 26	3 96	15 42	8 3	1 475	0 043948017	Day 4
Slc35f3	0 05	0 14	0 14	0 02	0	0	0 906906907	0 044008294	Day 4
Camkv	0 04	0 14	0 08	0 29	0 29	0 1	1 128834356	0 04402305	Day 4
Gzmd	0 98	0 48	1 48	0 48	0	0 07	0 597643098	0 044122377	Day 4
Fads6	1 29	1 68	2 4	2 21	4 91	1 79	1 422939068	0 044144633	Day 4
Asxl3	0 05	0 07	0 03	0 1	0 12	0 07	1 044444444	0 044209044	Day 4
Abcb4	3 3	10 44	13 87	11 31	27 69	14 21	1 836327997	0 044227243	Day 4
8430426J06Rik	0	0 26	0	0 08	1 61	0 25	1 515337423	0 044307057	Day 4
Scn4a	13 79	27 86	37 98	29 59	66 22	40 76	1 68909597	0 044442461	Day 4
Gm26839	0 18	0 48	0 35	0	0	0	0 748129676	0 044524981	Day 4
mt-Co1	8767 35	14789 05	20424 37	19495 59	39528 4	15448 13	1 693240939	0 044564818	Day 4

Fosl1	5 62	1 21	1 12	2 11	0 46	1 98	0 689497717	0 044610619	Day 4
Tc2n	0 01	0 01	0 03	0 13	0 02	0 07	1 055737705	0 044681401	Day 4
Foxf2	0 38	0 31	0 36	0 12	0 07	0 14	0 822222222	0 044833856	Day 4
Acsm5	0 04	0 12	0	0 15	0 18	0 23	1 126582278	0 044875903	Day 4
Gmpr	31 24	74 76	82 47	73 81	146 93	88 49	1 630699326	0 044968389	Day 4
Dtx1	0 47	0 49	0 85	2 8	1 37	0 46	1 586278586	0 044982465	Day 4
Wnt1	0 08	0 05	0	0 32	0 02	0 25	1 146964856	0 044986036	Day 4
Slc25a29	1 88	3 14	4 55	5 57	8 51	2 91	1 590294352	0 045019379	Day 4
Spns2	9 44	7 54	12 05	9 62	25 38	10 23	1 505775835	0 045205074	Day 4
Scn10a	0 13	0 07	0 03	0 02	0	0 02	0 941176471	0 045529242	Day 4
Rassf7	20 98	13 53	16 7	10 96	0 94	5 95	0 384615385	0 045561328	Day 4
Dhh	1 26	1 14	1 89	1 72	4 78	0 99	1 438957476	0 045628554	Day 4
Tmem178b	0 63	0 8	0 99	1 05	1 44	0 7	1 142066421	0 045771797	Day 4
Gm31706	0 05	0 12	0 04	0 24	0 12	0 15	1 093457944	0 045802495	Day 4
Rimklb	0 09	0 01	0 47	0 73	1 27	0 04	1 411764706	0 045999573	Day 4
Ampd1	150 6	225 66	214 91	162 86	376 3	365 39	1 527424811	0 046190916	Day 4
Cldn22	0	0	0	0	1 27	0	1 423333333	0 046351349	Day 4
Crym	1 29	2 67	1 94	2 33	4 96	2 63	1 451685393	0 046361866	Day 4
Tesl1	0	0	0	0 1	0 2	0 05	1 116666667	0 046518343	Day 4
Gm29684	0	0	0	0 07	0 11	0 03	1 07	0 046518343	Day 4
Gm16133	0 17	0	0 11	0	0	0	0 914634146	0 046791738	Day 4
Car4	1 83	1 23	4 38	3 35	10 62	2 01	1 818007663	0 046991933	Day 4
Gm29521	0 96	0 83	0	0 3	0 2	0 79	0 895615866	0 047014902	Day 4
Rspo1	0 62	0 34	0 53	2 22	1 92	0 4	1 679287305	0 047037394	Day 4
P2ry13	6 98	4 39	2 71	2 94	0 41	2 21	0 50117096	0 047092667	Day 4
Gm26569	0 75	0 82	0 38	0 03	0	0 17	0 646464646	0 047113067	Day 4
Kcnn1	4 38	4 74	9 95	7 3	15 07	7 1	1 471227911	0 047369792	Day 4
Idh3b	166 51	296 73	314 24	305 87	582 89	313 9	1 544767323	0 047410902	Day 4
Sec31b	1 46	2 36	4 09	3 74	6 27	3 36	1 500458295	0 047449966	Day 4
Tpsg1	0 07	0	0 12	0 5	0 31	0 27	1 278996865	0 047645283	Day 4
Gm5771	0	0	0	0	0 62	0 35	1 323333333	0 047675028	Day 4
Gm30082	0 18	0 34	0 43	0 51	1 24	0 73	1 387341772	0 047945841	Day 4
Vat1l	1 53	2 13	3	2 83	8 98	1 69	1 708074534	0 048015925	Day 4
Cldn14	0	0	0 03	0 22	0 35	0	1 178217822	0 048142876	Day 4
Dusp26	1 63	3 7	10 91	6 06	28 26	6 13	2 258316008	0 048199365	Day 4
Slc41a3	16 26	25 7	35 08	27 18	62 87	33 01	1 574962519	0 048454699	Day 4
Zdhhc23	1 06	1 31	2 37	2 74	6 47	0 76	1 675710594	0 048463618	Day 4
Pfkm	349 27	641 06	869 64	754 97	1152 66	984 05	1 553798505	0 048571417	Day 4

Tigar	6 83	12 1	19 09	15 02	30 99	18 35	1 642125792	0 048579046	Day 4
Kcnb1	0 93	2 84	2 9	2 7	6 28	3 3	1 580144778	0 048633664	Day 4
Them5	0 05	0 05	0 34	0 6	1 32	0 05	1 444767442	0 048703842	Day 4
Actr3b	1 19	1 19	1 1	1 3	2 49	1 74	1 316358025	0 048764955	Day 4
Gm13292	1 84	1 5	2 76	2 81	3 81	2 82	1 367032967	0 048870676	Day 4
Il12rb1	0 85	0 42	0 26	0 48	0 05	0 19	0 821192053	0 049017898	Day 4
Hmga2	6 76	1 3	0 95	1	0 31	1 3	0 467110741	0 049051791	Day 4
Mtfp1	10 72	30 75	40 77	33 92	88 92	32 78	1 860863444	0 049078406	Day 4
Gm6397	0 39	0 15	0 09	0	0	0	0 826446281	0 049094205	Day 4
Gm12688	0 36	0 22	1 12	1 51	1 82	1 05	1 570212766	0 049150779	Day 4
Gjb1	0 57	0 22	1 17	1 06	3 55	0 4	1 614919355	0 049299397	Day 4
Fam178b	2 85	1	5 31	3 67	16 22	1 84	2 033717105	0 049361048	Day 4
Gm15912	0 35	1 2	0 53	1 95	2 47	0 87	1 631889764	0 049439766	Day 4
Pmp2	1 03	1 24	3 94	3 94	9 49	1 25	1 919652552	0 049689653	Day 4
2210010C04Rik	0	0	0	0	1 4	0 38	1 593333333	0 049709444	Day 4
Vwa3b	0 35	0 37	0 12	0 79	1 67	1 01	1 684895833	0 049753962	Day 4
Lpin1	10 87	21 28	31 82	28 08	53 16	27 57	1 669553531	0 049787359	Day 4
Pde4dip	93 95	206 26	239 77	207 12	404 95	280 99	1 650263361	0 049836427	Day 4
mt-Atp6	71 61	390 85	165 25	365 95	616 95	239 66	1 943143442	0 04990145	Day 4
Cenph	4 59	1 78	1 26	1 65	0 24	0 89	0 54374412	0 049984106	Day 4
Ucp3	56 64	49 28	42 13	111 94	74 67	88 38	1 840383979	1 55394E-15	Day 7
Pdk4	140 43	85 77	74 21	418 11	181 77	274 93	2 893147886	4 61666E-13	Day 7
Arrdc2	23 49	17 18	20 66	70 77	65 24	40 2	2 78579201	5 05893E-11	Day 7
Ppp1r3c	100 72	128 64	109 89	231 99	201 19	201 01	1 861767714	3 10065E-09	Day 7
Ddit4	38 39	28 17	39 03	99 68	97 94	60 89	2 408232802	5 56226E-09	Day 7
Pck1	1 13	1 56	1 7	6 11	2 81	3 93	2 144790257	6 47463E-09	Day 7
Tmem181b-ps	9 35	6 93	6 6	3 56	1 9	3 81	0 474111283	9 01101E-09	Day 7
Tmem254a	39 56	33 86	29 48	6 12	10 9	8 42	0 268555241	5 28375E-08	Day 7
Duxbl1	3 07	1 35	1 9	0 67	0 41	0 41	0 481759657	9 71281E-08	Day 7
Gm20547	1 01	0 39	0 18	0	0	0	0 655021834	1 66169E-07	Day 7
1810049J17Rik	0	0	0	2 92	3 21	4 37	4 5	6 96012E-07	Day 7
Lvrn	1 05	0 94	0 95	2	1 34	1 73	1 358585859	1 18232E-06	Day 7
Cidec	3 36	2 54	4 24	8 52	7 84	6 06	1 934550989	3 75346E-06	Day 7
Nnt	23 55	37 86	23 75	13 18	8 9	11 88	0 41923775	4 39962E-06	Day 7
Slc10a6	1 69	1 49	2 17	4 31	2 69	3 57	1 625149701	5 41063E-06	Day 7
Aldh3a1	0 35	0 41	0 57	1 15	6 15	1 41	2 704387991	8 5581E-06	Day 7
Cd22	4 01	1 36	3 94	1 26	0 37	0 63	0 427294882	9 13246E-06	Day 7
Tmem266	0 3	0 47	0 55	1 27	2 43	1 24	1 837962963	9 1968E-06	Day 7

F5	0 43	0 59	0 55	1 12	1 02	0 92	1 326039387	1 36679E-05	Day 7
Gm26920	0 23	0 51	0 29	0	0	0	0 744416873	1 84044E-05	Day 7
Egr3	2 75	1 7	2 59	0 72	0 48	1 18	0 535856574	2 35668E-05	Day 7
Fgfr3-ps	0	0	0 03	0 94	0 58	0 82	1 762376238	3 25452E-05	Day 7
Alox15	0 85	1 58	1 71	2 93	2 9	3 35	1 705882353	3 56753E-05	Day 7
Gm37385	4 33	10 46	1 32	0	0	0	0 156985871	4 51495E-05	Day 7
Gm10132	24 07	32 25	14 52	85 97	79 82	32 1	2 720612134	4 78564E-05	Day 7
Gm9726	0	0	0	1 25	0 84	0 97	2 02	4 95591E-05	Day 7
Gm16759	0 15	0 13	0 11	0 33	0 4	0 45	1 233038348	0 000140489	Day 7
Zfp968	1 88	2 24	1 52	0 64	0 33	1 28	0 607638889	0 00014753	Day 7
Cyp2e1	9 67	5 48	8 76	20 21	13 36	9 51	1 712374582	0 000302948	Day 7
Gm54741	0 91	0 53	1 48	2 1	2 99	11 5	3 309121622	0 000306215	Day 7
Ces1d	2 81	2 15	2 73	6 59	9 67	4 42	2 21515435	0 000308386	Day 7
Hmgcs2	0 95	1 09	0 87	2 63	1 2	1 5	1 409475465	0 00031049	Day 7
Gm12960	13 23	8 8	4 36	2 91	1 8	3 55	0 383123511	0 000342334	Day 7
Rasd2	1 41	1 73	1 79	3 26	4 14	2 1	1 57629256	0 000589296	Day 7
Gm49355	0 82	0 45	0 72	2 31	0 91	1 23	1 492985972	0 000601225	Day 7
B430218F22Rik	0 28	0 24	0 09	0 7	0 72	0 83	1 454293629	0 000646157	Day 7
Rps27rt	64 62	44 66	55 38	114 41	160 43	64 95	2 044554455	0 00070066	Day 7
Gm3055	2 24	1 63	0 92	0 66	0 19	0 79	0 59563543	0 000775446	Day 7
Gm10241	28 31	36 09	13 36	0	5 23	4 56	0 15837048	0 000813196	Day 7
Gm20735	0 26	0 39	0 68	0	0 24	0 01	0 750577367	0 000946186	Day 7
Gm44421	0 29	0 54	0 54	1 2	2 58	0 97	1 773455378	0 001078106	Day 7
H2-Q1	0 08	0	0	0 21	0 21	0 21	1 178571429	0 001191115	Day 7
Gm15964	0 53	0 52	0 57	0 17	0 1	0 16	0 742424242	0 001320312	Day 7
Gm42501	0	0	0	0 16	0 34	0 58	1 36	0 001526153	Day 7
Il1rn	3 45	5 96	1 01	1 15	0 76	1 06	0 44485842	0 001670026	Day 7
Scn5a	1 6	0 56	0 48	0 77	0 25	0 28	0 762411348	0 00177284	Day 7
Ush1c	0 1	0 16	0 07	0 26	0 41	0 45	1 237237237	0 001905621	Day 7
B3gnt5	0 44	0 59	0 28	0 03	0 08	0 34	0 800464037	0 002012865	Day 7
Alkal2	0 26	0 26	0 27	0 83	0 88	0 61	1 403693931	0 002085675	Day 7
Actn3	1982 29	2259 16	1975 25	2676 79	5324 54	2491 3	1 687481711	0 00209336	Day 7
Nr4a1	45 75	64 86	72 21	78 96	140 25	83 31	1 644171779	0 002129104	Day 7
Tmem160	27 74	23 9	28	29 83	61 72	42 41	1 657308809	0 002144	Day 7
Ppp1ccb	9 42	14 21	15 83	4 04	2 48	8 15	0 416156382	0 002195673	Day 7
Gadd45g	56 42	47 65	92 28	102 98	159 74	86 9	1 768848758	0 002262764	Day 7
Casr	0 55	1 02	0 87	1 12	1 87	1 85	1 441176471	0 002298207	Day 7
Bcl2a1b	17 05	13 11	7 75	6 52	1 69	6 03	0 421412857	0 002447912	Day 7

Sphkap	0 21	0 22	0 18	0 37	0 37	0 35	1 132963989	0 002457528	Day 7
Gm50216	0 36	0 52	0 41	1 06	0 83	0 7	1 303030303	0 002542277	Day 7
Myh4	3182 09	4141 26	3531 87	4568 21	9085 02	4593 84	1 680760751	0 00267511	Day 7
Gm21982	0 1	0 1	0 04	0 14	0 39	0 39	1 209876543	0 00270567	Day 7
Il11ra2	0 1	0 09	0 07	0 39	1 02	0 26	1 432515337	0 002819739	Day 7
Gm47103	0 86	0 58	0 93	0 17	0 06	0 13	0 625698324	0 002896007	Day 7
Cebpb	17 33	10 84	13 18	16 52	33 87	18 07	1 611273957	0 003430473	Day 7
Gm5148	2 78	6 3	5 19	1 7	1 3	2 3	0 4806022	0 003640132	Day 7
Zfp960	1 25	1 88	1 41	0 47	0 13	0 85	0 590185676	0 00392812	Day 7
Angptl4	28 92	14 88	11 27	54 78	13 35	34 44	1 817978302	0 003944776	Day 7
Gm48832	0	0	0	0 07	0 04	0 11	1 073333333	0 0039985	Day 7
Depp1	14 58	15 4	30 77	33 13	42 93	28 96	1 694431373	0 004005191	Day 7
Gm15935	0 5	0 65	0 36	1 5	2 5	2 38	2 079822616	0 00402899	Day 7
A530058N18Rik	4 06	3 35	4 22	4 48	18 76	5 99	2 203007519	0 004200945	Day 7
Zfp991	1 65	0 99	0 72	0 57	0 19	0 44	0 660377358	0 004375812	Day 7
Pmp2	2 58	1 77	2 72	1 02	0 99	0 77	0 573982125	0 004689149	Day 7
Gm47484	0 08	0	0	0 29	0 14	0 18	1 172077922	0 004691051	Day 7
Sox11	2 03	0 71	0 39	0 35	0 17	0 45	0 647634584	0 004834886	Day 7
Gm15737	0 1	0 02	0 04	0 15	0 31	0 12	1 132911392	0 004898829	Day 7
Gm36235	1 72	2 78	3 02	3 23	5 77	3 65	1 487642586	0 004947279	Day 7
Exoc7	26 02	25 34	25 72	30 96	59 79	27 03	1 508241758	0 004982549	Day 7
Adora1	1 28	1 61	0 91	1 21	2 74	2 76	1 427941176	0 005014458	Day 7
Ptgs2	0 71	0 85	0 58	0 24	0 1	0 43	0 733463035	0 005348638	Day 7
Hcar2	0 14	1 88	0 07	0 03	0 08	0 06	0 622789784	0 005899991	Day 7
Ptprn	0 82	0 71	0 32	0 37	0 01	0 14	0 725773196	0 006009975	Day 7
Fbp2	10 47	12 47	10 25	12 53	27 61	14 69	1 597955236	0 006031647	Day 7
Il12a	1 71	2 36	1 5	3 54	5	3 82	1 792298716	0 006221566	Day 7
Gm45706	0	0	0 09	0 24	0 16	0 4	1 229773463	0 006733512	Day 7
Gm46223	5 85	3 2	1 23	0 27	0	1 34	0 347138554	0 007312186	Day 7
Ighg1	0	0 17	0	0 19	0 94	0 51	1 463722397	0 007424313	Day 7
Gm43802	1 59	0 56	1 15	2 68	2 57	2 8	1 753968254	0 007636331	Day 7
Prr14l	0 16	0 05	0 15	0 33	0 18	0 18	1 098214286	0 007691088	Day 7
4932441J04Rik	0 61	0 65	0 41	0 26	0 2	0 22	0 788008565	0 007763626	Day 7
Npcd	0 96	0 95	1 15	0 45	0 33	0 15	0 648514851	0 008009148	Day 7
Trip13	1 69	0 92	0 64	0 6	0 19	0 59	0 7008	0 008288316	Day 7
Bcl2a1a	1 73	2 32	1 45	0 56	0 52	0 59	0 549411765	0 008803777	Day 7
Fgfbp1	1 09	1 02	1 84	1 76	5 73	2 08	1 808633094	0 009916762	Day 7
Rgs6	0 51	0 41	0 25	0 67	1	0 7	1 287769784	0 009992248	Day 7

Gm13781	1 68	0 64	0 62	0 24	0 04	0 35	0 611111111	0 010044986	Day 7
Gm5086	1 74	1 43	1 58	0 51	0 31	0 83	0 6	0 010229492	Day 7
Gm37375	1 71	0	0	12 83	2 36	1 14	4 10403397	0 010474734	Day 7
Gm26912	0	0 04	0	0 21	0 11	0 27	1 180921053	0 010609419	Day 7
Gpx4-ps2	0 6	0 85	0 66	2 38	1 13	2 19	1 702544031	0 01062427	Day 7
Orm1	0 3	0 26	0 48	1 38	0 52	1 8	1 658415842	0 0106668	Day 7
Bach2os	1 03	0 16	0 03	0	0	0	0 710900474	0 010754621	Day 7
Gm45205	0 13	0 08	0 11	0 02	0	0	0 909638554	0 010911035	Day 7
4930516B21Rik	0 7	0 71	0 98	0 25	0 15	0 14	0 6567718	0 011128125	Day 7
Gm44170	1 56	1 68	4 68	0	0	0 98	0 364468864	0 01147272	Day 7
Rxfp3	0 4	0 13	0 1	0 06	0 02	0 04	0 859504132	0 011843426	Day 7
Gm49395	1 3	1 44	1 28	0 82	0 29	0 83	0 703703704	0 012315213	Day 7
Gm9522	1 12	0 62	1 01	0 3	0 14	0 07	0 610434783	0 012344213	Day 7
Gm48128	0 6	0 35	0 23	0 09	0	0 17	0 779904306	0 01255739	Day 7
Car11	1 21	2 11	2 31	2 59	4 3	3 01	1 494785632	0 012605278	Day 7
Gm38560	4 28	4 02	4 66	5 3	9 93	4 39	1 417293233	0 012714241	Day 7
2310015A16Rik	1 13	1 86	1 84	1 38	7 06	3 43	1 899106003	0 01322888	Day 7
Ostn	0 99	2 34	2 72	3 75	2 88	4 14	1 521546961	0 013344196	Day 7
Gm48035	1 43	1 02	1 23	0 51	0 34	0 55	0 658682635	0 013511417	Day 7
Cntnap2	1 46	2 26	1 68	2 26	6 37	1 96	1 617857143	0 013694179	Day 7
AU022793	0 62	0 35	0 56	0 07	0 08	0 21	0 741721854	0 013733212	Day 7
Serpine1	41 66	15 3	17 95	11 91	3 74	16 19	0 447182647	0 013958716	Day 7
Gm48551	1 16	1 51	1 46	0 84	0 8	0 4	0 70687237	0 014129199	Day 7
Spp1	111 63	70 33	21 24	26 49	8 26	21 15	0 285645005	0 014162215	Day 7
Igkv12-46	0	0 34	0 31	4 86	1 02	1 44	2 82739726	0 014267156	Day 7
2310016D23Rik	4 08	4 37	4 41	5 58	9 36	5 31	1 465952081	0 014765874	Day 7
Gcnt4	0 78	0 53	0 28	0 27	0 07	0 23	0 777777778	0 01493035	Day 7
A430093F15Rik	0 42	0 49	0 71	0 05	0 12	0 13	0 714285714	0 015269106	Day 7
Gm28653	15 21	9 27	5 89	4 93	1 48	7 03	0 492658076	0 015594905	Day 7
Cyp1a1	0 32	0 22	0 35	0 39	0 48	0 93	1 233933162	0 015888622	Day 7
Gm6676	0 06	0 18	0 11	0	0	0	0 895522388	0 016086221	Day 7
Depdc1b	0 73	0 53	0 56	0 52	0 2	0 25	0 823651452	0 01640352	Day 7
Sirpb1b	1 5	1 1	1 1	0 78	0 58	0 35	0 702985075	0 016617835	Day 7
Slc1a1	0 34	0 51	0 75	0 73	0 97	0 77	1 189130435	0 016839194	Day 7
1700101I11Rik	0 13	0	0 13	0 18	0 31	0 29	1 159509202	0 017159438	Day 7
Xlr	0 94	0 7	0 84	0 35	0 12	0 13	0 656934307	0 017543187	Day 7
Ccdc112	1 43	1 27	0 7	0 64	0 18	0 95	0 7453125	0 018429706	Day 7
Ighv5-4	0 43	0 52	3 04	0	0	0	0 429184549	0 018865018	Day 7

Gm45705	0 61	1 28	1 1	0 36	0 14	0 58	0 681135225	0 019512641	Day 7
Pkp1	0 54	0 05	0 1	0 04	0	0 06	0 840108401	0 019804362	Day 7
Gm44837	0 17	0 37	0 02	0 6	1 07	1 04	1 603932584	0 019856089	Day 7
Podnl1	2 19	0 56	0 44	0 38	0 05	0 39	0 617124394	0 019915375	Day 7
Gm18180	5 87	4 33	4 27	1	0	0 12	0 235832856	0 020466939	Day 7
Gm48430	0	0	0	0 35	0	0 24	1 196666667	0 020637177	Day 7
Atp13a5	0 18	0 12	0 17	0 27	0 51	0 2	1 146974063	0 021177547	Day 7
Slc25a25	18 96	19 94	34 89	34 9	101 01	39	2 31683813	0 021210492	Day 7
Tlr11	0 41	0 57	0 24	0 17	0 09	0 16	0 81042654	0 021278055	Day 7
Tnf	0 49	0 97	0 54	0 18	0 2	0 2	0 716	0 021378431	Day 7
Inava	0 61	0 1	0 53	0 11	0 03	0 02	0 745283019	0 021850282	Day 7
Gm49477	4 89	3 53	5 66	5 5	12 88	5 43	1 569672131	0 022420635	Day 7
Has2	2 24	1 3	1 58	0 86	0 15	1 1	0 629310345	0 022484815	Day 7
Gm45091	3 21	1 27	1 96	0 62	0 18	0 56	0 461864407	0 023009868	Day 7
Scart1	0 59	0 3	0 33	0 04	0 05	0 16	0 77014218	0 02308942	Day 7
Fut2	0 5	0 21	0 99	0 08	0 46	0	0 753191489	0 023093462	Day 7
Gm48552	0 13	0 2	0 53	0 89	0 87	0 71	1 417098446	0 023169775	Day 7
Inmt	2 36	2 58	7 35	7 05	7 76	7 31	1 642903859	0 023624087	Day 7
1700105P06Rik	0 81	0	0 19	1 21	1 09	0 95	1 5625	0 024301122	Day 7
Slfn4	0 87	1 09	0 56	0 38	0 05	0 56	0 722826087	0 02441866	Day 7
Ctrb1	1 01	0 15	0 07	0	0	0	0 709219858	0 02458706	Day 7
Epha3	3 07	0 99	0 76	0 86	0 19	0 76	0 615089514	0 024880545	Day 7
Pmaip1	4 19	1 55	0 88	1 03	0 46	1 05	0 575883576	0 02563847	Day 7
6330409D20Rik	0	0 11	0	0 53	0 16	0 27	1 273311897	0 025819513	Day 7
C920009B18Rik	0 45	0 33	0 43	0 26	0 1	0 25	0 857482185	0 027111543	Day 7
Tcp10b	0	0	0 03	0	0 24	0 44	1 214521452	0 027974862	Day 7
Mrap	0 84	0 34	1 37	1 66	1 33	1 67	1 38018018	0 028534328	Day 7
Raet1e	0 72	0 48	0 33	0 19	0	0 11	0 728476821	0 028699245	Day 7
Icam4	0 43	0 52	0 41	0 68	0 88	1 41	1 369266055	0 028869548	Day 7
C130080G10Rik	2 45	5 45	4 27	5 38	17 93	4 72	2 045484509	0 029554966	Day 7
Lep	0 29	0 29	0 21	0 69	0 38	0 23	1 134564644	0 029572818	Day 7
Gjd4	0 9	0 23	0 28	0 19	0	0 14	0 755102041	0 029711146	Day 7
Gm18584	0 49	0 09	0 36	0 06	0	0	0 776649746	0 029912605	Day 7
Sim1	0 02	0 01	0 07	0	0	0	0 967741935	0 030312229	Day 7
Mterf1b	0 79	0 78	0 77	0 26	0 21	0 35	0 715355805	0 030372407	Day 7
Rbp1	0 42	0 4	0 14	0 1	0 07	0 07	0 818181818	0 030877051	Day 7
Gm38356	13 16	16 24	12 78	4 27	2 84	11 02	0 467684816	0 030934214	Day 7
Gprin3	0 22	0 16	0 17	0 09	0 05	0 09	0 909859155	0 031126426	Day 7

Gm14493	1 47	1 26	0	0	0	0	0 523560209	0 031361887	Day 7
Gsta5	0	0 15	0	0 12	0 95	0 22	1 361904762	0 031497923	Day 7
Adgb	0 07	0 12	0 07	0 03	0 02	0 01	0 938650307	0 031559034	Day 7
Gm48752	0 08	0 07	0 13	0	0	0 02	0 920731707	0 031694217	Day 7
Tmem196	0 22	0 03	0 09	0	0	0 02	0 904191617	0 03296864	Day 7
6330549D23Rik	0 42	0 15	0 28	0 18	0 05	0 06	0 854545455	0 033250233	Day 7
1810059H22Rik	1 62	1 43	1 17	0 16	0 31	0 87	0 601108033	0 033301346	Day 7
Pou3f1	0 27	0 22	0 29	0 37	0 59	0 34	1 137566138	0 03390762	Day 7
Gm42629	0 05	0 11	0 05	0 24	0 42	0 28	1 22741433	0 03410098	Day 7
5730596B20Rik	0 3	0 16	0 16	0 08	0 04	0 05	0 875690608	0 034443157	Day 7
Aard	0 29	0 17	0 11	0 19	0 71	0 69	1 285714286	0 034486992	Day 7
Gm43075	0 07	0 03	0 03	0 07	0 11	0 2	1 079872204	0 03492962	Day 7
Ighv1-66	1 75	0 6	1 08	0	0	0	0 466562986	0 035978176	Day 7
Gm15283	0 13	0 27	0 3	0 04	0	0	0 821621622	0 036052852	Day 7
Gm11457	0	0	0 1	0 76	1 42	0 23	1 74516129	0 036502542	Day 7
Cxcl5	0 18	1 19	0 06	0 08	0 03	0 11	0 726862302	0 03669561	Day 7
Gm37589	0 01	0	0 01	0 01	0 05	0 13	1 056291391	0 036832192	Day 7
Pcdha5	0	0	0	0 03	0	0 08	1 036666667	0 037040606	Day 7
Gm11752	1 49	3 18	0 96	2 42	8 53	2 55	1 91193511	0 037112848	Day 7
Gm4294	9 17	15 02	0	0	0	0	0 110334682	0 037329391	Day 7
Gm9531	5 32	10 09	7 31	6 63	0 74	1 47	0 460342146	0 037405417	Day 7
Upk1b	0 53	0	0 15	0	0	0	0 815217391	0 037473857	Day 7
Gm45808	0	0 35	0	1 81	0	1 46	1 871641791	0 0377732	Day 7
Gzmb	0 94	1 33	0 85	0 3	0 15	0 68	0 674836601	0 03778731	Day 7
Calcb	0 28	0 2	0 24	0	0	0	0 806451613	0 038196095	Day 7
Btf3-ps18	0 55	1 8	0 55	0 19	0	0	0 540677966	0 038424823	Day 7
Fut1	0 09	0 16	0 09	0	0 02	0	0 904191617	0 038505324	Day 7
Ptchd4	0 19	0 25	0 4	0 11	0 06	0 13	0 859375	0 03854273	Day 7
Rmi2	0 68	0 45	0 78	0 72	1 94	0 6	1 274949084	0 039106641	Day 7
Slc17a8	0 55	0 36	0 15	0 21	0 03	0 08	0 81773399	0 039489222	Day 7
Gm47843	0 12	0 14	0 07	0 32	0 43	0 59	1 303303303	0 0395187	Day 7
C430049B03Rik	4 41	2 52	1 15	2 14	0 68	1 96	0 702166065	0 039666866	Day 7
Gm47147	0 96	0 9	1 05	0 51	0 28	0 42	0 712351946	0 039899496	Day 7
Gm35934	0 09	0 11	0 03	0	0	0 01	0 931888545	0 04026436	Day 7
Itih5l-ps	0	0	0 01	0 03	0 06	0 04	1 03986711	0 040745273	Day 7
Gm11839	0 85	0 51	0 73	0 42	0 11	0 3	0 752455796	0 041279389	Day 7
Pakap	1 8	0 3	0 83	0 04	0 12	0 33	0 588532884	0 041308133	Day 7
Gm11906	0 9	0 68	0 48	0 33	0 08	0 5	0 772727273	0 041499226	Day 7

Gm49601	2 11	1 14	1 55	3 6	1 07	4 5	1 56025641	0 041864921	Day 7
Dcx	1 36	0 65	0 29	0 48	0 1	0 37	0 745283019	0 041943545	Day 7
Uox	1	0 45	0 8	0 1	0	0 14	0 617142857	0 042053571	Day 7
4930512H18Rik	0 86	0 45	0 37	0 18	0	0 25	0 732905983	0 042224801	Day 7
Tnfrsf4	0	0 25	0 34	0 38	0 79	0 93	1 420612813	0 042448666	Day 7
Gm13166	0 68	2 34	0 45	1 7	0 05	1 51	0 967542504	0 042643959	Day 7
Plac9a	143 27	132 89	146 98	23 1	70 12	23 54	0 281034402	0 043024876	Day 7
Ppp1r2-ps4	0 69	0 13	0 5	0	0	0	0 694444444	0 044138681	Day 7
Tnnt2	142 91	55 43	27 44	32 46	12 25	29 38	0 336961273	0 044975873	Day 7
Gm15492	1 21	0 26	0 59	0 88	2 3	1 99	1 614624506	0 0450744	Day 7
Gm49890	1 38	0 5	0 55	0 37	0	0 23	0 662983425	0 045209214	Day 7
Pax1	0 25	0 23	0 19	0 06	0 02	0 1	0 866485014	0 045277288	Day 7
1700087I21Rik	0 18	0 16	0 2	0 16	0	0 12	0 926553672	0 045285578	Day 7
Gm45251	0 32	0 16	0 19	0 04	0	0 04	0 839237057	0 045753186	Day 7
Ighv2-9	0	0	0	2 26	0 76	0 4	2 14	0 0460111	Day 7
Gm42846	0	2 42	0 29	0	0	0	0 525394046	0 047033503	Day 7
Eps8l1	2 54	2 89	2 13	1 86	0 56	0 19	0 53125	0 047320091	Day 7
H3c1	0 33	0 17	0 45	1 13	1 45	0 67	1 582278481	0 04735297	Day 7
Gm47692	0 11	0	0	0 12	0 36	0 22	1 189710611	0 047376273	Day 7
Cnksr2	0 15	0 13	0 19	0 08	0 02	0 02	0 899135447	0 047389076	Day 7
Frmd5	0 37	0 35	0 35	0 08	0 1	0 04	0 791154791	0 047390173	Day 7
Baiap3	0 12	0 32	0 32	0 22	0	0 03	0 864361702	0 04808555	Day 7
Gltpd2	0 24	0 29	0 39	0 05	0	0 09	0 801020408	0 04811389	Day 7
Igkv6-23	0 91	1 81	0 99	3 04	1 8	6 46	2 131147541	0 048246796	Day 7
Gm37420	0 05	0 05	0 08	0	0	0	0 943396226	0 048536538	Day 7
4930481B07Rik	0 73	0 66	0 4	0 12	0 11	0 17	0 709812109	0 048641401	Day 7
Gm17122	0 43	0	0 1	5 08	0 57	0	2 450424929	0 048680662	Day 7
Has1	4 34	2 7	3 72	1 31	0 25	2 78	0 533430233	0 049083404	Day 7
4732471J01Rik	0 1	0 35	0 25	0 03	0	0 02	0 824324324	0 049242579	Day 7
Gm30165	0 01	0	0	0 24	0 03	0 06	1 106312292	0 049365954	Day 7
Odf3l1	0 82	0 75	0 51	0 18	0 33	0 22	0 734251969	0 049412741	Day 7
Plin1	1 41	1 98	1 12	7 5	25 13	18 94	7 266311585	7 83826E-21	Day 14
Tmem45b	0 29	0 31	0 48	2 61	8 79	9 1	5 759803922	1 41204E-20	Day 14
Hp	2 72	7 61	4 82	31 02	34 87	51 35	6 624793388	1 35472E-19	Day 14
Retn	1 07	2 94	1 3	10 68	42 01	26 36	9 873646209	5 55718E-18	Day 14
Mrap	0 18	0 14	0 21	3 81	10 01	5 95	6 450424929	1 74384E-17	Day 14
Pck1	0 81	1 84	1 26	8 8	6 78	16 24	5 039073806	9 3231E-15	Day 14
Slc7a10	0 21	0 22	0 2	1 18	3 73	2 59	2 892561983	5 43553E-12	Day 14

Cfd	41 01	69 98	45 89	267 91	1058 05	797 94	13 30310233	2 17934E-11	Day 14
Arxes2	0 12	0 41	0 36	1 92	4 41	3 34	3 257069409	3 03683E-11	Day 14
Lgals12	0 26	0 18	0 23	0 95	3 15	2 06	2 495912807	7 94839E-11	Day 14
Hcar1	0 07	0 1	0 04	0 57	2 8	0 86	2 252336449	1 37346E-10	Day 14
Adipoq	2 65	4 92	2 2	18 2	72 19	48 91	11 14330462	1 44773E-10	Day 14
Orm1	0 51	1 08	0 15	3 62	15 73	7 94	6 390295359	1 49873E-10	Day 14
A530016L24Rik	0 51	0 34	0 35	1 46	4 07	2 7	2 673809524	8 54981E-10	Day 14
Cyp2e1	2	6	4 18	29 41	119 51	85 91	15 66732543	1 01529E-09	Day 14
Scd1	10 24	17 44	11 13	49 12	171 18	127 68	8 39464243	1 28695E-09	Day 14
Lep	0 04	0 18	0 04	0 54	1 59	1 26	1 960122699	1 70944E-09	Day 14
Slc1a3	1 21	0 73	0 64	1 36	4 26	1 92	1 888888889	1 74778E-09	Day 14
Ces1f	0 34	0 03	0 21	0 77	6 84	2 6	3 689944134	1 88359E-09	Day 14
Acvr1c	0 28	0 05	0 07	0 17	0 63	0 33	1 214705882	3 06062E-09	Day 14
Cidec	1 49	3 67	2 59	12 4	60 83	35 16	10 36186047	3 78347E-09	Day 14
Ptger3	0 19	0 23	0 11	0 75	2 59	1 5	2 220963173	8 25165E-09	Day 14
Mup18	1 27	2 56	2 51	4 46	20 1	25 85	5 718415418	1 05911E-08	Day 14
Alox15	0 1	0 7	0 2	0 85	1 28	0 85	1 495	1 70591E-08	Day 14
Klhdc7a	0 05	0 12	0 07	0 26	0 69	0 63	1 413580247	2 56687E-08	Day 14
Mup10	0 16	0 2	0 49	1 73	5 85	11 66	5 776623377	2 93404E-08	Day 14
Prkar2b	1 03	1 06	0 49	1 63	6 03	3 14	2 47311828	4 12014E-08	Day 14
Gm14434	0 96	1 81	2 04	0 67	0	0 76	0 567221511	1 35727E-07	Day 14
Gsta3	0 21	0 79	0 36	1 05	10 45	2 33	3 860091743	1 50535E-07	Day 14
Car5b	0 74	0 89	0 55	1 43	4 72	2 89	2 324324324	1 67036E-07	Day 14
Slc25a1	7 09	8 25	3 62	11 44	32 57	23 64	3 217213115	1 69655E-07	Day 14
Slc36a2	0 86	1 06	0 55	1 47	7 47	5 2	3 13345521	1 77827E-07	Day 14
Acp5	1 95	4 18	1 21	6 42	16 23	11 59	3 601547389	2 00899E-07	Day 14
Mmd	5 08	5 6	4 19	7 87	13 57	14 01	2 151650811	2 14893E-07	Day 14
Chdh	0 19	0 09	0 11	0 38	0 76	0 83	1 466076696	6 415E-07	Day 14
Cdo1	1 33	2 25	1 7	5 94	31 99	18 75	7 207729469	1 05545E-06	Day 14
Arxes1	0 19	0 17	0	0 78	1 99	1 39	2 130952381	1 68475E-06	Day 14
Mup22	2 06	5 32	3 95	13 63	44 26	63 62	8 688764829	2 0042E-06	Day 14
Smoc1	0 29	0 4	0 29	0 62	1 94	2 09	1 922110553	2 25557E-06	Day 14
Trarg1	0 45	0 78	0 73	1 31	2 56	3 43	2 076612903	2 90391E-06	Day 14
Pnpla3	1 56	1 73	1 41	3 16	3 78	3 41	1 733766234	3 71479E-06	Day 14
Mafb	22 27	21 06	16 42	52 02	52 41	28 23	2 161912351	4 41046E-06	Day 14
Abhd15	0 37	0 27	0 19	1 1	1 18	0 97	1 631853786	7 09874E-06	Day 14
Aacs	2 93	2 25	1 96	4 05	11 07	5 03	2 283037475	8 00937E-06	Day 14
Gm37110	0 84	0 42	0 24	0	0	0	0 666666667	8 80629E-06	Day 14

Pon1	0 21	0 38	0 77	0 68	4 73	3 71	2 779816514	9 12108E-06	Day 14
Tkt	8 91	12 12	7 19	13 81	32 04	23 96	2 332158873	9 85687E-06	Day 14
Cebpd	8 63	8 3	6 81	22 91	15 85	12 74	2 038145101	1 18877E-05	Day 14
Ces1d	6 52	4 02	9 78	7 78	21 74	22 37	2 353773585	1 63256E-05	Day 14
Ankef1	0 07	0 28	0 08	0 45	1 6	0 5	1 618075802	2 40809E-05	Day 14
Slc25a10	4 19	4 09	3 72	5 32	15 23	10 96	2 300666667	2 56332E-05	Day 14
Prr14l	0 25	0 04	0 13	0 33	0 35	0 31	1 166666667	2 61179E-05	Day 14
Mki67	0 13	0 21	0 08	0 4	0 7	0 34	1 298245614	2 9097E-05	Day 14
Nat8l	0 09	0 08	0 05	0 11	0 82	0 56	1 394409938	3 25381E-05	Day 14
Cebpa	5 61	7 55	3 85	8 09	16 1	15 1	2 113443278	4 96008E-05	Day 14
Adrb3	0 12	0 05	0 04	0 27	7 32	1 28	3 697819315	4 97954E-05	Day 14
Fasn	9 85	12 98	7 03	21 39	160 51	65 81	7 629640901	5 51684E-05	Day 14
Apoc1	1 89	2 09	1 84	4	26 54	9 4	4 868480726	5 82964E-05	Day 14
Acaca	4 19	2 94	3 26	4 13	13 52	8 78	2 197908887	5 9686E-05	Day 14
Ccr3	0 09	0 04	0 04	0 26	0 55	0 45	1 34384858	6 28808E-05	Day 14
Tmem179	0 04	0 1	0 02	0 24	0 98	0 56	1 512658228	6 50218E-05	Day 14
Spp1	3 81	5 9	2 17	6 55	13 14	11 4	2 290994624	6 98208E-05	Day 14
Thrsp	22 78	29 84	25 92	32 63	101 27	60 68	2 423105224	7 01908E-05	Day 14
Chst1	1 41	1 92	1 12	2 13	4 41	4 94	1 943624161	8 85207E-05	Day 14
Cd1d1	1 47	1 63	1 99	2 19	6 9	4 92	2 102595797	9 31615E-05	Day 14
Agpat2	8 39	6 89	4 09	8 15	23 28	19 04	2 390254806	0 00010504	Day 14
Gm45819	0 44	0 64	1 03	0 13	0 12	0 1	0 655577299	0 000131818	Day 14
F5	0 09	0 12	0 04	0 23	0 43	0 31	1 221538462	0 000167666	Day 14
Mup14	0	0 29	0 44	0 98	3 68	4 42	3 238605898	0 000188856	Day 14
Adig	6 89	3 22	4 47	9 15	16 44	14 52	2 45221843	0 000226486	Day 14
Aldh3b2	0 02	0 16	0 04	0 16	2 4	0 41	1 854037267	0 000228221	Day 14
Nnt	37 48	21 36	41 48	20 94	14 9	17 47	0 545005807	0 00023086	Day 14
Gm42846	1 82	0 41	2 81	0	0	0	0 373134328	0 000238537	Day 14
Ffar2	0 14	0 05	0	0 48	0 56	0 77	1 507836991	0 000294338	Day 14
Igf2bp2	0 61	0 58	0 45	1 1	1 45	1 15	1 443965517	0 000376313	Day 14
Gys2	0	0	0	0 05	0 43	0 12	1 2	0 000408604	Day 14
H2-Q10	1 5	1 1	0 77	1 72	5 16	7 06	2 659340659	0 000455334	Day 14
Elovl6	0 48	0 38	0 28	0 6	2 02	0 91	1 577294686	0 000497009	Day 14
Rbp4	1 18	3 49	3 06	3 79	13 77	7 63	2 62721342	0 00051204	Day 14
Nnat	0 88	1 1	1 05	6 34	1 08	3 47	2 303482587	0 000562824	Day 14
Gbp2b	0 03	0 03	0	0 12	0 53	0 28	1 284313725	0 000666464	Day 14
Hcar2	0 13	0	0 02	0 28	0 59	0 73	1 46031746	0 000704059	Day 14
Pygl	1 82	2 99	1 39	2 51	8 88	4 83	2 089130435	0 000705524	Day 14

Ctcflos	0	0 04	0 05	0 32	0 53	0 22	1 317152104	0 000878186	Day 14
Gm43305	2 97	1 6	2 58	1 92	0 87	0 78	0 64729064	0 001013755	Day 14
Klb	0 01	0 03	0 05	0 03	0 38	0 5	1 265372168	0 001036725	Day 14
Gpat3	1 47	1 12	1 02	1 55	3 76	2 22	1 593040847	0 00116608	Day 14
Mup19	0 06	0	0 37	0 72	1 74	1 24	1 95335277	0 001239224	Day 14
Gm20489	0	0 11	0	0 9	0 59	0 26	1 52733119	0 001255763	Day 14
Ulbp1	0 49	0 37	0 84	0 72	0 77	0 53	1 068085106	0 001300473	Day 14
Col9a1	0 26	0 06	0 31	0 65	0 72	0 92	1 457300275	0 001420921	Day 14
Cyp26b1	2 9	2 66	1 01	0 7	1 55	0 92	0 644723093	0 001471679	Day 14
Rasd1	0 92	0 61	1 08	1 73	2 12	3 31	1 811051693	0 001549144	Day 14
Plekhh1	0 45	1 14	0 43	0 27	0 41	0 3	0 792828685	0 001565456	Day 14
Xlr3b	0 05	0 3	0 69	2 6	1 37	0 8	1 923267327	0 001614405	Day 14
Nrg4	0 32	0 69	0 55	0 8	3 7	1 47	1 967105263	0 001624716	Day 14
Cyp2f2	1 32	2 37	1 15	2 39	5 06	4 35	1 887755102	0 002033001	Day 14
Gm45470	0	0	0	0 01	0 16	0 03	1 066666667	0 002148618	Day 14
Tgtp1	1 92	1 63	1 21	1 59	4 84	4 81	1 835051546	0 002160093	Day 14
Gm29331	0	0	0	0 9	0 53	1 09	1 84	0 002189038	Day 14
P2rx1	0 36	0 41	0 38	0 68	1 39	1 12	1 491566265	0 002564516	Day 14
Acsm5	0 02	0	0	0 05	0 5	0 11	1 21192053	0 002627492	Day 14
Mgst2	0 41	0 27	0 31	1 39	2 46	2 48	2 338345865	0 002665394	Day 14
Clstn3	0 56	0 48	0 75	0 69	5	3 02	2 444676409	0 002689345	Day 14
Npr3	0 57	0 72	0 77	2 97	1 31	1 09	1 654150198	0 002815394	Day 14
B3galt2	0 38	0 29	0 17	0 46	0 88	0 49	1 2578125	0 003082364	Day 14
Siglecf	0 07	0 46	0	0 4	0 49	0 31	1 1898017	0 003111425	Day 14
Gm43590	0	1 09	0	5 7	0 6	10 49	4 838630807	0 003115467	Day 14
Prc1	0 8	0 78	0 27	2 23	1 75	1 5	1 748453608	0 00322102	Day 14
Gm48832	0	0 03	0 01	0 08	0 49	0 07	1 197368421	0 003311719	Day 14
Gm48778	0	0	0	1	1 73	0 26	1 996666667	0 003340844	Day 14
E230001N04Rik	0 31	0 38	0 38	1 55	0 89	0 58	1 479115479	0 003640269	Day 14
Serpina3c	0 34	1 84	0 46	1 4	4 51	2 58	2 037234043	0 003690274	Day 14
Gm10171	0 04	0	0	0 97	0 3	0 8	1 667763158	0 003931419	Day 14
Cdkn2a	1 39	1 74	1 85	0 7	0 52	0 41	0 580200501	0 004452493	Day 14
Retnlg	0 13	0 25	0 11	1 34	2 84	0 62	2 23495702	0 004861644	Day 14
Kcnf1	0 45	0 38	0 7	4 13	3 26	0 61	2 428256071	0 005046923	Day 14
A730036I17Rik	0 12	0 03	0 12	0 7	0 26	0 15	1 256880734	0 00618027	Day 14
Ifi27l2a	48 96	106 19	38 52	90 9	230 06	287 23	3 10769309	0 006769642	Day 14
Gm14493	0	1	1 76	0	0	0	0 520833333	0 007152012	Day 14
Tnfrsf11b	0 4	0 48	0 35	0 8	0 85	1 06	1 349881797	0 007159579	Day 14

Sell	0 07	0 32	0 08	0 51	0 7	0 57	1 377521614	0 007216451	Day 14
Gm29394	5 54	10 16	4 13	2 68	1 92	2 88	0 459045116	0 007257979	Day 14
Pde3b	0 45	2 69	0 47	1 38	2 05	1 52	1 202723147	0 007262989	Day 14
Gm15915	3 87	0 94	0 84	0 47	0 14	0 28	0 449710983	0 007610596	Day 14
Lonrf3	0 4	0 7	0 91	0 3	0 37	0 32	0 796407186	0 007712805	Day 14
2810459M11Rik	0	0	0	0 04	0 3	0 06	1 133333333	0 007750618	Day 14
Cdsn	0	0 02	0	0 02	0 24	0 21	1 149006623	0 007795201	Day 14
Ly75	0 04	0 04	0 04	0 1	0 15	0 15	1 08974359	0 007866943	Day 14
Rab44	0 8	0 29	0 16	1 65	0 92	0 61	1 454117647	0 00787486	Day 14
Cish	4 66	1 11	1 11	21 2	57 79	2 55	8 556680162	0 007951691	Day 14
Gm49739	0	0 28	0	7 01	13 95	11 12	10 69512195	0 008195116	Day 14
Serpina3g	0 29	0 18	0	0 23	0 72	0 5	1 282420749	0 008206308	Day 14
Actg2	0 74	2 61	1 14	1 5	3 46	5 62	1 813084112	0 00854226	Day 14
Gimap9	0 5	0 85	1 02	1 43	2 26	1 69	1 560521415	0 008592073	Day 14
Gale	0 84	0 95	0 23	1 07	2 7	1 74	1 695219124	0 008783357	Day 14
Timp1	5 33	5 1	1 62	9 23	6 54	10 08	1 916943522	0 008825022	Day 14
Mid1-ps1	4 23	2 83	0 67	1 12	3 72	0 84	0 808946878	0 009396652	Day 14
Fcor	0 59	0	0 51	0 83	6 18	3 76	3 358536585	0 009732317	Day 14
Gm13302	0	0	0	0 23	0 35	0	1 193333333	0 009820835	Day 14
Gm49164	4 03	5 96	2 16	2 25	1 8	2 17	0 608580858	0 010115615	Day 14
Gm17344	0	0	0 12	1 23	0	0 17	1 41025641	0 010185467	Day 14
Zfp936	0	0 13	0	0 37	0 73	0 11	1 345047923	0 010293648	Day 14
Pcsk1n	0 09	0 02	0 02	0 37	0 33	0 15	1 230031949	0 010426838	Day 14
Acsm3	0 44	0 09	0 1	0 48	1 36	1 33	1 699724518	0 010860004	Day 14
Aldh1a7	0 52	0 68	0 79	0 66	3 14	1 65	1 693386774	0 010987685	Day 14
Gm10180	0	0	0	0	0	0	1	0 011068599	Day 14
Uhrf1	0 17	0 39	0 15	0 61	0 78	0 38	1 285714286	0 011196457	Day 14
Opn3	0 52	0 25	0 25	0 58	1 77	0 7	1 504975124	0 0115471	Day 14
Slc6a11	0 09	0 09	0 11	0 01	0 02	0 01	0 924012158	0 011655093	Day 14
Gm13563	0 14	0 51	0 46	0	0	0	0 729927007	0 011962021	Day 14
St6galnac5	0 01	0 03	0 01	0 08	0 14	0 09	1 085245902	0 012024049	Day 14
Nlrc3	0 63	0 73	0 61	2 81	1 17	0 72	1 549295775	0 012625337	Day 14
Gm12185	0 43	0 22	0 18	0 28	1 74	0 66	1 483028721	0 012698871	Day 14
Ccl22	0 11	0 18	0 12	0 59	0 46	0 32	1 281524927	0 013401046	Day 14
Snurf	11 95	2 62	9 33	35 29	32 55	22 99	3 488104089	0 014202599	Day 14
Fa2h	0 76	1 33	0 52	0 84	2 49	2 32	1 541889483	0 015347152	Day 14
Gm36756	0 65	2 38	1 45	0 14	0 22	0 44	0 50802139	0 015611135	Day 14
Lgr6	0 17	0 09	0 07	0 28	0 17	0 15	1 081081081	0 015788787	Day 14

Tmem171	0 2	0 18	0	1 09	0 44	0 37	1 449704142	0 016071097	Day 14
Acpp	0 04	0 07	0 11	0 13	0 35	0 34	1 186335404	0 016493419	Day 14
Pglyrp1	0 4	0 24	0 26	0 85	1 71	1 53	1 817948718	0 016690418	Day 14
Dusp15	0 81	1 45	0 61	0 92	3 58	1 69	1 565587734	0 017153106	Day 14
Muc4	0 09	0 5	0 27	0 13	0 17	0 1	0 880829016	0 017497346	Day 14
Sstr3	0 06	0 15	0 01	0 54	0 28	0 26	1 267080745	0 017510787	Day 14
Gdf1	1 89	0 76	1 15	2 61	2 08	3 64	1 666176471	0 017754077	Day 14
7530428D23Rik	3 94	4 3	10 37	1 59	0 63	1 23	0 298472929	0 017813721	Day 14
Gata1	0 03	0 03	0	0 21	0 45	0 1	1 22875817	0 017833912	Day 14
Hhex	3 65	1 81	1 15	0 64	1 43	1 36	0 669094693	0 017980564	Day 14
Rpl9-ps6	2 5	5 63	1 05	5 25	8 49	7 53	1 992610837	0 01811581	Day 14
Vat1l	1 58	1 16	0 33	0 58	2 6	2 15	1 3723229	0 018412239	Day 14
Irf6	0 08	1 68	0 09	0 64	0 76	0 5	1 010309278	0 018501878	Day 14
Gm11175	0	1 85	2 1	0	0	0	0 431654676	0 018502804	Day 14
Gm12250	0 33	0 41	0 15	0 18	1 58	1 46	1 598971722	0 018962806	Day 14
Mymk	1 26	2 66	0 62	0 57	0 6	0 75	0 652519894	0 019030723	Day 14
Mup9	0 38	0 16	1 26	3 06	7 75	3 57	3 620833333	0 019091094	Day 14
Cnmd	0 04	0	0	0 08	0 61	0 31	1 315789474	0 019554466	Day 14
Gm47996	0	0	0 13	0 49	0 5	0 44	1 415335463	0 019577884	Day 14
Padi4	0	0 14	0 18	0 6	0 57	0 12	1 292168675	0 019697094	Day 14
Gm36927	0	0	0	0 05	0 48	0	1 176666667	0 020424826	Day 14
Mup11	0	0 82	0	2 7	3 55	8 09	4 539267016	0 020499761	Day 14
Gria2	0 02	0	0	0 18	0 02	0	1 059602649	0 020651701	Day 14
Gm47580	0	0	0	0 12	0 15	0 05	1 106666667	0 021194863	Day 14
1810012K08Rik	0 79	0 52	0 18	0	0	0	0 668151448	0 021242944	Day 14
Slc22a3	0 15	0 33	0 26	0 45	0 79	0 52	1 272727273	0 021500645	Day 14
Pfkfb3	55 84	50 74	47 12	87 01	240 46	61 63	2 502233567	0 02157699	Day 14
Msln	0 1	0 32	0 3	0 62	0 87	0 65	1 38172043	0 021705781	Day 14
4930556H04Rik	0 44	0 25	0 38	0 09	0 16	0 03	0 805896806	0 021966117	Day 14
Adtrp	0 11	0 32	0	0 12	0 96	1 08	1 504373178	0 022959814	Day 14
Gm49494	0	0	0 92	3 45	1 77	2 01	2 609693878	0 023036852	Day 14
Krt8	0 21	1 36	0 09	0 18	0 08	0 12	0 725321888	0 023677513	Day 14
Angpt4	0 3	0 38	0 28	0 35	0 89	1 18	1 368686869	0 024271974	Day 14
Ffar4	0 04	0 08	0	0 11	0 2	0 7	1 28525641	0 024300713	Day 14
Gm45632	0	0	0 02	0 23	0 17	0 05	1 142384106	0 024510446	Day 14
Pmp2	1 46	1 63	0 77	0 77	4 51	4 37	1 844023324	0 02491309	Day 14
Prr11	0 14	0 1	0 06	0 21	0 23	0 41	1 166666667	0 024974939	Day 14
Gm9754	0 1	0 08	0 16	0	0 02	0 04	0 916167665	0 025029679	Day 14

Igkv14-111	0	5 63	0 6	0	0 24	0	0 351029252	0 025185945	Day 14
Gm10143	0 07	0 17	0	0	0	0	0 925925926	0 025433452	Day 14
Fam83a	0 07	0	0 01	0 04	0 6	0 43	1 321428571	0 025672645	Day 14
Tll2	0 03	0 02	0 01	0 06	0 15	0 1	1 081699346	0 026494566	Day 14
Ugt8a	0 83	0 97	0 39	0 53	2 63	1 73	1 520231214	0 027600753	Day 14
Coch	0 22	0 35	0 11	0 14	0 64	0 79	1 241847826	0 028298597	Day 14
Kcns3	0 15	0 16	0 04	0 08	0 69	0 55	1 289552239	0 028378941	Day 14
Gvin-ps3	0 01	0	0 01	0 03	0 12	0 02	1 049668874	0 028711887	Day 14
Gm44878	0 39	0 05	0 09	0	0	0	0 849858357	0 028747582	Day 14
Ect2	0 06	0 09	0 05	0 21	0 32	0 17	1 15625	0 028852477	Day 14
Ubd	0	0	0	0 06	0 59	0 19	1 28	0 029474502	Day 14
Serpinb2	0	0 03	0	0 27	0 14	0 02	1 132013201	0 029869028	Day 14
Gm26716	0	0	0	0 77	1 84	0 56	2 056666667	0 030356467	Day 14
Lck	0 32	0 26	0 1	0 15	0 92	0 78	1 317934783	0 030510584	Day 14
Gm2564	2 27	3 49	0 77	2 98	5 76	5 75	1 835257083	0 030756677	Day 14
Acaa1b	0 07	0 13	0 15	0 03	0 94	0 69	1 391044776	0 030921103	Day 14
Ccna2	0 27	0 32	0 17	0 42	0 66	0 58	1 239361702	0 031196831	Day 14
Acat3	0	0 21	0 03	0 06	0 85	0 42	1 336419753	0 03138093	Day 14
H2bc24	0	0	0	0 04	0 1	0 35	1 163333333	0 032486826	Day 14
Prn	0 43	0 43	0 06	0 54	0 47	1 85	1 494897959	0 033084895	Day 14
Gm50470	0	0 05	0 29	0	0	0	0 898203593	0 03366869	Day 14
Evpl	0 01	0 04	0 02	0 12	0 14	0 04	1 074918567	0 033892738	Day 14
Lctl	0 11	0 13	0 03	0 04	1 06	0 4	1 376146789	0 034430958	Day 14
Kcnk3	0 41	0 31	0 27	0 29	0 99	0 86	1 288220551	0 03465564	Day 14
Gm10653	0	0	0	0 05	0 48	0 28	1 27	0 03502001	Day 14
Igfbp2	0 29	0 06	0 07	1 1	0 43	0 52	1 476608187	0 035174294	Day 14
Gm49839	0	0	0	0 04	0 05	0 05	1 046666667	0 035213597	Day 14
Olr1	0	0	0 01	0 05	0 11	0 04	1 063122924	0 035271366	Day 14
Gm4524	0 17	0 32	0 17	0 5	0 64	0 49	1 265027322	0 035852267	Day 14
Kansl2-ps	0 59	0 39	0 13	1 11	0 76	0 87	1 396593674	0 036042208	Day 14
Gm37240	1 4	5 23	0 06	0	0 02	0 4	0 352941176	0 036100765	Day 14
Cxcl9	1 01	1 12	0 77	0 44	5 13	2 18	1 822033898	0 03610614	Day 14
Gm15491	2 39	3 02	3 47	0 55	1 65	2 27	0 628787879	0 037089361	Day 14
Clca3a1	0 06	0 19	0 01	0 24	0 4	0 22	1 18404908	0 037123519	Day 14
Gzmb	0 15	0 04	0 2	0 11	0 95	0 72	1 410029499	0 037149954	Day 14
Erich3	0 01	0 01	0 01	0 07	0 09	0 03	1 052805281	0 037462964	Day 14
9530080O11Rik	0 44	0 49	0 32	0 07	0	0 21	0 771764706	0 037526102	Day 14
Spint1	0 03	0 13	0 04	0 19	0 27	0 32	1 18125	0 038345786	Day 14

Gbp11	0 06	0 04	0 03	0 06	0 69	0 04	1 21086262	0 038545885	Day 14
Crot	0 32	0 19	0 27	0	0 06	0	0 80952381	0 039373839	Day 14
Unc5c	0	0 01	0	0 02	0 02	0 05	1 026578073	0 039936721	Day 14
Fanca	0 21	0 17	0 41	1 26	0 82	0 9	1 577836412	0 040065551	Day 14
Stra6	0 35	0 38	0 17	0 8	0 74	0 97	1 412820513	0 040483954	Day 14
Cyp2c23	3 31	3	3 69	1 1	2 89	0 74	0 594615385	0 040873031	Day 14
Smco3	0 31	0 16	0 23	0 27	1 19	0 57	1 359459459	0 040988681	Day 14
Zbtb32	0 06	0 06	0 08	0 34	0 38	0 24	1 2375	0 042234579	Day 14
Cd3d	0 07	0 06	0 09	0 03	0 92	0 28	1 313664596	0 042339116	Day 14
Samsn1	0 3	0 46	0 07	0 63	0 84	0 61	1 326370757	0 04287569	Day 14
Rpl17-ps5	0	0	0 51	0 85	0 73	2 35	1 974358974	0 043201	Day 14
Gm5148	1 58	2 58	0 87	5 62	2 18	2 7	1 681195517	0 043512201	Day 14
Gm45774	0 26	0 04	0 05	0 38	0 32	0 39	1 220895522	0 043872229	Day 14
Chil1	0 15	0 55	0 24	0 46	0 79	0 82	1 28680203	0 044351025	Day 14
Zfp968-ps	0	1 08	0 21	1 39	1 43	1 26	1 65034965	0 044801669	Day 14
Gm7887	0 08	0 2	2 64	4 21	1 91	5 09	2 400337838	0 045082754	Day 14
Gm42658	0 24	0 23	0 18	0 03	0 07	0 03	0 857534247	0 045300522	Day 14
Wdr86	0 02	0 07	0 06	0 16	0 27	0 13	1 13015873	0 045533463	Day 14
Ipcef1	0 05	0 09	0 04	0 07	0 2	0 07	1 050314465	0 045769864	Day 14
B230206H07Rik	0 22	0 23	0 23	0 32	1 09	0 37	1 298913043	0 046078074	Day 14
Gm15448	0	0 03	0	0 23	0 07	0 05	1 105610561	0 04620536	Day 14
2210039B01Rik	0 7	0 51	0 68	0 21	0 22	0 14	0 73006135	0 046209698	Day 14
Gm45315	3 62	5 04	1 88	0 11	0	1 82	0 364106352	0 046325413	Day 14
Gm37310	0	0 02	0	0 03	0 28	0	1 09602649	0 046349964	Day 14
Zfp781	0 13	0	0 04	0 15	0 33	0 15	1 14511041	0 04680456	Day 14
Trim29	0	0 05	0 02	0 21	0 04	0 19	1 120521173	0 048140379	Day 14
Cntn2	0 04	0 1	0 03	0 01	0 01	0 01	0 955835962	0 048223543	Day 14
Olfr118	0	0	0	0 14	0 09	0 07	1 1	0 049158242	Day 14
Gm29438	0	0 02	0 02	0 09	0 15	0 07	1 088815789	0 049726415	Day 14
Clec9a	0 32	0 23	0 13	0 49	0 76	0 28	1 230978261	0 049754012	Day 14

Supplemental Table 2. Key Reagents

REAGENT	SOURCE	IDENTIFIER
**Antibodies**		
Rabbit monoclonal anti-Gsdme	Abcam	CAT#ab215191, RRID: AB_2737000
Rabbit monoclonal anti-Klf4	Abcam	CAT#ab215036, RRID:AB_2933978
Rabbit monoclonal anti-c-Jun	Abcam	CAT#ab40766, RRID:AB_731602
Rabbit monoclonal anti- PDGFR alpha	Abcam	Cat#ab203491, RRID:AB_2892065
Mouse monoclonal anti-Caspase3	Proteintech	CAT#66470-2-Ig, RRID: AB_2876892
Mouse monoclonal anti-IL-18	Proteintech	CAT#60070-1-Ig, RRID:AB_2280158
Mouse monoclonal anti-Tubulin	Proteintech	CAT#66031-1-Ig, RRID:AB_11042766
Mouse monoclonal anti-Lamin B	Proteintech	Cat#12987-1-AP, RRID:AB_2136290
Mouse monoclona anti-Pax-7	Santa Cruz	Cat#sc-81648, RRID:AB_2159836
Rabbit monoclonal anti-Perilipin-1	Cell Signaling Technology	Cat#9349, RRID:AB_10829911
Mouse monoclonal anti- Myosin Heavy Chain Type IIA	DSHB	Cat#SC-71, RRID:AB_2147165
Mouse monoclonal	DSHB	Cat#BF-F3, RRID:AB_2266724

anti- Myosin Heavy Chain Type IIB		
Rabbit normal IgG (for ChIP-qPCR)	Proteintech	Cat#B900620, RRID:AB_2883054
Mouse normal IgG (for ChIP-qPCR)	Proteintech	Cat#30000-0-AP, RRID:AB_2819035
Mouse Monoclonal anti-LYVE1 (for ChIP-qPCR)	Thermo-Fisher	Cat#14-0443-82, RRID:AB_1633414
Rabbit Polyclonal anti-CD163 (for ChIP-qPCR)	Proteintech	Cat#16646-1-AP, RRID:AB_2756528
Rat monoclonal anti-CD16/32 (TruStain FcX™)	BioLegend	CAT#101320, RRID:AB_1574975
AlexaFluor(R)700 anti-mouse F4/80	BioLegend	CAT#123130, RRID:AB_2293450
Brilliant Violet 605™ anti-mouse CD45	BioLegend	CAT#103155, RRID:AB_2650656
FITCanti-mouse CD11b	BioLegend	CAT#564454, RRID:AB_312788
PerCP/Cyanine5.5 anti-mouse CD11c	BioLegend	CAT#117328, RRID: AB_2129641
APC anti-mouse CD3	BioLegend	CAT#100235, RRID:AB_2561455
APC anti-mouse CD19	BioLegend	CAT#152409, RRID: AB_2629838
APC anti-mouse NK1.1	BioLegend	CAT#108709, RRID:AB_313396
PE anti-mouse Ly6C	BioLegend	CAT#128007, RRID:AB_1186133
PE anti-mouse CD31	BioLegend	CAT#102407, RRID: AB_312902
Brilliant Violet 421™ anti-mouse Ly6G	BioLegend	Cat#127628, RRID:AB_2562567
Brilliant Violet 421™ anti-mouse CD140a Antibody	BioLegend	Cat# 135923, RRID: AB_2814036
APC/Fire™ 750 anti-mouse I-A/I-E (MHC II)	BioLegend	Cat#107652, RRID:AB_2616729

Brilliant Violet 650™ anti-mouse CD192 (CCR2)	BioLegend	Cat#150613, RRID:AB_2721553
BUV496 Anti-Mouse CD86	BD Biosciences	Cat#750437, RRID:AB_2874600
BV786 anti-mouse CD64	BD Biosciences	Cat# 569507, RRID:AB_3685116
Brilliant Ultra Violet™ 661 anti-mouse CD163	Thermo Fisher Scientific	Cat# 376-1631-82, RRID:AB_3074044
LYVE1 Monoclonal Antibody (ALY7) PE-Cyanine7	eBioscience	Cat#25-0443-82, RRID:AB_2802237
Rat IgG1 kappa Isotype Control (eBRG1), PE-Cyanine7	eBioscience	Cat#25-4301-82, RRID:AB_470198
Goat Polyclonal IRDye 800CW Goat anti-mouse IgG antibody	LI-COR Biosciences	CAT#926-32210, RRID:AB_621842
Goat Polyclonal IRDye 800CW Goat anti-rabbit IgG antibody	LI-COR Biosciences	CAT#925-32211, RRID:AB_2651127
Goat Polyclonal Alexa Fluor 488-conjugated anti-rabbit IgG	Thermo Fisher Scientific	CAT#A-11034, RRID:AB_2576217
Goat Polyclonal Alexa Fluor 568-conjugated anti-rat IgG	Thermo Fisher Scientific	CAT#A-11077, RRID:AB_2534121
Goat Polyclonal Alexa Fluor 568-conjugated anti-mouse IgG	Thermo Fisher Scientific	CAT#A-21134, RRID:AB_2535773
InVivoMAb anti-mouse/rat IL-1β (neutralizing antibody)	Bioxcell	CAT#BE0246, RRID:AB_2687727
InVivoMAb anti-mouse IL-18 (neutralizing antibody)	Bioxcell	CAT#BE0237, RRID:AB_2687719

Chemicals, Peptides, and Recombinant Proteins		
BSA	Roche	03117405001
Palmitic acid	Sigma-Aldrich	P9767
Protease inhibitor cocktail	Sigma-Aldrich	P8340
Sirius Red	Sigma-Aldrich	365548
Succinate Dehydrogenase Assay Kit	Sigma-Aldrich	MAK197
Glutamic acid	Sigma-Aldrich	G626
Malic acid/Malate	Sigma-Aldrich	M1000
Pyruvic acid	Sigma-Aldrich	P2256
Succinate	Sigma-Aldrich	S2378
ADP	Sigma-Aldrich	117105
CCCP	Sigma-Aldrich	C2759
Antimycin A	Sigma-Aldrich	A8674
Oligomycin	Sigma-Aldrich	O4876
Rotenone	Sigma-Aldrich	R8875
Digitonin	Sigma-Aldrich	D5628
TMPPD	Sigma-Aldrich	T3134
Sodium L-ascorbate	Sigma-Aldrich	A7631
Methanol	Sigma-Aldrich	67-56-1
Acetonitrile	Sigma-Aldrich	75-05-8
MTBE	Sigma-Aldrich	1634-04-4
Ammonium formate	Sigma-Aldrich	540-69-2
Dichloromethane	Sigma-Aldrich	75-0902
Isopropanol	Sigma-Aldrich	67-63-0
MiR05-Kit	Oroboros	60101-01
Cardiotoxin	Latoxan	L8102-1MG
Kenpaullone	MedChemExpress	HY-12302
T-5224	MedChemExpress	HY-12270
Protein A/G PLUS-Agarose	Santa Cruz Biotechnology	sc-2003
DEPC-treated water	Thermo Fisher Scientific	4387937
DiO dye	Thermo Fisher Scientific	V22886
DAPI	Thermo Fisher Scientific	D1306

Normal goat serum	Thermo Fisher Scientific	31872
Paraformaldehyde	Thermo Fisher Scientific	23-305-510
Fetal bovine serum	Thermo fisher scientific	10099141
Penicillin-Streptomycin	Thermo fisher scientific	15070063
Pre-stained Protein Marker	Thermo fisher scientific	26616
Propidium Iodide Solution	BD Biosciences	556463
RIPA buffer	Beyotime	P0013B
Prestained Protein Marker	GeneTex	GTX50875
Collagenase IV	Worthington	LS004186
DL1000 DNA ladder	Takara Bio	3591A
DL10000 DNA ladder	Takara Bio	3584A
RNAiso Extract Reagent	Takara Bio	9109
Recombinant Human IL-18 Protein	R&D system	9124-IL
Cenicriviroc	Biorbyt	orb402001
**Critical Commercial Assays**		
CD11b MicroBead Kit	Miltenyi Biotec	130-097-142,
PDGFRα MicroBead Kit	Miltenyi Biotec	130-101-502
Seahorse XF Real-Time ATP Rate Assay Kit	Agilent	103592-100
PrimeScript™ RT-PCR Kit	Takara Bio	RR014A
Vectastain Elite ABC-HRP Kit (Mouse)	Vector Laboratories	PK-6102
Nuclear and Cytoplasmic Protein Extraction Kit	Beyotime Biotechnology	P0028
BCA protein assay kit	Beyotime Biotechnology	P0011

AxyPrep DNA GelExtraction Kit	Axygen Biosciences	#AP-GX-250
**Deposited data**		
RNA-seq on muscle from WT and Gsdme-KO mice injected CTX	This paper	GSE271087
Single cell RNA-seq on muscle from WT and Gsdme-KO mice injected CTX	This paper	GSE270614
Lipidomics on muscle from WT and Gsdme-KO mice injected CTX	This paper	MTBLS10509
**Experimental Models: Organisms/Strains**		
Mouse: C57BL/6J	Shanghai Sino-British SIPPR/BK Lab Animal Ltd.	N/A
Mouse: *B6.129P2-Lyz2$^{tm1(cre)Ifo}$/J*	Jackson Laboratory	JAX stock 004781
Mouse: C57BL/6-Tg(Pdgfra-cre)1Clc/J	Jackson Laboratory	JAX stock 013148
Mouse: Gsdme-KO	Shanghai Biomodel Organism Science & Technology Development Co., Ltd.	N/A
Mouse: Gsdme$^{stop/stop}$	Cyagen Bioscience Inc.	KICMAS190710LY2
**Oligonucleotides**		
qPCR		
Primers for mouse Gsdma Forward	Sangon Biotech	CGGGTCTGTCACGGAGCA
Primers for mouse Gsdma Reverse	Sangon Biotech	CAGCCCTTGGGGATGGTTA

Primers for mouse Gsdmc Forward	Sangon Biotech	TTGTCAAGGTTGGAGGCAGTG
Primers for mouse Gsdmc Reverse	Sangon Biotech	AGTTCAGCCAGTAGCCGTGTT
Primers for mouse Gsdmd Forward	Sangon Biotech	CTTTATGCTTGAAGGGTGA
Primers for mouse Gsdmd Rorward	Sangon Biotech	TGCTGCCGCTTACCTCC
Primers for mouse Gsdme Forward	Sangon Biotech	GCAACGGAGGATGGGACG
Primers for mouse Gsdme Rorward	Sangon Biotech	GGGCAGGTAACACCGCAAA
Primers for mouse *Il-1β* Forward	Sangon Biotech	CAGGCTCCGAGATGAAC
Primers for mouse *Il-1β* Reverse	Sangon Biotech	TGCTTGTGAGGTGCTGA
Primers for mouse Il-18 Forward	Sangon Biotech	TTATTGACAACACGCTTTAC
Primers for mouse Il-18 Reverse	Sangon Biotech	TCTGATTCCAGGTCTCC
Primers for mouse *Pax7* Forward	Sangon Biotech	GTTCGGGAAGAAAGAGGACGAC
Primers for mouse *Pax7* Reverse	Sangon Biotech	GGTTCTGATTCCACATCTGAGCC
Primers for mouse *Pax3* Forward	Sangon Biotech	GCGTCTCTAAGATCCTGTGCAG
Primers for mouse *Pax3* Reverse	Sangon Biotech	GATTTCCCAGCTAAACATGCCCG
Primers for mouse *Myf5* Forward	Sangon Biotech	GGTGGAGAACTATTACAGCCTGC
Primers for mouse *Myf5* Reverse	Sangon Biotech	ACAGTAGATGCTGTCAAAGCTGC
Primers for mouse *Gapdh* Forward	Sangon Biotech	CCCATCACCATCTTCCAGGAG
Primers for mouse *Gapdh* Reverse	Sangon Biotech	TTCACCACCTTCTTCTTGATGTCAT
Chip(Jun)		
Primers for mouse *Cd163* Forward	Sangon Biotech	ACGGCTGGAGCATGAATGAA
Primers for mouse *Cd163* Reverse	Sangon Biotech	TCTTCCTAAGCATCGGTGGC
Primers for mouse *Lyve1* Forward	Sangon Biotech	GCCAACGAGGCCTGTAAGAT
Primers for mouse *Lyve1* Reverse	Sangon Biotech	GGAGTTAACCCAGGTGTCGG
Chip(Klf4)		

Primers for mouse *Cd163* Forward	Sangon Biotech	AGCTTTGGAATGGGTGGACA
Primers for mouse *Cd163* Reverse	Sangon Biotech	GGAGCGTTAGTGACAGCAGA
Primers for mouse *Lyve1* Forward	Sangon Biotech	CCGGAGGGATCTGCACAATG
Primers for mouse *Lyve1* Reverse	Sangon Biotech	TCTGTTGCGGGTGTTTGAGT
**Software and Algorithms**		
GraphPad Prism version 8	GraphPad software	https://www.graphpad.com/
FlowJo v.10	BD Biosciences	https://www.flowjo.com/
FlowAi-2.3.2	BD Biosciences	https://www.flowjo.com/exchange/
UMAP-4.1.1	BD Biosciences	https://www.flowjo.com/exchange/
ImageJ	National Institutes of Health	https://imagej.nih.gov/ij/
FV10-ASW Viewer software	Olympus	https://www.olympus-lifescience.com.cn
HALO	Indica Labs	https://indicalab.com/halo
ProteoWizard	ProteoWizard software	https://proteowizard.sourceforge.io/
R-3.6.0	R Core Team	https://www.r-project.org/
Seurat-4.3.0	R package	https://satijalab.org/seurat/
Mfuzz-2.64.0	R package	DOI: 10.18129/B9.bioc.Mfuzz
CellChat-1.6.1	R package	https://github.com/sqjin/CellChat
Monocle-2.26.0	R package	https://cole-trapnell-lab.github.io/monocle-release/
QuSAGE-2.38.0	R package	DOI: 10.18129/B9.bioc.qusage
Python-3.8.10	Python Core Developers	https://www.python.org/
Scanpy-1.9.3	Python package	https://scanpy.readthedocs.io/en/stable/
Scipy-1.10.1	Python package	https://scipy.org/
pySCENIC-0.12.1	Python package	https://github.com/aertslab/pySCENIC
scVelo-0.2.5	Python package	https://scvelo.readthedocs.io/en/stable/#
Cellpose -3.0	Python package	https://github.com/MouseLand/cellpose
