# Supplementary material for: GSDME–IL-18 pyroptotic axis prevents myosteatosis by expanding tissue-resident macrophages to promote muscle regeneration
Source: J Clin Invest. 2026 Feb 17;136(8):e198076. doi: 10.1172/JCI198076 (PMC13078871; doi:10.1172/JCI198076)
Supplement: Unedited blot and gel images [file jci-136-198076-s278.pdf]

Full unedited gel for Figure F1E

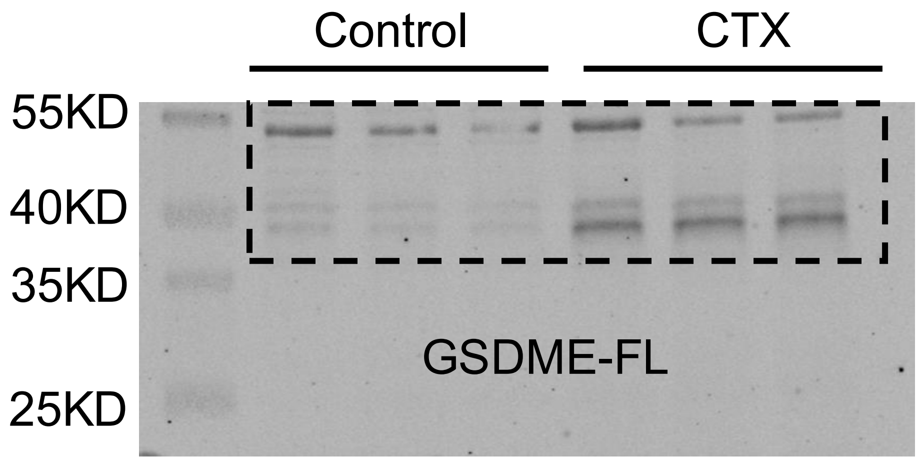

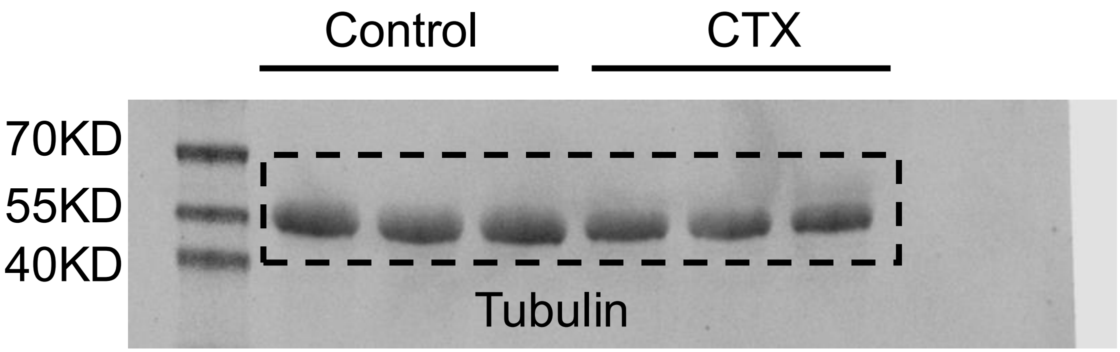

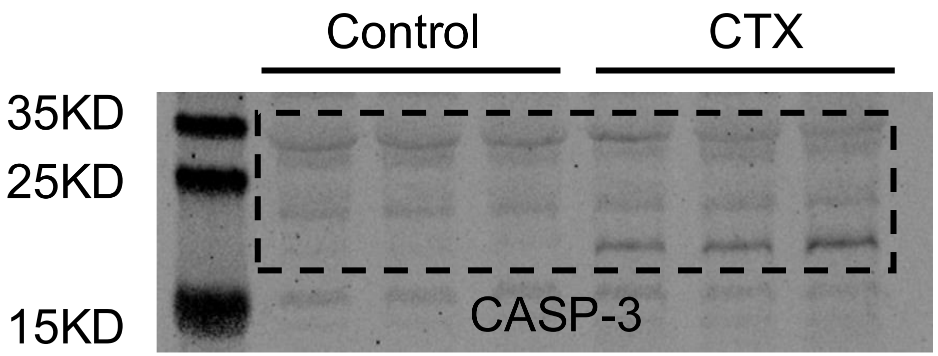

Full unedited gel for Figure F6J

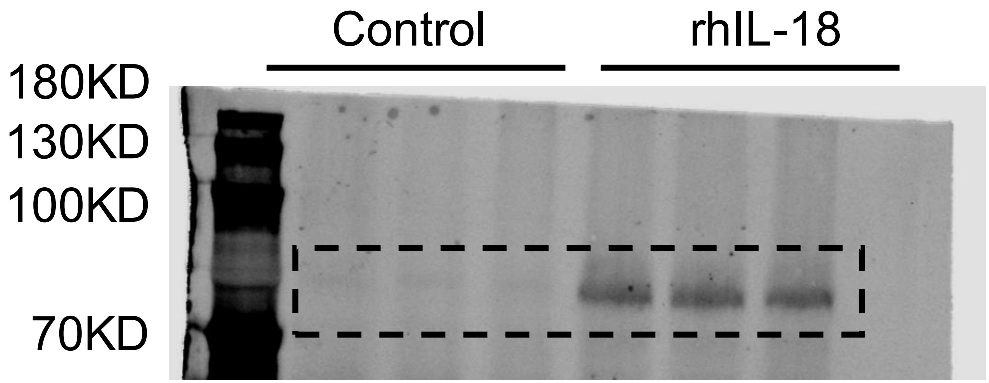

Klf4

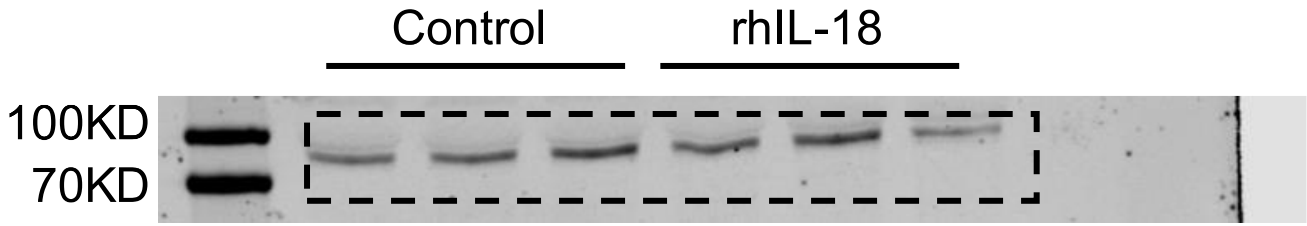

Lamin B

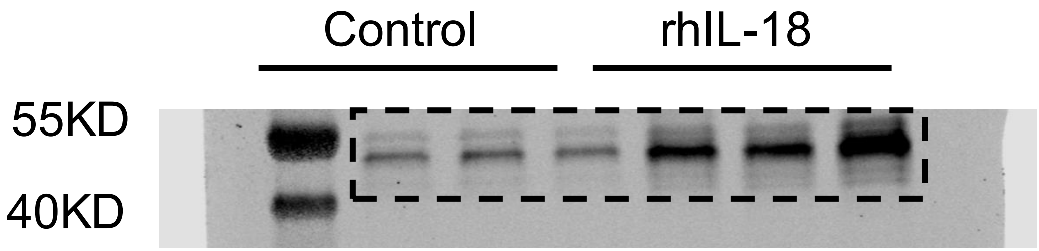

Jun

Full unedited gel for Figure S2B

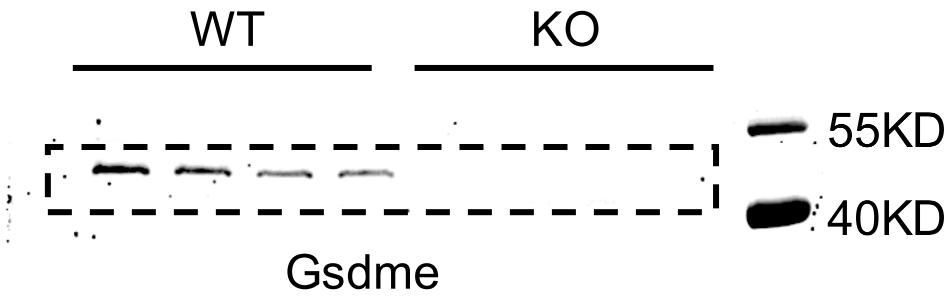

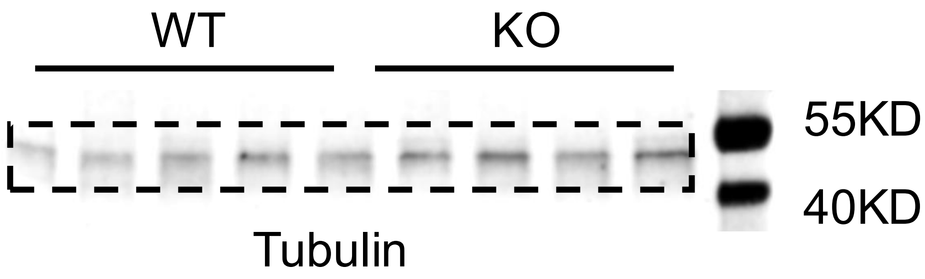

Full unedited gel for Figure S6F

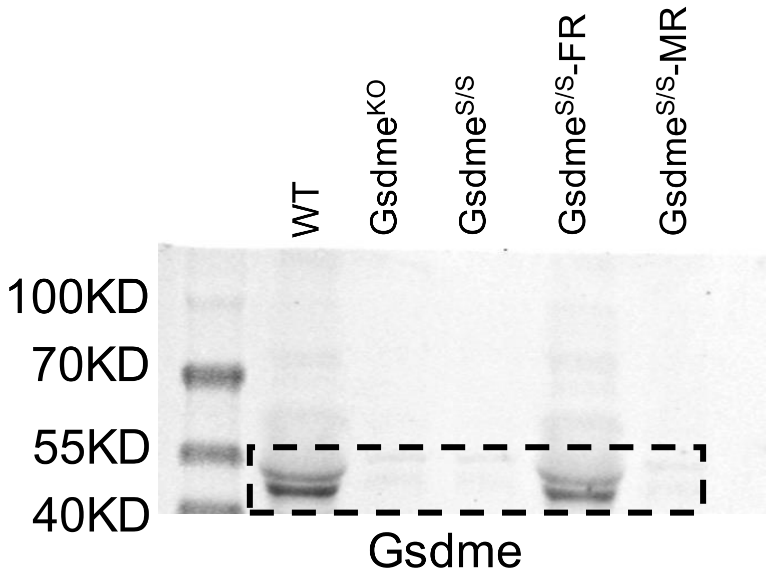

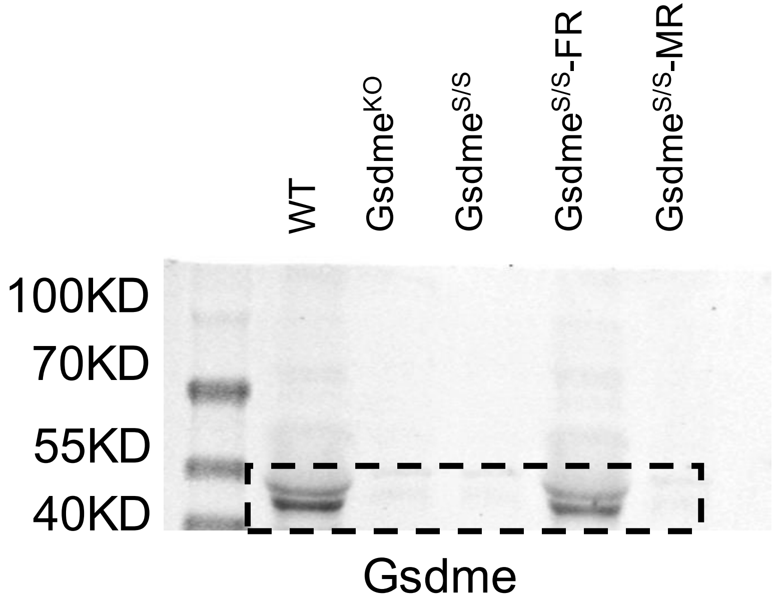

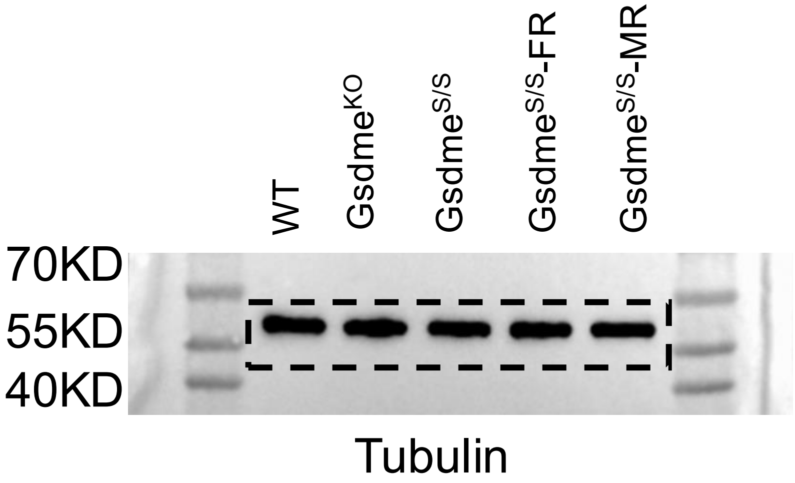

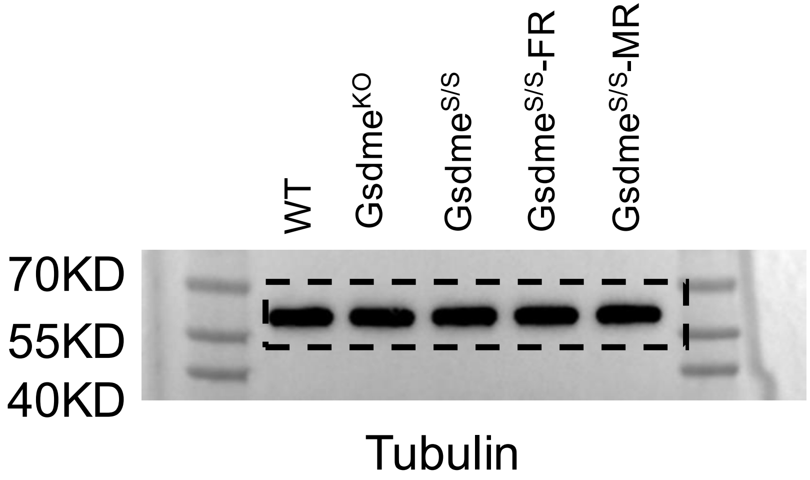

Full unedited gel for Figure S10D

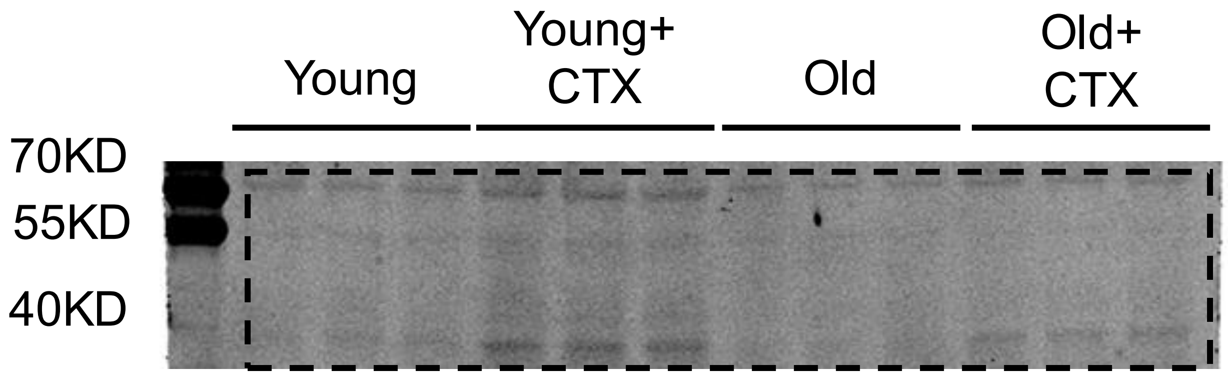

GSDME

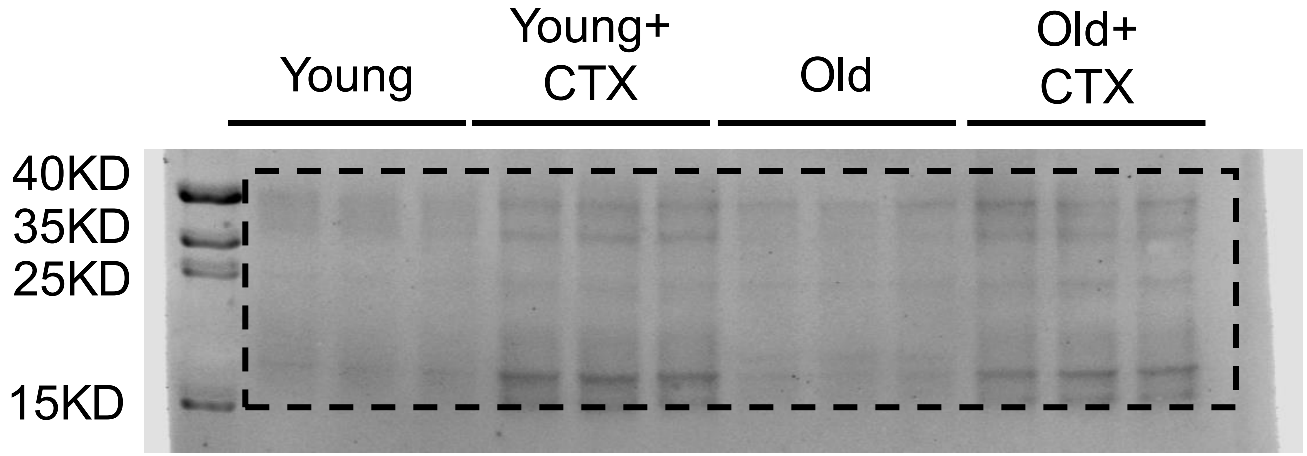

IL-18

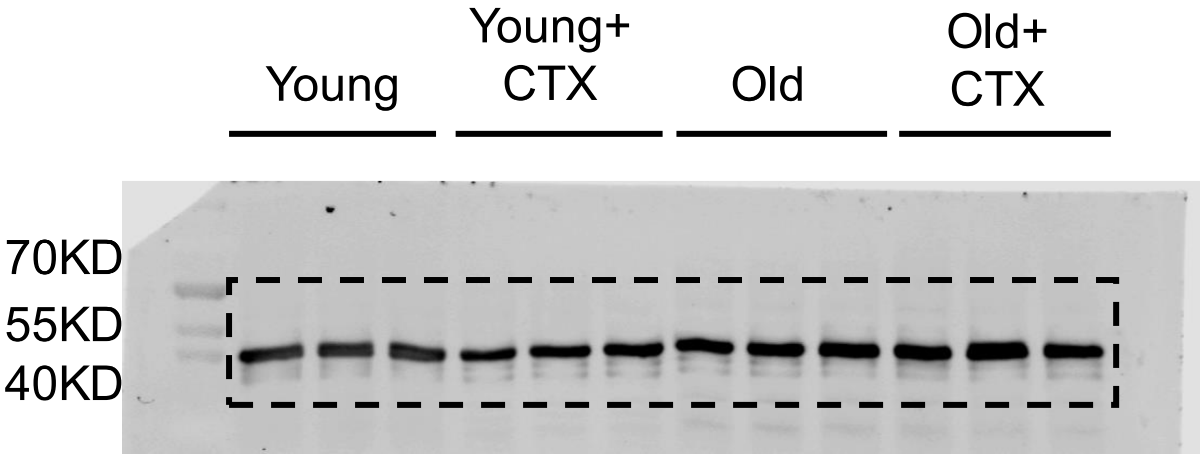

Tubulin
